# BTK and PI3K Inhibitors Reveal Synergistic Inhibitory Anti-Tumoral Effects in Canine Diffuse Large B-Cell Lymphoma Cells

**DOI:** 10.3390/ijms222312673

**Published:** 2021-11-24

**Authors:** Weibo Kong, Sina Sender, Leila Taher, Simon Villa-Perez, Yixuan Ma, Anett Sekora, Barbara C. Ruetgen, Bertram Brenig, Julia Beck, Ekkehard Schuetz, Christian Junghanss, Ingo Nolte, Hugo Murua Escobar

**Affiliations:** 1 Department of Medicine Clinic III, Hematology, Oncology and Palliative Medicine, Rostock University Medical Center, 18057 Rostock, Germany; weibo.kong@outlook.com (W.K.); Sina.Sender@med.uni-rostock.de (S.S.); Simon.VillaPerez@med.uni-rostock.de (S.V.-P.); Yixuan.Ma@med.uni-rostock.de (Y.M.); anett.sekora@med.uni-rostock.de (A.S.); christian.junghanss@med.uni-rostock.de (C.J.); 2 Small Animal Clinic, University of Veterinary Medicine Hannover, 30559 Hannover, Germany; ingo.nolte@gmx.net; 3 Institute of Muscle Biology and Growth, Research Institute for Farm Animal Biology (FBN), 18196 Dummerstorf, Germany; 4 Institute for Biostatistics and Informatics in Medicine and Ageing Research, Rostock University Medical Center, 18057 Rostock, Germany; leila.taher@tugraz.at; 5 Institute of Biomedical Informatics, Graz University of Technology, 8010 Graz, Austria; 6 Department for Pathobiology, Clinical Pathology, University of Veterinary Medicine Vienna, Veterinärplatz 1, 1210 Vienna, Austria; Barbara.Ruetgen@vetmeduni.ac.at; 7 Department of Animal Sciences, Faculty of Agricultural Sciences, University of Goettingen, 37077 Goettingen, Germany; bbrenig@gwdg.de (B.B.); ekkehard.schuetz@agr.uni-goettingen.de (E.S.); 8 Chronix Biomedical Goettingen, 37079 Goettingen, Germany; jbeck@chronixbiomedical.de

**Keywords:** BCR signaling pathway, BTK inhibitor, PI3K inhibitor, DLBCL, gene variants

## Abstract

Bruton’s tyrosine kinase (BTK) and phosphoinositide 3-kinase (PI3K) in the B-cell receptor (BCR) signaling pathway are considered potential therapeutic targets for the treatment of B-cell lymphomas, among which, diffuse large B-cell lymphoma (DLBCL) is the most common type. Herein, we comparatively evaluated the single and combined application of the BTK inhibitor ibrutinib and the selective PI3Kγ inhibitor AS-605240 in the canine DLBCL cell line CLBL-1. For further comparison, key findings were additionally analyzed in canine B-cell leukemia GL-1 and human DLBCL cell line SU-DHL-4. While ibrutinib alone induced significant anti-proliferative effects on all cell lines in a dose-dependent manner, AS-605240 only induced anti-proliferative effects at high concentrations. Interestingly, ibrutinib and AS-605240 acted synergistically, reducing cell proliferation and increasing apoptosis/necrosis in all cell lines and inducing morphological changes in CLBL-1. Moreover, the combined application of ibrutinib and AS-605240 reduced relative phosphorylation and, in some instances, the levels of the BTK, AKT, GSK3β, and ERK proteins. Comparative variant analysis of RNA-seq data among canine B- and T-lymphoid cell lines and primary B-cell lymphoma samples revealed potentially high-impact somatic variants in the genes that encode PI3K, which may explain why AS-605240 does not singly inhibit the proliferation of cell lines. The combination of ibrutinib and AS-605240 represents a promising approach that warrants further in vivo evaluation in dogs, potentially bearing significant value for the treatment of human DLBCL.

## 1. Introduction

The past decades have witnessed an increase in the incidence of lymphoma in humans and dogs. Today, lymphoma is among the most common malignancies in both species [1]. Due to similarities in molecular biology, treatment, and outcome, canine B-cell lymphoma is considered as a spontaneously arising animal model of human non-Hodgkin lymphoma (NHL) [2,3]. In dogs, diffuse large B-cell lymphoma (DLBCL) is the most frequent B-cell lymphoma; the survival of patients in absence of treatment is limited to a few weeks or months [4]. Conventional first-line therapeutic interventions are CHOP (cyclophosphamide, hydroxydaunorubicin, vincristine, prednisolone)-based protocols similar to those used in humans. While 85–90% of canine patients respond to CHOP, most of them relapse and develop resistance to the initial therapeutic protocol within 12 months, mostly with fatal outcome [5].

Tyrosine kinase inhibitors (TKIs) are next-generation cancer agents that allow highly specific targeting of key kinases in lymphoma-relevant pathways, such as the B-cell receptor (BCR) pathway. Among them, Bruton’s tyrosine kinase (BTK) and phosphoinositide 3-kinase (PI3K) inhibitors have exhibited encouraging effects on human and canine leukemia and lymphoma. In the activated B-cell-like (ABC) subtype of DLBCL (ABC-DLBCL), Davis et al. showed that a chronically activated BCR pathway was essential for cell survival [6]. In particular, BTK was crucial for the survival of ABC-DLBCLs with wild-type CARD11. Moreover, in a phase I/II clinical trial assessing the BTK inhibitor ibrutinib as a single treatment in 80 relapsed or refractory human DLBCL patients, 37% of ABC-DLBCL patients responded to the treatment, whereas only 5% of the patients with the germinal center B-cell subtype of DLBCL (GCB-DLBCL) did [7]. This study emphasizes the importance of BTK and the molecular subtype when targeting the BCR pathway. BTK inhibitors have also been reported to be effective at treating canine B-cell lymphoma. Thus, Honigberg et al. indicated that ibrutinib induced partial responses in three of eight dogs [8]. Moreover, the administration of another BTK inhibitor, acalabrutinib, in canine B-cell lymphoma in vitro and in vivo models resulted in dose-dependent inhibition of BTK autophosphorylation and clinical benefit in 30% of the dogs [9].

In order to target PI3K, currently, different approaches using pan- and isoform-specific PI3K inhibitors are being evaluated. Accordingly, Bojarczuk et al. compared molecules targeting different isoforms of class I PI3K in human DLBCL cell lines and showed that most of the analyzed cell lines were more sensitive to a pan-PI3K inhibitor (pictilisib) than to selective PI3K-α, -β, -δ and dual -β/δ inhibitors [10]. However, one PI3K α/δ inhibitor (copanlisib) was highly effective in most of these DLBCLs. In another study, Scuoppo et al. noted that pan-PI3K inhibitors may cause deregulated PI3K activity in phosphatase and tensin homolog (PTEN)-negative DLBCLs [11]. In addition, selective PI3K-δ/γ inhibitors could suppress the AKT S473 phosphorylation in dasatinib-resistant cell lines. Therapeutic targeting of the PI3K pathway has also been reported for the canine B-cell lymphoma cell line 3132, in which exposure to the pan-class I PI3K inhibitor ZSTK474 induced antiproliferative effects [12]. Furthermore, the selective PI3Kδ inhibitor RV1001 was evaluated in 21 dogs with B- and T-cell lymphoma, resulting in response rates of 62% and a median time to progression of 21 days [13].

While there are studies about the combined application of these inhibitors in humans, a comparable experimental setting has not been reported in dogs. Taking into account the high comparability of canine and human lymphoma in terms of presentation, biologic response, and molecular mechanisms, dogs constitute highly valuable naturally occurring models to evaluate innovative therapeutic protocols. Further, the genetic diversity of canine breeds and their stable genetic backgrounds offer the possibility to comparatively analyze pathogenic lymphoma mechanisms and therapeutic interventions in genetically independent populations. Finally, the rapid relapse of canine lymphoma patients enables the evaluation of second-line protocols in substantially shorter time spans than in humans. Concerning human therapeutic approaches, the combined application of BTK and PI3Kδ inhibitors has proved to be superior compared to single-agent treatments in transgenic murine CLL model [14,15]. Recent studies have revealed that the combination of ibrutinib and idelalisib induces apoptosis of ibrutinib-resistant human cell lines by downregulating AKT and restoring FOXO3a levels. The drug-resistant cell lines presented downregulation of FOXO3a/PTEN levels and activation of AKT compared to the sensitive counterparts [16]. Accordingly, and taking into account the comparative oncologic aspects, the combined application of BTK and PI3K inhibitors in the treatment of canine DLBCL bears significance for dogs as patients and as an in vivo model for humans with DLBCL.

Single nucleotide variants (SNVs) affecting *BTK* as well as *PI3K* genes have been described to mediate resistance against ibrutinib (e.g., C481S in *BTK*) [17,18] and dactolisib (e.g., H1047R in *PIK3CA*) [19]. Accordingly, the genomic background is expected to have a non-negligible influence on inhibitor efficacy and clinical outcome. Herein, we characterized the effects of ibrutinib and AS-605240 as single and combined agents in canine and human pre-clinical DLBCL models at the cell biological level. Further, we analyzed the sequences of the *BTK*, *PI3K*, and *PTEN* genes for SNVs and compared them across several canine B-, T-lymphoid cell lines and a set of canine primary B-cell lymphoma samples searching for potentially high-impact variants in the genes that encode the primary targets of ibrutinib and AS-605240. The collected data represent a solid basis for later in vivo evaluation of combinations of AS-605240 and ibrutinib in dogs with spontaneous lymphoma.

## 2. Results

### 2.1. Single Treatment of Ibrutinib Exhibits Anti-proliferative Effects on CLBL-1, GL-1, and SU-DHL-4

**Figure 1:**
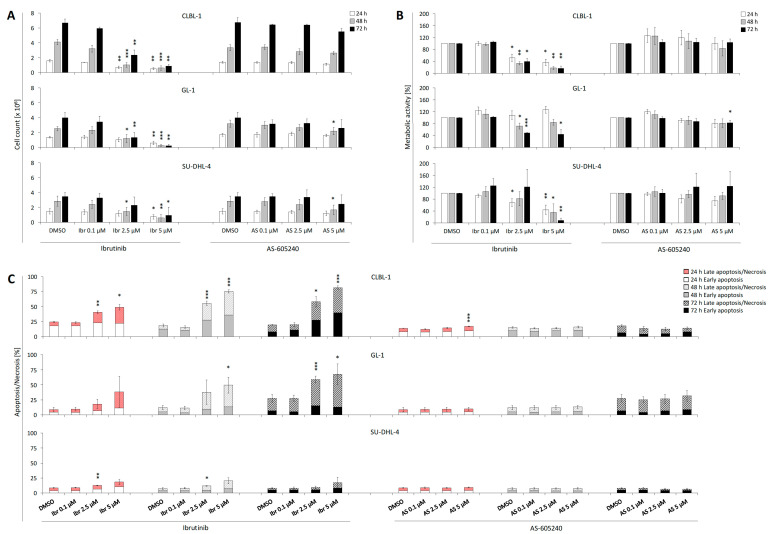
CLBL-1, GL-1, and SU-DHL-4 exposed to ibrutinib or AS-605240 separately at various concentrations (0.1–5 μM) for 24, 48, and 72 h. (**A**) The number of viable cells. (**B**) The metabolic activity (indicated as the percentage compared to the DMSO-treated cells). (**C**) The rate of early apoptosis (annexin V+/PI-) and late apoptosis/necrosis (annexin V+/PI+). Percentages are shown as relative to alive cells were first gated based on FSC/SSC. The figures display the average value (±SD) of three independent experiments. The significance of the treatment effect compared to the control was calculated as a *p* value using Student’s *t*-test. When * *p* < 0.05, then **; *p* < 0.01, ***; *p* < 0.001.

Compared to the DMSO control, ibrutinib showed anti-proliferative effects on CLBL-1 beginning at 1 µM at all time points, while the reference cell lines GL-1 and SU-DHL-4 showed significant (*p* < 0.05, two-tailed *t*-test) reduction at 2.5 µM at selective time points (Figure 1A, Supplementary Table S1A–C). Exposure to 5 µM ibrutinib led to a significant (*p* < 0.05, two-tailed *t*-test) reduction in the number of vital cells in all cell lines at all time points. No inhibitory effect was observed on the proliferation of any of the three cell lines for concentrations of ibrutinib lower than 0.1 μM. Based on the anti-proliferative effect of ibrutinib concentrations on CLBL-1 at 72 h, the IC50 was calculated as 1.3 μM. Following this, 1 µM (IC45) was chosen as the fixed concentration of ibrutinib for the combined application experiments. AS-605240 showed inhibitory effects on CLBL-1 only at a concentration of 10 μM (Figure 1A, Supplementary Table S1A). AS-605240 reduced GL-1 and SU-DHL-4 proliferation at a concentration of 5 μM and an incubation time of 48 h (Supplementary Table S1B,C).

In addition, ibrutinib exerted inhibitory effects on the metabolic activities of CLBL-1 and reference cell lines GL-1 and SU-DHL-4. These effects on the tested cell lines were evident starting at a concentration of approximately 1–2.5 μM, with variations at specific time points (Figure 1B, Supplementary Table S2A–C). No inhibitory effects were observed in any of the three cell lines for concentrations of ibrutinib lower than 0.1 μM. AS-605240 showed inhibitory effects on the metabolic activity of CLBL-1 at concentrations of 10 μM at 24 and 72 h (Figure 1B, Supplementary Table S2A–C). Concerning the reference cell lines, AS-605240 also showed an inhibitory effect on GL-1 only at a concentration of 5 μM at 72 h. This effect was not observed for SU-DHL-4.

### 2.2. Single Treatment of Ibrutinib Induces Apoptosis and Necrosis of CLBL-1, GL-1, and SU-DHL-4

Ibrutinib increased early apoptosis and late apoptosis/necrosis for CLBL-1 and selectively for reference cell lines GL-1 and SU-DHL-4 beginning at concentrations of 2.5 μM (Figure 1C). Compared to the control, 5 μM ibrutinib significantly increased CLBL-1 apoptosis/necrosis from 19.3% up to 81.5% (Supplementary Table S3A). GL-1 and SU-DHL-4 showed a corresponding increase from 27.6% to 67.7%, and from 7.4% to 20.4%, respectively (Supplementary Table S3B,C).

AS-605240 concentrations from 5 μM on increased apoptosis/necrosis of CLBL-1 significantly for selective time points (Figure 1C, Supplementary Table S3A). In GL-1 and SU-DHL-4, no significant increase of apoptotic/necrotic cells was observed (Figure 1C, Supplementary Table S3B,C).

### 2.3. Combined Application of Ibrutinib and AS-605240 Enhances Inhibition of CLBL-1 Proliferation and Metabolic Activity

**Figure 2:**
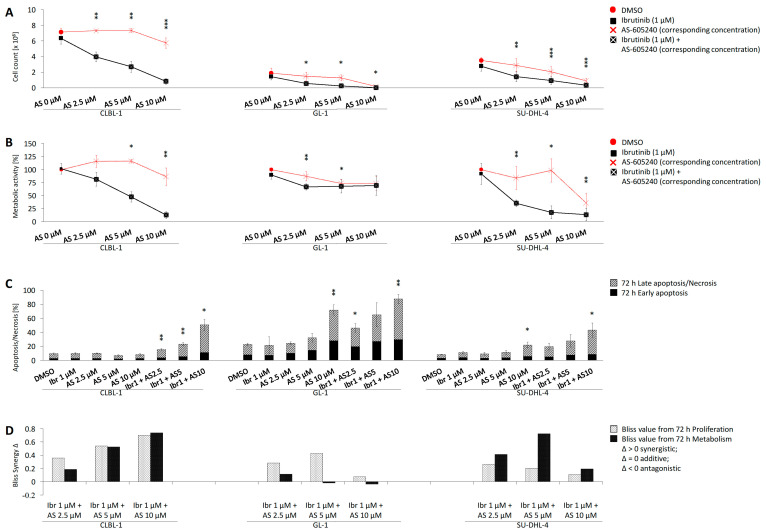
CLBL-1, GL-1, and SU-DHL-4 were incubated with ibrutinib (1 μM) or/and AS-605240 (2.5, 5, and 10 μM) for 72 h. The figures display the average value (±SD) of three independent experiments. The significance of the combined inhibitors compared to the control was calculated as a *p* value using Student’s *t*-Test, shown in (**A**–**C**). When * *p* < 0.05, then **; *p* < 0.01, ***; *p* < 0.001. The significance of the combined inhibitors compared to the single treatment was calculated as a *p* (vs. Sin) value using Student’s *t*-test, shown in Supplementary Table S4C. (**A**) The number of viable cells. (**B**) The metabolic activity (indicated as the percentage compared to the DMSO-treated cells). (**C**) The rate of early apoptosis (annexin V+/PI-) and late apoptosis/necrosis (annexin V+/PI+). (**D**) The synergy of ibrutinib and AS-605240 was evaluated through the Bliss independence model. If Δ > 0, the two inhibitors work synergistically; if Δ = 0, they work additively; if Δ < 0, they work antagonistically.

The proliferation inhibition of CLBL-1 and the reference cell lines GL-1 and SU-DHL-4 was significantly (*p* < 0.05, two-tailed *t*-test) increased compared to DMSO when exposed to the inhibitor combinations (Ibr + AS2.5, Ibr + AS5, Ibr + AS10) for 72 h (Figure 2A, Supplementary Table S4A). Compared to single treatments with ibrutinib (1 μM) or AS-605240 (2.5, 5, 10 μM), combined inhibition (Ibr + AS2.5, Ibr + AS5, Ibr + AS10) significantly inhibited CLBL-1 proliferation. In GL-1, an enhanced anti-proliferative effect was induced by combined inhibitors (Ibr + AS5, Ibr + AS10), as well as in SU-DHL-4 by the combined inhibitor (Ibr + AS10).

Compared to the DMSO control, the metabolic activity inhibitory effect observed in CLBL-1 and reference cell lines GL-1 and SU-DHL-4 was significantly (*p* < 0.05, two-tailed *t*-test) enhanced when exposed to the corresponding combined inhibitors (Ibr + AS2.5, Ibr + AS5, Ibr + AS10) (Figure 2B, Supplementary Table S4B). Compared to single treatment with ibrutinib (1 μM) or AS-605240 (2.5, 5, 10 μM), the combined inhibitor application (Ibr + AS5, Ibr + AS10) significantly inhibited CLBL-1 metabolic activity. In GL-1, enhanced inhibition of the metabolic activity was induced by the combined inhibitors (Ibr + AS2.5), as well as in SU-DHL-4 by the inhibitor combination (Ibr + AS5).

### 2.4. Combined Application of Ibrutinib and AS-605240 Induces Apoptosis and Necrosis of CLBL-1

Compared to the DMSO control or treatment with only ibrutinib (1 μM) or AS-605240 (2.5, 5, 10 μM), the combined inhibitions (Ibr + AS2.5, Ibr + AS5, Ibr + AS10) significantly (*p* < 0.05, two-tailed *t*-test) induced apoptosis/necrosis in CLBL-1. The apoptosis/necrosis inductions of CLBL-1 can be maximally increased to 50.7% by the combined inhibitors (Ibr + AS10), compared to 9.5% by DMSO, 9.8% by 1 μM ibrutinib, and 8.1% by 10 μM AS-605240. Compared to the control, the apoptosis/necrosis induction in GL-1 was significantly increased by the inhibitor combinations (Ibr + AS2.5 and Ibr + AS10), as well as in SU-DHL-4 by combined inhibitors (Ibr + AS10) (Figure 2C, Supplementary Table S4C).

Furthermore, the combined inhibitors caused more CLBL-1 cells to undergo apoptotic and necrotic processes, compared to the cells in the control group. Consequently, cellular fragmentation, membrane blebs, condensation of chromatin, breakdown of plasma membrane and nucleus, leakage of contents, and apoptotic bodies were detected by Pappenheim staining (Supplementary Figure S1).

### 2.5. Bliss Analysis Reveals Synergistic Effects of Ibrutinib and AS-605240

The bliss values calculated based on the proliferation data of CLBL-1, GL-1, and SU-DHL-4 showed that the difference (Δ) between observed and expected values ranged between 0.07 and 0.70, in different combined ibrutinib and AS-605240 groups (Figure 2D, Supplementary Table S4D). Positive bliss values indicated that ibrutinib and AS-605240 had a synergistic effect regarding the proliferation of CLBL-1, GL-1, and SU-DHL-4. Based on the metabolic activity data, the bliss values of CLBL-1 and SU-DHL-4 ranged between 0.19 and 0.74, indicating that ibrutinib and AS-605240 had a synergistic effect on the anti-metabolic activity of CLBL-1 and SU-DHL-4. However, the bliss values of GL-1 were partly below than 0 (Ibr + AS5, Ibr + AS10), indicating that ibrutinib and AS-605240 have an antagonistic effect on the anti-metabolic activity of GL-1.

### 2.6. Single or Combined Ibrutinib and AS-605240 Reduce the Ratio of Phosphorylated and Total Forms of Target Proteins of CLBL-1

**Figure 3:**
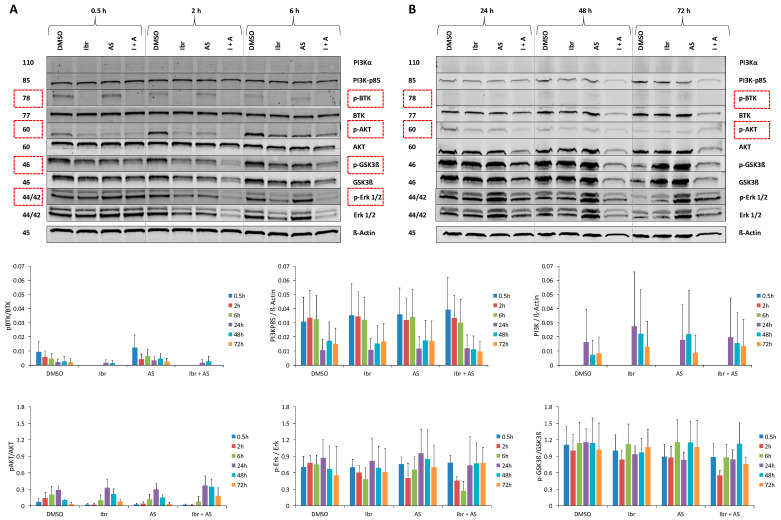
Western blot analysis of CLBL-1 treated with ibrutinib, AS-605240, and their combination for different time periods. Total and phosphorylated forms of key proteins were detected. β-Actin was used as the loading control. The figures display the average value (±SD) of three independent experiments. Pictures are given as examples. (**A**) CLBL-1 cells were exposed to the indicated treatments for 0.5, 2, and 6 h. All bands were on the same membrane. (**B**) CLBL-1 cells were exposed to the indicated treatments for 24, 48, and 72 h. All bands were on the same membrane. The protein in the red box was the group showing combined inhibitory effect.

Western blot analyses revealed that the expression of p-BTK, p-AKT, p-GSK3β, and p-ERK1/2 in CLBL-1 was reduced by ibrutinib and combined inhibitors (Ibr + AS5) after exposure for different time periods (Figure 3A,B, Supplementary Table S5C). At specific time points, ibrutinib inhibited the ratio of the phosphorylated and total form of BTK (0.5–6 and 72 h), AKT (0.5–6 h), GSK3β (0.5, 2, and 24 h), and ERK (2 and 6 h). AS-605240 reduced the ratio of the phosphorylated and total forms of AKT (0.5–6 h), GSK3β (0.5, 2, and 24 h), and ERK (2 h) at specific time points. AS-605240 did not cause changes in the levels of PI3K-p85.

Compared to the DMSO, the combination of ibrutinib and AS-605240 reduced the ratio of phosphorylated and total forms of BTK (0.5–6 and 72 h), with an intensity similar to that of the treatment with ibrutinib alone. The combination also reduced p-ERK/ERK (2 and 6 h), p-GSK3β/GSK3β (0.5–24 and 72 h), and p-AKT/AKT (0.5–6 h) ratios at specific time points.

### 2.7. RNA-Seq Analysis of Canine B- and T-lymphoid Cell Lines and Primary Lymphoma Samples Reveals Potentially High-Impact Variants Predominantly in PI3K Transcripts

**Figure 4:**
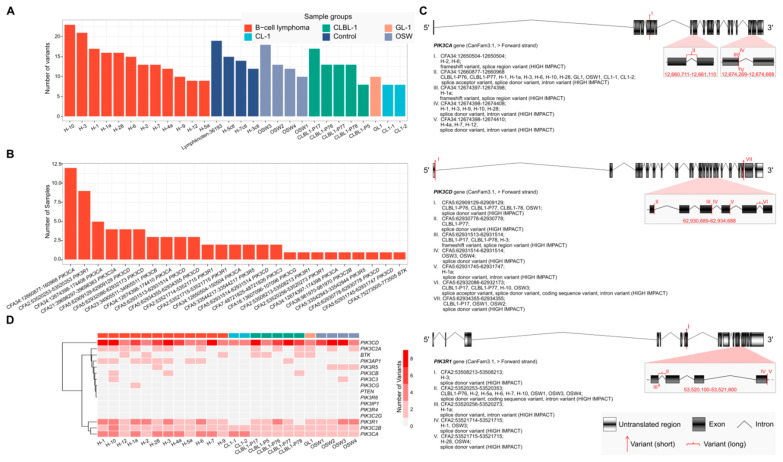
Short variant analysis of canine B- and T-lymphoid cell lines and primary lymphoma samples reveals variants in *PI3K* and *BTK*. (**A**) Number of variants detected in each sample. (**B**) Number of samples with variants of *PI3K* and *BTK* detected in lymphoid cell lines and/or lymphoma samples but not in non-neoplastic control samples. (**C**) Transcript structures of *PIK3CA*, *PIK3CD*, and *PIK3R1* with different variants were visualized. (**D**) Heat map displaying the number of variants detected in each of 16 transcripts in 24 neoplastic samples of 5 different sample groups.

Examination of RNA-seq data from 28 samples comprising two canine B lymphoid cell lines, two T lymphoid cell lines, primary B-cell lymphomas, and non-neoplastic controls revealed frequent potentially loss-of-function variants among some of the genes encoding the primary targets of AS-605240 and ibrutinib and their regulators: *BTK, PI3K*, and *PTEN*. Each sample had an average of 13 variants with a standard deviation of 4 variants (Figure 4A); these variants affected an average of 6 genes with a standard deviation of 1 gene. While 29 of the 54 variants were present in one or more lymphoid cell lines or primary B-cell lymphomas as well as in one or more non-neoplastic control samples, 25 were displayed by lymphoid cell lines and/or lymphoma samples but not by any non-neoplastic control sample (Figure 4B). Most (76%) of these 25 variants were observed in three or fewer samples. However, two INDELS (CFA2, NC_006584.3:g.53520253_53520353del and CFA34, NC_006616.3:g.12660877-12660968del; CanFam3.1) were identified in 9 and 12 samples representing 3 and 5 different sample groups, respectively. Specifically, the 25 variants affected 10 of the 16 transcripts of interest (*BTK*, *PIK3CD*, *PIK3R1*, *PIK3CA*, *PIK3R5*, *PIK3C2B*, *PIK3C2A*, *PIK3CG*, *PIK3CB*, *and PIK3C3*) at different locations (Figure 4C; Supplementary Table S6B, Supplementary Figure S3). Most (15, 60%) of these variants were predicted to be donor splice site variants. Notably, *PIK3CD*, *PIK3R1*, and *PIK3CA* exhibited variants in a relatively large number of samples (11, 13, and 17, respectively), representing most of the neoplastic sample groups (3, 3, and 5, respectively; Figure 4D). No variants of *PTEN* were found in the examined samples.

## 3. Discussion

DLBCL is often characterized by increased tyrosine phosphorylation activities compared to normal resting B cells. Accordingly, the application of different TKIs has shown promising pre-clinical and clinical results in different hematologic neoplasias, including DLBCL [20]. Among them, BTK and PI3K inhibitors have exhibited encouraging clinical results. On the one hand, the BTK inhibitor ibrutinib has significant value for the treatment of human DLBCL patients, particularly with ABC-DLBCL subtype [7], and has been shown to exert dose-dependent proliferation inhibition and IgE-dependent histamine release suppression in a pre-clinical model of canine mast cell tumor [21]. Further, although ibrutinib has not yet been approved for veterinary medicine, a dose-escalation study has been performed in dogs [22]. In addition, in 2013, Honigberg et al. reported the use of ibrutinib in canine B-cell lymphoma, a spontaneous animal model for B-cell malignancies. Of a total of eight treated dogs, three had partial responses, including “follicular large cell” morphology in two and “diffuse immunoblastic” in one [8]. On the other hand, PI3K targeting by selective inhibitors such as idelalisib has been introduced in the human clinic and was pre-clinically evaluated in dogs [23]. While idelalisib was found to induce severe lethal side effects leading to a hold of several idelalisib mono and combination trials, the general strategy to target PI3K remains a promising approach [24,25]. The herein used selective PI3Kγ inhibitor AS-605240 has been demonstrated to have anti-leukemic activity and strong synergy with glucocorticoids both in vitro and in a NOD/SCID xenograft mouse model of human T-ALL [26]. Nevertheless, we are not aware of any report on the application of AS-605240 on canine cells, making this the first one. The herein used CLBL-1 cell line represents a well-characterized in vitro model for the canine activated B-cell-like (ABC) DLBCL subtype [27]. In view of the achievements of ibrutinib and AS-605240, the combination of these two inhibitors has potential evaluation and application value in in vitro and in vivo canine DLBCL models. Similar to ibrutinib, AS-605240 has not yet been approved for veterinary use. In contrast to ibrutinib, no dose-escalation study has been conducted for AS-605240 in dogs. Therefore, a single-drug dose-escalation study for AS-605240 is a prerequisite for proceeding with an actual combined drug regimen in canine lymphoma patients.

Evaluation of the combined drug application showed significant synergistic effects. As a single agent, AS-605240 only exhibited an anti-proliferation effect in CLBL-1 at the highest concentration assayed (10 μM), reducing cell proliferation by 14% compared to the DMSO control. Similarly, treatment with ibrutinib alone at a concentration of 1 μM had a small effect, reducing cell proliferation by 5%. In contrast, the combined application of ibrutinib (1 μM) and AS-605240 reduced cell proliferation by at least 40%. Moreover, combining ibrutinib (1 μM) with AS-605240 at a concentration of 10 μM resulted in the survival of only 11% of CLBL-1 cells. Thus, combining these two inhibitors significantly enhances the anti-proliferative effects compared to either of the single treatments. The synergistic effect between ibrutinib and AS-605240 on cell proliferation and metabolic activity was further confirmed using the Bliss independence model. This is consistent with the study of Griner et al., who described a high-throughput screening platform that could rapidly and systematically identify synergistic, additive, and antagonistic drug combinations and found that the combination of ibrutinib with various PI3K pathway inhibitors, such as MK-2206, CAL-101, BKM-120, BEZ-235, GDC-0941, GDC-0980, everolimus, and PRT-060318, had either synergistic or additive effects on the inhibition of human ABC-DLBCL line TMD8 [28]. In order to compare the observed responses in CLBL-1, we analyzed two reference cell lines: the human GCB-DLBCL cell line SU-DHL-4 and the canine B-cell leukemia cell line GL-1. Both cell lines recapitulated the effects observed in the canine B-cell lymphoma cell line, showing synergistic proliferation inhibition as a response to combined ibrutinib and AS-605240 exposure. Single application of ibrutinib caused a distinct effect, inhibiting the ABC-DLBCL subtype cell line CLBL-1 and, although less efficiently, the GCB-DLBCL subtype SU-DHL-4 cells [7,29]. According to the previously described data on PI3K inhibition in combination with ibrutinib and our data, the addition of AS-605240 bears solid potential to enhance ibrutinib-based protocols in both subtypes. This study lays the foundation for prospective clinical trials in dogs, contingent upon a positive drug safety evaluation of ibrutinib and AS-605240.

Western blot analyses were performed to evaluate the effect of the applied inhibitors on selected key downstream targets of the canine PI3K pathway [30,31,32,33,34]. The results presented herein showed that ibrutinib reduced the ratio of phosphorylated and total forms of BTK, AKT, and ERK in CLBL-1 at specific time points. This is consistent with previous findings in humans [30]. AS-605240 targets the catalytic subunit of PI3K (class I) and is more selective for PI3Kγ than for PI3Kα/β/δ [35]. In this study, AS-605240 did not induce a significant decrease of PI3K-p85, which belongs to the regulatory subunit of PI3K (class I) [35]. Nevertheless, the combined application of ibrutinib and AS-605240 reduced the ratio of phosphorylated and total forms of BTK, AKT, GSK3β, and ERK at specific time points. These proteins are involved in the development, proliferation, survival, and motility of lymphoma cells. We propose an approach based on the inhibition of two different effectors on the BCR pathway, BTK and PI3Kγ that will affect most of the terminal effectors of the pathway, interfering with the function of Ras/MEK/ERK, AKT/mTOR, FOXO, NFkB, MYC, and ELK. The induced biological effects could be of considerable value for DLBCL, as PI3K is essential for the activity of BTK and BTK links BCR activity to NF-kB [36].

The observed changes in protein level are directly dependent on the ability of the inhibitors to bind the respective isoform. Hence, structural aberrations within the target transcripts are of major significance and the efficiency of TKIs can be substantially influenced by the presence of genetic variants affecting the transcripts that encode the target proteins [17,18,19]. Different in vitro and xenograft models are normally used while evaluating novel therapeutic approaches before clinical testing. Therefore, genetic variants dominantly present in the initial neoplasias or accumulated by clonal selection of the cells, may influence the information gained from these modes. In addition to variants that were common among non-neoplastic control samples, 25 potentially high-impact variants, predicted by the Ensembl Variant Effect Predictor, were found to affect *PI3K* and *BTK* only in lymphoid cell lines and primary B-cell lymphomas. Overall, 15 (60%) of the 25 variants were predicted to alter splicing. Since these variants are found both among cell lines and spontaneously arising lymphoma samples, it is likely that the above-described effects for ibrutinib and AS-605240 on the CLBL-1 cell line will have high transfer potential to the lymphoma patients showing similar aberrations. Prominently, *PIK3CA* is the only member of the PI3K pathway that exhibits frequent activating mutations in various tumor types [19]. In this study, we observed three variants (CF34, NC_006616.3:g.12674397_12674398; NC_006616.3:g.12674398_12674408del; NC_006616.3:g.12674398-12674410del; CanFam3.1) on one particular exon of *PIK3CA* that would potentially affect the protein catalytic domain of phosphatidylinositol 3-/4-kinase in dogs. Within the PI3K/AKT/mTOR pathway, the activity of PTEN leads to hyperactivation of this pathway, inducing drug resistance. Scuoppo et al. described that PTEN suppression and PI3K activation induced resistance to dasatinib, a multi-kinase inhibitor, in a model with multiple genetically characterized DLBCL cell lines in the presence of *PTEN* mutations [11]. In their study, an mTOR inhibitor showed synergistic effects when combined with dasatinib, and it was more efficient than selective PI3K or AKT inhibitors in deregulating PI3K activity. However, in the samples we examined, no variants of *PTEN* were found. Therefore, the resistance of the ABC-DLBCL cell line used in this experiment to AS-605240 may be due to excessive activation of PI3K mutations.

The concentrations used in the present study for the combination assays have only been studied in vitro before [37,38,39]. Although there is no report indicating whether or not these concentrations are feasible in vivo, the European Medicines Agency has concluded from a dose-escalating study in dogs that the maximum tolerated oral dose of ibrutinib is 200 mg/kg (around 454 μmol/kg) [22]. Even though a direct comparison of drug dosages in vitro and in vivo should be treated with caution, the concentrations employed here were within this range. As mentioned before, AS-605240 has never been used in dog patients. Therefore, a sine qua non for a drug combination approach in dogs is a dose-escalation study of AS-605240 for the dosages that have already been applied in other mammalian species. A subsequent clinical study combining ibrutinib and AS-605240 in dogs would be of significant value due to the high incidence of DLBCL and the high relapse rates in this species. A subsequent clinical study combining ibrutinib and AS-605240 in dogs would be of significant value due to the high incidence of DLBCL and the high relapse rates in this species. A precondition for an advanced in vivo evaluation is the basic pharmacokinetic evaluation of the drug combination in terms of clearance, tissue and fluid accumulation, and metabolite characterization. In this, the data available due to the single-drug approvals of BTK inhibitors such as ibrutinib or acalabrutinib for lymphomas in humans as well as the data on approved PI3K inhibitors will allow realizing this in a considerably shortened time frame. As mentioned before, both single-drug escalation and combined-drug escalation studies are required to increase safety for canine patients. Since in dogs this can be realized in a relatively short time, a clinical trial complying with patient safety for the drug combination appears feasible in the foreseeable future.

## 4. Materials and Methods

### 4.1. Cell Lines and Culture Condition

CLBL-1 and GL-1 were kindly provided by the University of Veterinary Medicine, Vienna, Austria. The human cell line, SU-DHL-4, was purchased from the institute (Leibniz Institute DSMZ—German Collection of Microorganisms and Cell Cultures GmbH, Braunschweig, Germany).

The CLBL-1 cell line was isolated from a male Bernese mountain dog with confirmed stage IV diffuse large cell lymphoma [27]. GL-1 was a B-cell leukemia cell line, derived from a German Shepherd [40]. The DLBCL cell line SU-DHL-4 was extracted and cultured from a 38-year-old male patient with diffuse histiocytic lymphoma [41]. CLBL-1, GL-1, and SU-DHL-4 were cultured in RPMI-1640 medium, supplemented with 10% heat-inactivated fetal bovine serum and 1% penicillin–streptomycin (10,000 U/mL penicillin, 10 mg/mL streptomycin, Biochrom GmbH, Berlin, Germany). Cells were incubated at 37 °C in a humidified atmosphere of 5% CO_2_, and the medium was changed twice a week.

### 4.2. Tyrosine Kinase Inhibitors

Ibrutinib (Ibr) and AS-605240 (AS) were purchased from Selleck Chemicals (Absource Diagnostics GmbH, München, Germany). According to the manufacturer’s information, the substances were dissolved in dimethyl sulfoxide (DMSO, Sigma-Aldrich Chemie GmbH, Steinheim, Germany) to form a 10 mM stock solution and stored at −80 °C.

### 4.3. Single Drug Application Experiments

For all drug application experiments, cells were seeded at a density of 0.33 × 10^6^ cells/mL. For the single-treatment setup (proliferation, metabolic activity, and apoptosis/necrosis assay) of CLBL-1, the cells were separately incubated with serial concentrations (0.001, 0.01, 0.1, 0.5, 1, 2.5, 5, 10 μM) of ibrutinib or AS-605240 for 24, 48, and 72 h. For the single-treatment setup of the comparison cell lines GL-1 and SU-DHL-4, the cells were separately incubated with selective serial concentrations (0.1, 2.5, 5 μM) of ibrutinib or AS-605240 for 24, 48, and 72 h.

### 4.4. Combined Drug Application Experiments

For the combined application experiments (proliferation, metabolic activity, and apoptosis/necrosis assay) of CLBL-1, GL-1, and SU-DHL-4, cells were separately incubated with 1 μM ibrutinib and various concentrations (2.5, 5, 10 μM) of AS-605240 based on the single drug application of CLBL-1 for 72 h. For the morphology assay, CLBL-1 was separately incubated with ibrutinib (1 μM), AS-605240 (2.5, 5, 10 μM), and their corresponding combinations for 24, 48, and 72 h. For the Western blot analyses, the CLBL-1 was separately incubated with 1 μM ibrutinib, 5 μM AS-605240, and their combination for 0.5, 2, 6, 24, 48, and 72 h.

Cells in the control group were cultured in their medium with the same concentrations of DMSO used for the drug-treated cells, i.e., 0.1% (*v*/*v*) for the single application and 0.2% (*v*/*v*) for combined application.

### 4.5. Cell Proliferation and Metabolic Activity

Cell proliferation was evaluated by trypan blue dye exclusion. Briefly, 0.5 × 10^6^ cells per well were seeded in duplicate in the 24-well plate and exposed to inhibitor and vehicle. Cells were harvested at each time point and washed with PBS. The number of viable cells was determined by counting with a hemocytometer and trypan blue staining (Marienfeld Superior, Lauda-Königshofen, Germany).

Metabolic activity was assessed with the premix WST-1 cell proliferation assay system. For this, 5 × 10^4^ cells per well were seeded in triplicate in the 96-well plate and exposed to inhibitor and vehicle. When the cells reached each time point, 15 µL of pre-warmed WST-1 reagent was added into 150 µL cell suspension and medium control and incubated at 37 °C for 2 h. Finally, the change in absorbance was detected by PromegaGloMax^®^-Multi Microplate Multimode Reader (Promega, Madison, WI, USA). When analyzing the data, the average absorbance value of the reference wavelength was subtracted from that of the sample wavelength, which correlated with the number of viable cells.

### 4.6. Apoptosis and Necrosis Measurement

Apoptosis and necrosis were assessed by flow cytometry, in which cells were stained with annexin V FITC (BD Biosciences, Heidelberg, Germany) and propidium iodide (PI) solution (Sigma Aldrich, St. Louis, MO, USA) [42]. Cells treated with single or combined inhibitors for different time points were harvested, centrifuged (180× *g*, 10 min, 4 °C) and washed with PBS twice. After washing steps, the cell pellet was resuspended in 100 µL annexin binding buffer (1×) (Becton, Dickinson and Company, Heidelberg, Germany), then incubated with 5 µL of annexin V FITC for 15 min at room temperature in the dark. The cell suspension was adjusted to a final volume of 500 µL with annexin binding buffer, the cells were stained with PI (20 µg/mL) immediately before measurement. Unstained and mono-stained cells were regarded as the control in each experiment. The measurement was carried out on BD FACS Verse™ flow cytometer, and data were analyzed using BD FACSuite™ software (Becton, Dickinson and Company, Heidelberg, Germany).

### 4.7. Detection of Synergistic Drug Combinations

The synergy between ibrutinib and AS-605240 was assessed using the Bliss independence model. The synergistic effects of a drug combination are determined by the difference (Δ) between the observed (O) and the expected (E) inhibition of the combined treatment. E is calculated as follows: E = A + B − A⋅B, where A and B are the relative inhibition of single-inhibitor A and B. In this case, Δ > 0 indicates a synergistic effect and Δ < 0, an antagonistic effect [43,44]. Bliss values for ibrutinib (1 µM) combined with various concentrations (2.5, 5, 10 μM) of AS-605240 were calculated based on our findings for the proliferation and metabolic activity of CLBL-1, GL-1, and SU-DHL-4.

### 4.8. Examination of Cell Morphology

Pappenheim staining was used to examine the morphology of CLBL-1 after drug exposition. After harvesting the cells at every time point, each sample was fixed on three glass slides by the Shandon Cytospin 3 centrifuge (Shandon, Frankfurt/Main, Germany), which were adjusted to the concentration of 5 × 10^4^ in 200 µL PBS per slide. After air-drying the slides, they were stained with May–Grünwald solution (Merck, Darmstadt, Germany) for 6 min, washed with buffer (pH = 7.2), stained with Giemsa solution (1:10, Merck, Darmstadt, Germany) for 20 min, and washed with buffer again. When slides were air-dried, the morphology of cells was examined and visualized with Evos XL Core Imaging System (Life Technologies, Darmstadt, Germany), magnified 100 times.

### 4.9. Protein Expression Analyses Using Western Blot

The expressions of targeted proteins, PI3K, phosphorylated and total forms of BTK, AKT, GSK-3β, and ERK1/2 were comparatively evaluated by a Western blot. β-Actin was used as the loading control. Before the Western blot experiments, the cross reactivity of all primary antibodies was verified on the human precursor B acute lymphoblastic leukemia cell line SEM, human Burkitt lymphoma cell lines Raji and Ramos, as well as canine CLBL-1 cell line (Supplementary Figure S2).

CLBL-1 cells were exposed to 1 µM ibrutinib, 5 µM AS-605240, and their combination for 0.5, 2, 6, 24, 48, and 72 h. Cells were lysed with 80–100 µL 1x RIPA buffer (10x RIPA, 100x Protease/Phosphatase Inhibitor Cocktail (Cell Signaling Technology, Frankfurt, Germany)) and ultrasound (Bandelin, Berlin, Germany). The protein concentrations were determined by Bradford Assay (Bio-Rad, München, Germany). Briefly, 30 µg of protein per sample was loaded into the Criterion^TM^ TGX^TM^ Precast Gel (Bio-Rad, München, Germany) and separated by electrophoresis. Proteins were transferred onto a PVDF membrane (Bio-Rad, München, Germany) by a Trans-Blot Turbo Transfer System (Bio-Rad, München, Germany) and blocked in 1:3 diluted Odyssey blocking buffer (LI-COR GmbH, Nebraska, USA) at room temperature for 1 h. After the blocking step, membranes were incubated with the primary antibody solutions (1:5 Blocking Buffer/PBST) at 4 °C overnight (Supplementary Table S5A). Membranes were washed and incubated with fluorescent labeled secondary antibody for one hour (Supplementary Table S5B). After washing, the proteins were detected using a LI-COR Odyssey Imager and Image Studio Lite software (LI-COR GmbH, Lincoln, NE, USA).

### 4.10. Variant Calling on RNA-Seq Data

Raw sequencing data from 28 samples in 6 sample groups, i.e., the canine B-lymphoid cell lines CLBL-1 (*n* = 5) and GL-1 (*n* = 1), the canine T-lymphoid cell lines CL-1 (*n* = 2) [45] and OSW (*n* = 4) [46], canine primary B-cell lymphomas (*n* = 12), and non-neoplastic controls (*n* = 4), were previously generated by us [47] and deposited at the NCBI GEO database (GSE112474).

Raw sequencing data had already been pre-processed [47]. Starting from the BAM-formatted files, variants were called according to the Genome Analysis Toolkit (GATK) Best Practices workflow for calling single-nucleotide polymorphisms (SNPs) and insert/deletions (INDELs) on RNA-seq data (https://gatkforums.broadinstitute.org/gatk/discussion/3891/calling-variants-in-rnaseq, last accessed on 8 October 2019). GATK version 3.4–46 [48] was used throughout the analysis. Firstly, Picard Toolkit (2.15.0, Broad Institute, GitHub Repository) was used for adding read group information, sorting, marking duplicates, and indexing. Next, GATK SplitNCigarReads was applied to split reads into exon segments and trim any sequences overhanging into the intronic regions. Then, GATK base-quality score recalibration (BQSR) was called to detect systematic errors caused by the sequencer and subsequently adjust the base quality score. After recalibration, SNPs and INDELS were called jointly with the GATK HaplotypeCaller. Lastly, variants were filtered based on their depth of coverage (DP), strand bias (SB), variant confidence (QUAL), QualByDepth (QD), FisherStrand (FS), RMSMappingQuality (MQ), MappingQualityRankSumTest (MQRankSum), ReadPosRankSumTest (ReadPosRankSum), and StrandOddsRatio (SOR). Specifically, GATK VariantFiltration was applied with the parameters “--filterExpression ‘DP < 5 || SB > −0.1 || QUAL < 50.0 || QD < 2.0 || FS > 60.0 || MQ < 40.0 || MQRankSum < −12.5 || ReadPosRankSum< −8.0 || DP > 500’” and “--filterExpression ‘QD < 2.0 || FS > 200.0 || ReadPosRankSum< −20.0 || SOR > 10.0’” to filter out SNPs and INDELS, respectively, that are likely to be artefacts.

Filtered variants within the body of any of the genes of interest (Supplementary Table S6A) were annotated with the Ensembl Variant Effect Predictor (VEP, version 98.2) [49]. All variants classified by VEP as “IMPACT = HIGH” were used to conduct a comparative burden analysis of the samples. Variants classified as “IMPACT = HIGH” include classic loss-of-function variants (Sequence Ontology terms: “transcript_ablation”, “transcript_amplification”, “frameshift_variant”, “splice_acceptor_variant”, “splice_donor_variant”, “start_lost”’, “stop_gained”, and/or “stop_lost”, http://www.sequenceontology.org/, last accessed on 31 July 2020). Variants classified as “IMPACT = MODERATE”, “IMPACT = LOW”, or “IMPACT = MODIFIER” (including missense, synonymous, in-frame insertions and deletions, etc.) were excluded from the analysis.

### 4.11. Statistical Analysis and CLBL-1 IC50 Identification

All experiments were independently repeated at least three times. Results of proliferation, metabolic activity, and apoptosis/necrosis were described using mean value and standard deviation. Significance between the inhibitor exposure and control group was calculated using the two-tailed Student’s *t*-test [43]. *p*-Values (*p*) < 0.05 were considered to be significant. When *p* < 0.05, then *; *p* < 0.01, **; *p* < 0.001, ***. IC50 of CLBL-1 was calculated through Graph Pad Prism Version 8.0.2, based on the number of the living cells for the 72 h group. The concentrations and cell numbers were log-transformed and normalized. Afterward, nonlinear regression (dose-response inhibition, log vs. normalized response–variable slope) was used to determine the IC50 values.

## 5. Conclusions

Ibrutinib and AS-605240 act synergistically to inhibit cell proliferation, metabolic activity, and to induce apoptosis on CLBL-1, also to affect GL-1 and SU-DHL-4 as reference cell lines. Comparative analysis among canine B- and T-lymphoid cell lines and primary B-cell lymphoma samples, reveals potentially high-impact variants in *PI3K* and *BTK*. These data emphasize the importance of PI3K as a therapeutic target in canine DLBCL and additionally confirm that the combination with a BTK inhibition acts synergistically disrupting the BCR pathway. The reported combination shows a promising therapeutic approach in canine DLBCL that can be used as a valuable in vivo model for human DLBCL. The present study supports the combined application of ibrutinib and AS-605240 in vivo in canine DLBCL as the available therapies fail to achieve cure or long-lasting remissions.

## Data Availability

The data supporting the reported results can be found in the website mentioned in the article.

## Supplementary Materials

The following are available online at https://www.mdpi.com/article/10.3390/ijms222312673/s1.
