# Supplementary material for: BTK and PI3K Inhibitors Reveal Synergistic Inhibitory Anti-Tumoral Effects in Canine Diffuse Large B-Cell Lymphoma Cells
Source: Int J Mol Sci. 2021 Nov 24;22(23):12673. doi: 10.3390/ijms222312673 (PMC8658042; doi:10.3390/ijms222312673)

## Slide 1
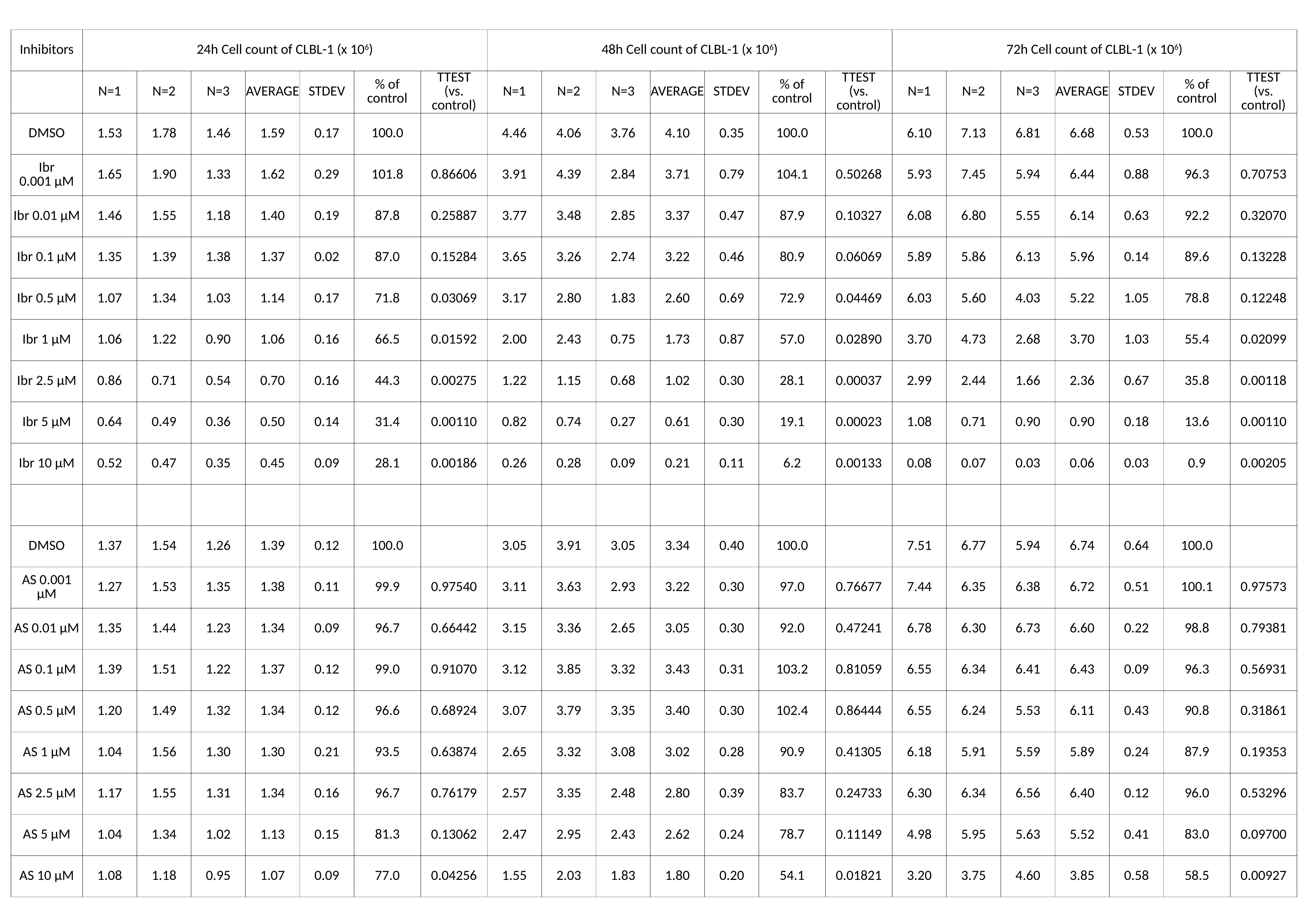

| Inhibitors | 24h Cell count of CLBL-1 (x 106) | | | | | | | 48h Cell count of CLBL-1 (x 106) | | | | | | | 72h Cell count of CLBL-1 (x 106) | | | | | | |
| --- | --- | --- | --- | --- | --- | --- | --- | --- | --- | --- | --- | --- | --- | --- | --- | --- | --- | --- | --- | --- | --- |
| | N=1 | N=2 | N=3 | AVERAGE | STDEV | % of control | TTEST (vs. control) | N=1 | N=2 | N=3 | AVERAGE | STDEV | % of control | TTEST (vs. control) | N=1 | N=2 | N=3 | AVERAGE | STDEV | % of control | TTEST (vs. control) |
| DMSO | 1.53 | 1.78 | 1.46 | 1.59 | 0.17 | 100.0 | | 4.46 | 4.06 | 3.76 | 4.10 | 0.35 | 100.0 | | 6.10 | 7.13 | 6.81 | 6.68 | 0.53 | 100.0 | |
| Ibr 0.001 µM | 1.65 | 1.90 | 1.33 | 1.62 | 0.29 | 101.8 | 0.86606 | 3.91 | 4.39 | 2.84 | 3.71 | 0.79 | 104.1 | 0.50268 | 5.93 | 7.45 | 5.94 | 6.44 | 0.88 | 96.3 | 0.70753 |
| Ibr 0.01 µM | 1.46 | 1.55 | 1.18 | 1.40 | 0.19 | 87.8 | 0.25887 | 3.77 | 3.48 | 2.85 | 3.37 | 0.47 | 87.9 | 0.10327 | 6.08 | 6.80 | 5.55 | 6.14 | 0.63 | 92.2 | 0.32070 |
| Ibr 0.1 µM | 1.35 | 1.39 | 1.38 | 1.37 | 0.02 | 87.0 | 0.15284 | 3.65 | 3.26 | 2.74 | 3.22 | 0.46 | 80.9 | 0.06069 | 5.89 | 5.86 | 6.13 | 5.96 | 0.14 | 89.6 | 0.13228 |
| Ibr 0.5 µM | 1.07 | 1.34 | 1.03 | 1.14 | 0.17 | 71.8 | 0.03069 | 3.17 | 2.80 | 1.83 | 2.60 | 0.69 | 72.9 | 0.04469 | 6.03 | 5.60 | 4.03 | 5.22 | 1.05 | 78.8 | 0.12248 |
| Ibr 1 µM | 1.06 | 1.22 | 0.90 | 1.06 | 0.16 | 66.5 | 0.01592 | 2.00 | 2.43 | 0.75 | 1.73 | 0.87 | 57.0 | 0.02890 | 3.70 | 4.73 | 2.68 | 3.70 | 1.03 | 55.4 | 0.02099 |
| Ibr 2.5 µM | 0.86 | 0.71 | 0.54 | 0.70 | 0.16 | 44.3 | 0.00275 | 1.22 | 1.15 | 0.68 | 1.02 | 0.30 | 28.1 | 0.00037 | 2.99 | 2.44 | 1.66 | 2.36 | 0.67 | 35.8 | 0.00118 |
| Ibr 5 µM | 0.64 | 0.49 | 0.36 | 0.50 | 0.14 | 31.4 | 0.00110 | 0.82 | 0.74 | 0.27 | 0.61 | 0.30 | 19.1 | 0.00023 | 1.08 | 0.71 | 0.90 | 0.90 | 0.18 | 13.6 | 0.00110 |
| Ibr 10 µM | 0.52 | 0.47 | 0.35 | 0.45 | 0.09 | 28.1 | 0.00186 | 0.26 | 0.28 | 0.09 | 0.21 | 0.11 | 6.2 | 0.00133 | 0.08 | 0.07 | 0.03 | 0.06 | 0.03 | 0.9 | 0.00205 |
| | | | | | | | | | | | | | | | | | | | | | |
| DMSO | 1.37 | 1.54 | 1.26 | 1.39 | 0.12 | 100.0 | | 3.05 | 3.91 | 3.05 | 3.34 | 0.40 | 100.0 | | 7.51 | 6.77 | 5.94 | 6.74 | 0.64 | 100.0 | |
| AS 0.001 µM | 1.27 | 1.53 | 1.35 | 1.38 | 0.11 | 99.9 | 0.97540 | 3.11 | 3.63 | 2.93 | 3.22 | 0.30 | 97.0 | 0.76677 | 7.44 | 6.35 | 6.38 | 6.72 | 0.51 | 100.1 | 0.97573 |
| AS 0.01 µM | 1.35 | 1.44 | 1.23 | 1.34 | 0.09 | 96.7 | 0.66442 | 3.15 | 3.36 | 2.65 | 3.05 | 0.30 | 92.0 | 0.47241 | 6.78 | 6.30 | 6.73 | 6.60 | 0.22 | 98.8 | 0.79381 |
| AS 0.1 µM | 1.39 | 1.51 | 1.22 | 1.37 | 0.12 | 99.0 | 0.91070 | 3.12 | 3.85 | 3.32 | 3.43 | 0.31 | 103.2 | 0.81059 | 6.55 | 6.34 | 6.41 | 6.43 | 0.09 | 96.3 | 0.56931 |
| AS 0.5 µM | 1.20 | 1.49 | 1.32 | 1.34 | 0.12 | 96.6 | 0.68924 | 3.07 | 3.79 | 3.35 | 3.40 | 0.30 | 102.4 | 0.86444 | 6.55 | 6.24 | 5.53 | 6.11 | 0.43 | 90.8 | 0.31861 |
| AS 1 µM | 1.04 | 1.56 | 1.30 | 1.30 | 0.21 | 93.5 | 0.63874 | 2.65 | 3.32 | 3.08 | 3.02 | 0.28 | 90.9 | 0.41305 | 6.18 | 5.91 | 5.59 | 5.89 | 0.24 | 87.9 | 0.19353 |
| AS 2.5 µM | 1.17 | 1.55 | 1.31 | 1.34 | 0.16 | 96.7 | 0.76179 | 2.57 | 3.35 | 2.48 | 2.80 | 0.39 | 83.7 | 0.24733 | 6.30 | 6.34 | 6.56 | 6.40 | 0.12 | 96.0 | 0.53296 |
| AS 5 µM | 1.04 | 1.34 | 1.02 | 1.13 | 0.15 | 81.3 | 0.13062 | 2.47 | 2.95 | 2.43 | 2.62 | 0.24 | 78.7 | 0.11149 | 4.98 | 5.95 | 5.63 | 5.52 | 0.41 | 83.0 | 0.09700 |
| AS 10 µM | 1.08 | 1.18 | 0.95 | 1.07 | 0.09 | 77.0 | 0.04256 | 1.55 | 2.03 | 1.83 | 1.80 | 0.20 | 54.1 | 0.01821 | 3.20 | 3.75 | 4.60 | 3.85 | 0.58 | 58.5 | 0.00927 |

## Slide 2
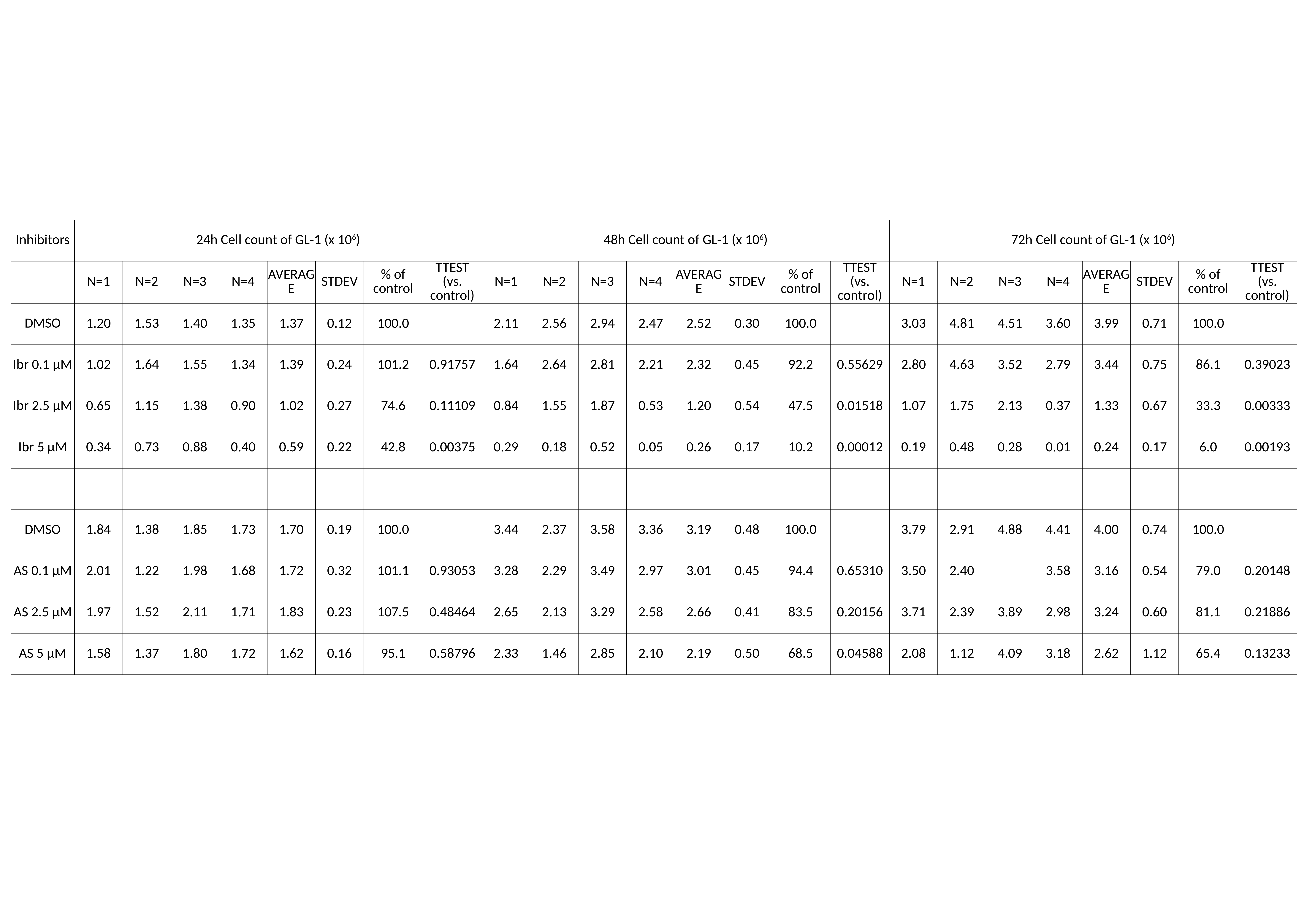

| Inhibitors | 24h Cell count of GL-1 (x 106) | | | | | | | | 48h Cell count of GL-1 (x 106) | | | | | | | | 72h Cell count of GL-1 (x 106) | | | | | | | |
| --- | --- | --- | --- | --- | --- | --- | --- | --- | --- | --- | --- | --- | --- | --- | --- | --- | --- | --- | --- | --- | --- | --- | --- | --- |
| | N=1 | N=2 | N=3 | N=4 | AVERAGE | STDEV | % of control | TTEST (vs. control) | N=1 | N=2 | N=3 | N=4 | AVERAGE | STDEV | % of control | TTEST (vs. control) | N=1 | N=2 | N=3 | N=4 | AVERAGE | STDEV | % of control | TTEST (vs. control) |
| DMSO | 1.20 | 1.53 | 1.40 | 1.35 | 1.37 | 0.12 | 100.0 | | 2.11 | 2.56 | 2.94 | 2.47 | 2.52 | 0.30 | 100.0 | | 3.03 | 4.81 | 4.51 | 3.60 | 3.99 | 0.71 | 100.0 | |
| Ibr 0.1 µM | 1.02 | 1.64 | 1.55 | 1.34 | 1.39 | 0.24 | 101.2 | 0.91757 | 1.64 | 2.64 | 2.81 | 2.21 | 2.32 | 0.45 | 92.2 | 0.55629 | 2.80 | 4.63 | 3.52 | 2.79 | 3.44 | 0.75 | 86.1 | 0.39023 |
| Ibr 2.5 µM | 0.65 | 1.15 | 1.38 | 0.90 | 1.02 | 0.27 | 74.6 | 0.11109 | 0.84 | 1.55 | 1.87 | 0.53 | 1.20 | 0.54 | 47.5 | 0.01518 | 1.07 | 1.75 | 2.13 | 0.37 | 1.33 | 0.67 | 33.3 | 0.00333 |
| Ibr 5 µM | 0.34 | 0.73 | 0.88 | 0.40 | 0.59 | 0.22 | 42.8 | 0.00375 | 0.29 | 0.18 | 0.52 | 0.05 | 0.26 | 0.17 | 10.2 | 0.00012 | 0.19 | 0.48 | 0.28 | 0.01 | 0.24 | 0.17 | 6.0 | 0.00193 |
| | | | | | | | | | | | | | | | | | | | | | | | | |
| DMSO | 1.84 | 1.38 | 1.85 | 1.73 | 1.70 | 0.19 | 100.0 | | 3.44 | 2.37 | 3.58 | 3.36 | 3.19 | 0.48 | 100.0 | | 3.79 | 2.91 | 4.88 | 4.41 | 4.00 | 0.74 | 100.0 | |
| AS 0.1 µM | 2.01 | 1.22 | 1.98 | 1.68 | 1.72 | 0.32 | 101.1 | 0.93053 | 3.28 | 2.29 | 3.49 | 2.97 | 3.01 | 0.45 | 94.4 | 0.65310 | 3.50 | 2.40 | | 3.58 | 3.16 | 0.54 | 79.0 | 0.20148 |
| AS 2.5 µM | 1.97 | 1.52 | 2.11 | 1.71 | 1.83 | 0.23 | 107.5 | 0.48464 | 2.65 | 2.13 | 3.29 | 2.58 | 2.66 | 0.41 | 83.5 | 0.20156 | 3.71 | 2.39 | 3.89 | 2.98 | 3.24 | 0.60 | 81.1 | 0.21886 |
| AS 5 µM | 1.58 | 1.37 | 1.80 | 1.72 | 1.62 | 0.16 | 95.1 | 0.58796 | 2.33 | 1.46 | 2.85 | 2.10 | 2.19 | 0.50 | 68.5 | 0.04588 | 2.08 | 1.12 | 4.09 | 3.18 | 2.62 | 1.12 | 65.4 | 0.13233 |

## Slide 3
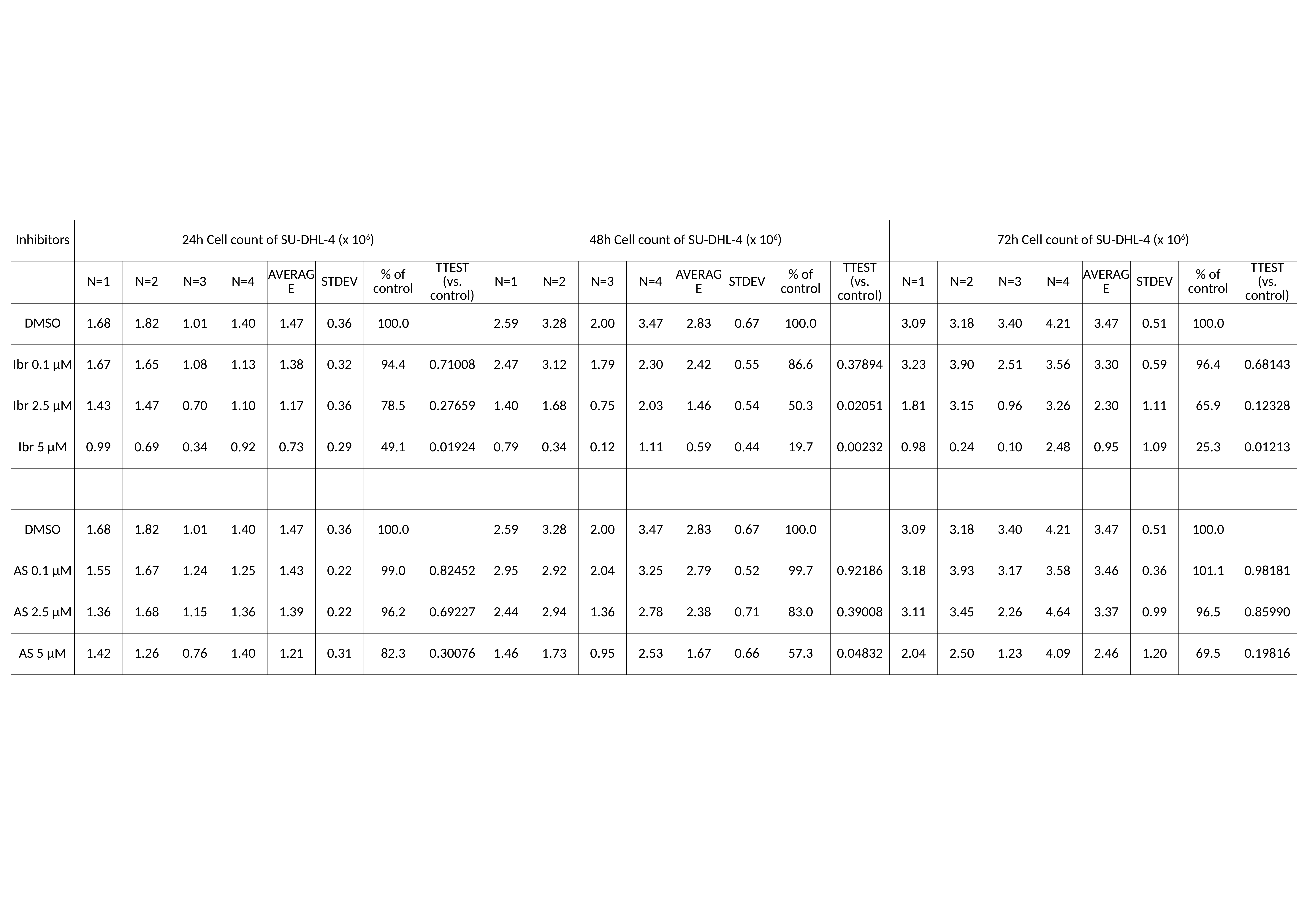

| Inhibitors | 24h Cell count of SU-DHL-4 (x 106) | | | | | | | | 48h Cell count of SU-DHL-4 (x 106) | | | | | | | | 72h Cell count of SU-DHL-4 (x 106) | | | | | | | |
| --- | --- | --- | --- | --- | --- | --- | --- | --- | --- | --- | --- | --- | --- | --- | --- | --- | --- | --- | --- | --- | --- | --- | --- | --- |
| | N=1 | N=2 | N=3 | N=4 | AVERAGE | STDEV | % of control | TTEST (vs. control) | N=1 | N=2 | N=3 | N=4 | AVERAGE | STDEV | % of control | TTEST (vs. control) | N=1 | N=2 | N=3 | N=4 | AVERAGE | STDEV | % of control | TTEST (vs. control) |
| DMSO | 1.68 | 1.82 | 1.01 | 1.40 | 1.47 | 0.36 | 100.0 | | 2.59 | 3.28 | 2.00 | 3.47 | 2.83 | 0.67 | 100.0 | | 3.09 | 3.18 | 3.40 | 4.21 | 3.47 | 0.51 | 100.0 | |
| Ibr 0.1 µM | 1.67 | 1.65 | 1.08 | 1.13 | 1.38 | 0.32 | 94.4 | 0.71008 | 2.47 | 3.12 | 1.79 | 2.30 | 2.42 | 0.55 | 86.6 | 0.37894 | 3.23 | 3.90 | 2.51 | 3.56 | 3.30 | 0.59 | 96.4 | 0.68143 |
| Ibr 2.5 µM | 1.43 | 1.47 | 0.70 | 1.10 | 1.17 | 0.36 | 78.5 | 0.27659 | 1.40 | 1.68 | 0.75 | 2.03 | 1.46 | 0.54 | 50.3 | 0.02051 | 1.81 | 3.15 | 0.96 | 3.26 | 2.30 | 1.11 | 65.9 | 0.12328 |
| Ibr 5 µM | 0.99 | 0.69 | 0.34 | 0.92 | 0.73 | 0.29 | 49.1 | 0.01924 | 0.79 | 0.34 | 0.12 | 1.11 | 0.59 | 0.44 | 19.7 | 0.00232 | 0.98 | 0.24 | 0.10 | 2.48 | 0.95 | 1.09 | 25.3 | 0.01213 |
| | | | | | | | | | | | | | | | | | | | | | | | | |
| DMSO | 1.68 | 1.82 | 1.01 | 1.40 | 1.47 | 0.36 | 100.0 | | 2.59 | 3.28 | 2.00 | 3.47 | 2.83 | 0.67 | 100.0 | | 3.09 | 3.18 | 3.40 | 4.21 | 3.47 | 0.51 | 100.0 | |
| AS 0.1 µM | 1.55 | 1.67 | 1.24 | 1.25 | 1.43 | 0.22 | 99.0 | 0.82452 | 2.95 | 2.92 | 2.04 | 3.25 | 2.79 | 0.52 | 99.7 | 0.92186 | 3.18 | 3.93 | 3.17 | 3.58 | 3.46 | 0.36 | 101.1 | 0.98181 |
| AS 2.5 µM | 1.36 | 1.68 | 1.15 | 1.36 | 1.39 | 0.22 | 96.2 | 0.69227 | 2.44 | 2.94 | 1.36 | 2.78 | 2.38 | 0.71 | 83.0 | 0.39008 | 3.11 | 3.45 | 2.26 | 4.64 | 3.37 | 0.99 | 96.5 | 0.85990 |
| AS 5 µM | 1.42 | 1.26 | 0.76 | 1.40 | 1.21 | 0.31 | 82.3 | 0.30076 | 1.46 | 1.73 | 0.95 | 2.53 | 1.67 | 0.66 | 57.3 | 0.04832 | 2.04 | 2.50 | 1.23 | 4.09 | 2.46 | 1.20 | 69.5 | 0.19816 |

## Slide 4
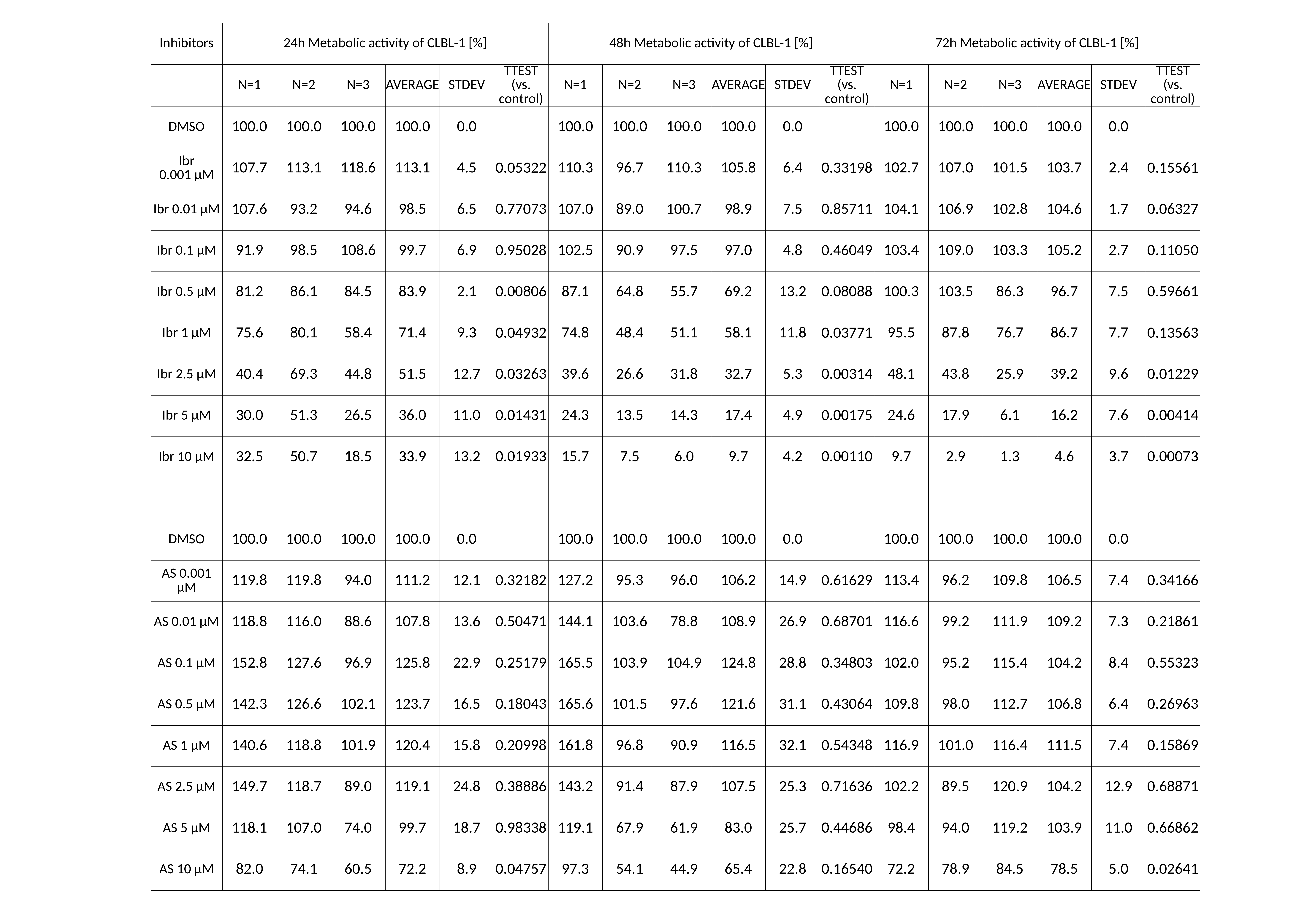

| Inhibitors | 24h Metabolic activity of CLBL-1 [%] | | | | | | 48h Metabolic activity of CLBL-1 [%] | | | | | | 72h Metabolic activity of CLBL-1 [%] | | | | | |
| --- | --- | --- | --- | --- | --- | --- | --- | --- | --- | --- | --- | --- | --- | --- | --- | --- | --- | --- |
| | N=1 | N=2 | N=3 | AVERAGE | STDEV | TTEST (vs. control) | N=1 | N=2 | N=3 | AVERAGE | STDEV | TTEST (vs. control) | N=1 | N=2 | N=3 | AVERAGE | STDEV | TTEST (vs. control) |
| DMSO | 100.0 | 100.0 | 100.0 | 100.0 | 0.0 | | 100.0 | 100.0 | 100.0 | 100.0 | 0.0 | | 100.0 | 100.0 | 100.0 | 100.0 | 0.0 | |
| Ibr 0.001 µM | 107.7 | 113.1 | 118.6 | 113.1 | 4.5 | 0.05322 | 110.3 | 96.7 | 110.3 | 105.8 | 6.4 | 0.33198 | 102.7 | 107.0 | 101.5 | 103.7 | 2.4 | 0.15561 |
| Ibr 0.01 µM | 107.6 | 93.2 | 94.6 | 98.5 | 6.5 | 0.77073 | 107.0 | 89.0 | 100.7 | 98.9 | 7.5 | 0.85711 | 104.1 | 106.9 | 102.8 | 104.6 | 1.7 | 0.06327 |
| Ibr 0.1 µM | 91.9 | 98.5 | 108.6 | 99.7 | 6.9 | 0.95028 | 102.5 | 90.9 | 97.5 | 97.0 | 4.8 | 0.46049 | 103.4 | 109.0 | 103.3 | 105.2 | 2.7 | 0.11050 |
| Ibr 0.5 µM | 81.2 | 86.1 | 84.5 | 83.9 | 2.1 | 0.00806 | 87.1 | 64.8 | 55.7 | 69.2 | 13.2 | 0.08088 | 100.3 | 103.5 | 86.3 | 96.7 | 7.5 | 0.59661 |
| Ibr 1 µM | 75.6 | 80.1 | 58.4 | 71.4 | 9.3 | 0.04932 | 74.8 | 48.4 | 51.1 | 58.1 | 11.8 | 0.03771 | 95.5 | 87.8 | 76.7 | 86.7 | 7.7 | 0.13563 |
| Ibr 2.5 µM | 40.4 | 69.3 | 44.8 | 51.5 | 12.7 | 0.03263 | 39.6 | 26.6 | 31.8 | 32.7 | 5.3 | 0.00314 | 48.1 | 43.8 | 25.9 | 39.2 | 9.6 | 0.01229 |
| Ibr 5 µM | 30.0 | 51.3 | 26.5 | 36.0 | 11.0 | 0.01431 | 24.3 | 13.5 | 14.3 | 17.4 | 4.9 | 0.00175 | 24.6 | 17.9 | 6.1 | 16.2 | 7.6 | 0.00414 |
| Ibr 10 µM | 32.5 | 50.7 | 18.5 | 33.9 | 13.2 | 0.01933 | 15.7 | 7.5 | 6.0 | 9.7 | 4.2 | 0.00110 | 9.7 | 2.9 | 1.3 | 4.6 | 3.7 | 0.00073 |
| | | | | | | | | | | | | | | | | | | |
| DMSO | 100.0 | 100.0 | 100.0 | 100.0 | 0.0 | | 100.0 | 100.0 | 100.0 | 100.0 | 0.0 | | 100.0 | 100.0 | 100.0 | 100.0 | 0.0 | |
| AS 0.001 µM | 119.8 | 119.8 | 94.0 | 111.2 | 12.1 | 0.32182 | 127.2 | 95.3 | 96.0 | 106.2 | 14.9 | 0.61629 | 113.4 | 96.2 | 109.8 | 106.5 | 7.4 | 0.34166 |
| AS 0.01 µM | 118.8 | 116.0 | 88.6 | 107.8 | 13.6 | 0.50471 | 144.1 | 103.6 | 78.8 | 108.9 | 26.9 | 0.68701 | 116.6 | 99.2 | 111.9 | 109.2 | 7.3 | 0.21861 |
| AS 0.1 µM | 152.8 | 127.6 | 96.9 | 125.8 | 22.9 | 0.25179 | 165.5 | 103.9 | 104.9 | 124.8 | 28.8 | 0.34803 | 102.0 | 95.2 | 115.4 | 104.2 | 8.4 | 0.55323 |
| AS 0.5 µM | 142.3 | 126.6 | 102.1 | 123.7 | 16.5 | 0.18043 | 165.6 | 101.5 | 97.6 | 121.6 | 31.1 | 0.43064 | 109.8 | 98.0 | 112.7 | 106.8 | 6.4 | 0.26963 |
| AS 1 µM | 140.6 | 118.8 | 101.9 | 120.4 | 15.8 | 0.20998 | 161.8 | 96.8 | 90.9 | 116.5 | 32.1 | 0.54348 | 116.9 | 101.0 | 116.4 | 111.5 | 7.4 | 0.15869 |
| AS 2.5 µM | 149.7 | 118.7 | 89.0 | 119.1 | 24.8 | 0.38886 | 143.2 | 91.4 | 87.9 | 107.5 | 25.3 | 0.71636 | 102.2 | 89.5 | 120.9 | 104.2 | 12.9 | 0.68871 |
| AS 5 µM | 118.1 | 107.0 | 74.0 | 99.7 | 18.7 | 0.98338 | 119.1 | 67.9 | 61.9 | 83.0 | 25.7 | 0.44686 | 98.4 | 94.0 | 119.2 | 103.9 | 11.0 | 0.66862 |
| AS 10 µM | 82.0 | 74.1 | 60.5 | 72.2 | 8.9 | 0.04757 | 97.3 | 54.1 | 44.9 | 65.4 | 22.8 | 0.16540 | 72.2 | 78.9 | 84.5 | 78.5 | 5.0 | 0.02641 |

## Slide 5
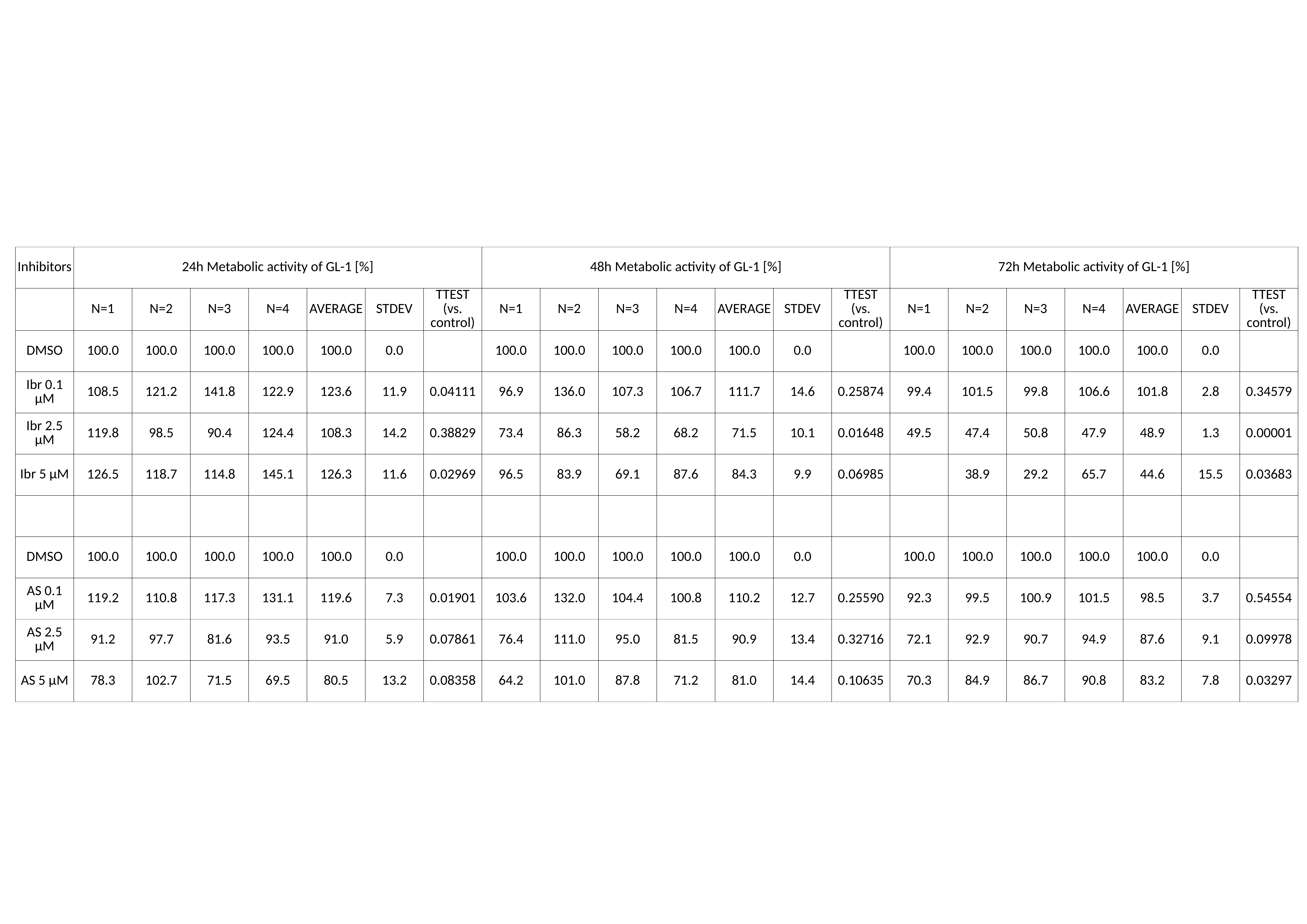

| Inhibitors | 24h Metabolic activity of GL-1 [%] | | | | | | | 48h Metabolic activity of GL-1 [%] | | | | | | | 72h Metabolic activity of GL-1 [%] | | | | | | |
| --- | --- | --- | --- | --- | --- | --- | --- | --- | --- | --- | --- | --- | --- | --- | --- | --- | --- | --- | --- | --- | --- |
| | N=1 | N=2 | N=3 | N=4 | AVERAGE | STDEV | TTEST (vs. control) | N=1 | N=2 | N=3 | N=4 | AVERAGE | STDEV | TTEST (vs. control) | N=1 | N=2 | N=3 | N=4 | AVERAGE | STDEV | TTEST (vs. control) |
| DMSO | 100.0 | 100.0 | 100.0 | 100.0 | 100.0 | 0.0 | | 100.0 | 100.0 | 100.0 | 100.0 | 100.0 | 0.0 | | 100.0 | 100.0 | 100.0 | 100.0 | 100.0 | 0.0 | |
| Ibr 0.1 µM | 108.5 | 121.2 | 141.8 | 122.9 | 123.6 | 11.9 | 0.04111 | 96.9 | 136.0 | 107.3 | 106.7 | 111.7 | 14.6 | 0.25874 | 99.4 | 101.5 | 99.8 | 106.6 | 101.8 | 2.8 | 0.34579 |
| Ibr 2.5 µM | 119.8 | 98.5 | 90.4 | 124.4 | 108.3 | 14.2 | 0.38829 | 73.4 | 86.3 | 58.2 | 68.2 | 71.5 | 10.1 | 0.01648 | 49.5 | 47.4 | 50.8 | 47.9 | 48.9 | 1.3 | 0.00001 |
| Ibr 5 µM | 126.5 | 118.7 | 114.8 | 145.1 | 126.3 | 11.6 | 0.02969 | 96.5 | 83.9 | 69.1 | 87.6 | 84.3 | 9.9 | 0.06985 | | 38.9 | 29.2 | 65.7 | 44.6 | 15.5 | 0.03683 |
| | | | | | | | | | | | | | | | | | | | | | |
| DMSO | 100.0 | 100.0 | 100.0 | 100.0 | 100.0 | 0.0 | | 100.0 | 100.0 | 100.0 | 100.0 | 100.0 | 0.0 | | 100.0 | 100.0 | 100.0 | 100.0 | 100.0 | 0.0 | |
| AS 0.1 µM | 119.2 | 110.8 | 117.3 | 131.1 | 119.6 | 7.3 | 0.01901 | 103.6 | 132.0 | 104.4 | 100.8 | 110.2 | 12.7 | 0.25590 | 92.3 | 99.5 | 100.9 | 101.5 | 98.5 | 3.7 | 0.54554 |
| AS 2.5 µM | 91.2 | 97.7 | 81.6 | 93.5 | 91.0 | 5.9 | 0.07861 | 76.4 | 111.0 | 95.0 | 81.5 | 90.9 | 13.4 | 0.32716 | 72.1 | 92.9 | 90.7 | 94.9 | 87.6 | 9.1 | 0.09978 |
| AS 5 µM | 78.3 | 102.7 | 71.5 | 69.5 | 80.5 | 13.2 | 0.08358 | 64.2 | 101.0 | 87.8 | 71.2 | 81.0 | 14.4 | 0.10635 | 70.3 | 84.9 | 86.7 | 90.8 | 83.2 | 7.8 | 0.03297 |

## Slide 6
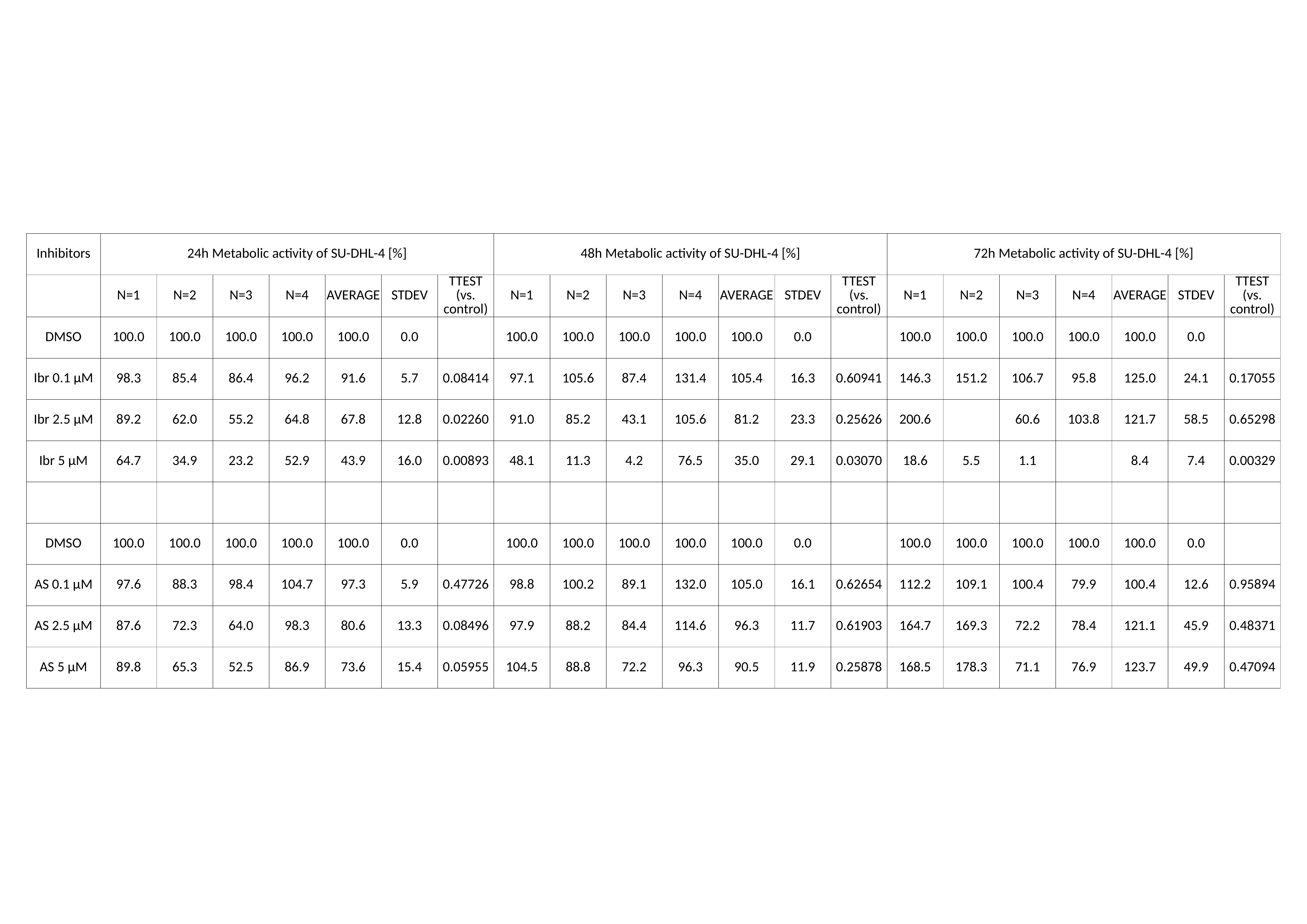

| Inhibitors | 24h Metabolic activity of SU-DHL-4 [%] | | | | | | | 48h Metabolic activity of SU-DHL-4 [%] | | | | | | | 72h Metabolic activity of SU-DHL-4 [%] | | | | | | |
| --- | --- | --- | --- | --- | --- | --- | --- | --- | --- | --- | --- | --- | --- | --- | --- | --- | --- | --- | --- | --- | --- |
| | N=1 | N=2 | N=3 | N=4 | AVERAGE | STDEV | TTEST (vs. control) | N=1 | N=2 | N=3 | N=4 | AVERAGE | STDEV | TTEST (vs. control) | N=1 | N=2 | N=3 | N=4 | AVERAGE | STDEV | TTEST (vs. control) |
| DMSO | 100.0 | 100.0 | 100.0 | 100.0 | 100.0 | 0.0 | | 100.0 | 100.0 | 100.0 | 100.0 | 100.0 | 0.0 | | 100.0 | 100.0 | 100.0 | 100.0 | 100.0 | 0.0 | |
| Ibr 0.1 µM | 98.3 | 85.4 | 86.4 | 96.2 | 91.6 | 5.7 | 0.08414 | 97.1 | 105.6 | 87.4 | 131.4 | 105.4 | 16.3 | 0.60941 | 146.3 | 151.2 | 106.7 | 95.8 | 125.0 | 24.1 | 0.17055 |
| Ibr 2.5 µM | 89.2 | 62.0 | 55.2 | 64.8 | 67.8 | 12.8 | 0.02260 | 91.0 | 85.2 | 43.1 | 105.6 | 81.2 | 23.3 | 0.25626 | 200.6 | | 60.6 | 103.8 | 121.7 | 58.5 | 0.65298 |
| Ibr 5 µM | 64.7 | 34.9 | 23.2 | 52.9 | 43.9 | 16.0 | 0.00893 | 48.1 | 11.3 | 4.2 | 76.5 | 35.0 | 29.1 | 0.03070 | 18.6 | 5.5 | 1.1 | | 8.4 | 7.4 | 0.00329 |
| | | | | | | | | | | | | | | | | | | | | | |
| DMSO | 100.0 | 100.0 | 100.0 | 100.0 | 100.0 | 0.0 | | 100.0 | 100.0 | 100.0 | 100.0 | 100.0 | 0.0 | | 100.0 | 100.0 | 100.0 | 100.0 | 100.0 | 0.0 | |
| AS 0.1 µM | 97.6 | 88.3 | 98.4 | 104.7 | 97.3 | 5.9 | 0.47726 | 98.8 | 100.2 | 89.1 | 132.0 | 105.0 | 16.1 | 0.62654 | 112.2 | 109.1 | 100.4 | 79.9 | 100.4 | 12.6 | 0.95894 |
| AS 2.5 µM | 87.6 | 72.3 | 64.0 | 98.3 | 80.6 | 13.3 | 0.08496 | 97.9 | 88.2 | 84.4 | 114.6 | 96.3 | 11.7 | 0.61903 | 164.7 | 169.3 | 72.2 | 78.4 | 121.1 | 45.9 | 0.48371 |
| AS 5 µM | 89.8 | 65.3 | 52.5 | 86.9 | 73.6 | 15.4 | 0.05955 | 104.5 | 88.8 | 72.2 | 96.3 | 90.5 | 11.9 | 0.25878 | 168.5 | 178.3 | 71.1 | 76.9 | 123.7 | 49.9 | 0.47094 |

## Slide 7
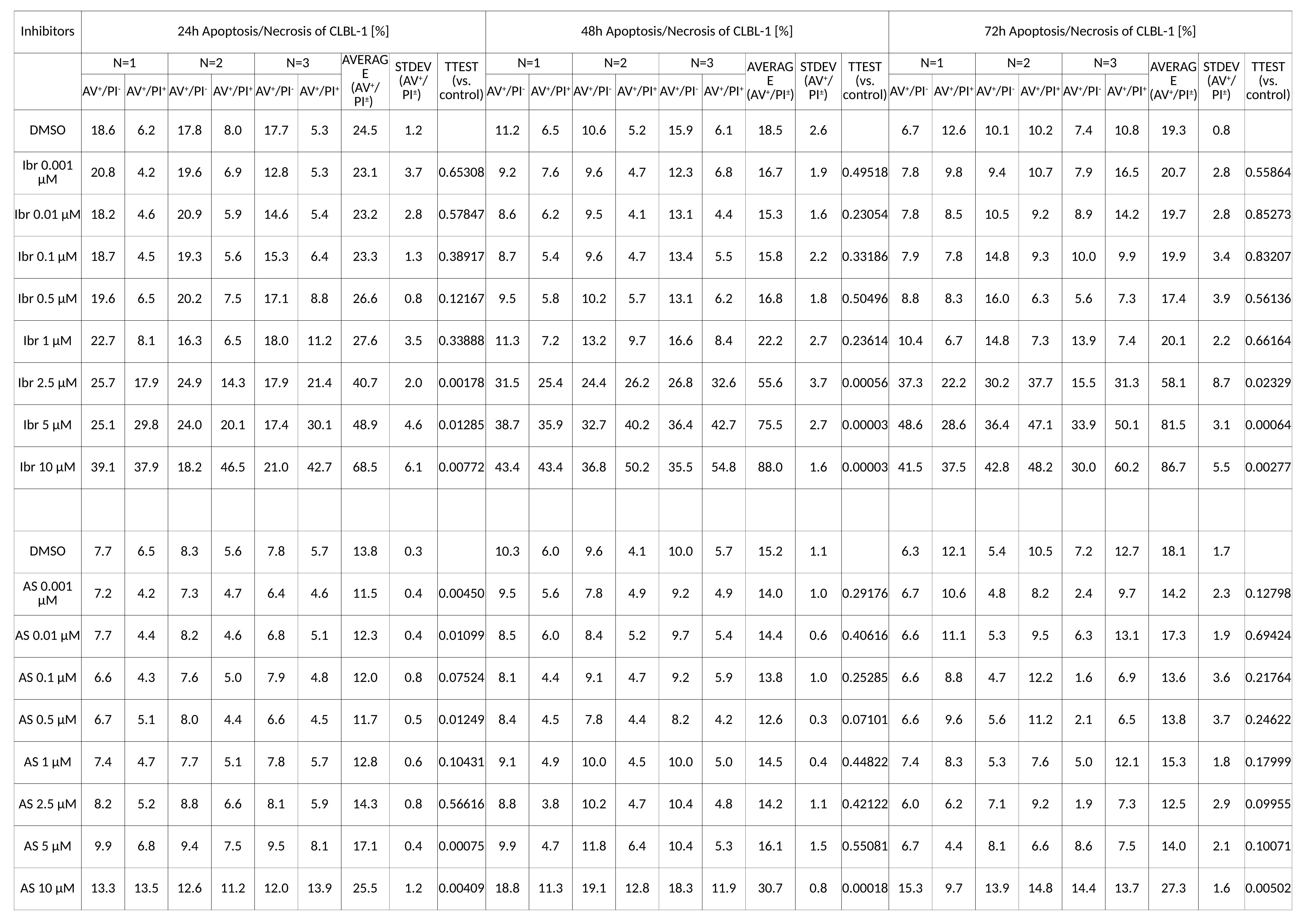

| Inhibitors | 24h Apoptosis/Necrosis of CLBL-1 [%] | | | | | | | | | 48h Apoptosis/Necrosis of CLBL-1 [%] | | | | | | | | | 72h Apoptosis/Necrosis of CLBL-1 [%] | | | | | | | | |
| --- | --- | --- | --- | --- | --- | --- | --- | --- | --- | --- | --- | --- | --- | --- | --- | --- | --- | --- | --- | --- | --- | --- | --- | --- | --- | --- | --- |
| | N=1 | | N=2 | | N=3 | | AVERAGE (AV+/PI±) | STDEV (AV+/PI±) | TTEST (vs. control) | N=1 | | N=2 | | N=3 | | AVERAGE (AV+/PI±) | STDEV (AV+/PI±) | TTEST (vs. control) | N=1 | | N=2 | | N=3 | | AVERAGE (AV+/PI±) | STDEV (AV+/PI±) | TTEST (vs. control) |
| | AV+/PI- | AV+/PI+ | AV+/PI- | AV+/PI+ | AV+/PI- | AV+/PI+ | | | | AV+/PI- | AV+/PI+ | AV+/PI- | AV+/PI+ | AV+/PI- | AV+/PI+ | | | | AV+/PI- | AV+/PI+ | AV+/PI- | AV+/PI+ | AV+/PI- | AV+/PI+ | | | |
| DMSO | 18.6 | 6.2 | 17.8 | 8.0 | 17.7 | 5.3 | 24.5 | 1.2 | | 11.2 | 6.5 | 10.6 | 5.2 | 15.9 | 6.1 | 18.5 | 2.6 | | 6.7 | 12.6 | 10.1 | 10.2 | 7.4 | 10.8 | 19.3 | 0.8 | |
| Ibr 0.001 µM | 20.8 | 4.2 | 19.6 | 6.9 | 12.8 | 5.3 | 23.1 | 3.7 | 0.65308 | 9.2 | 7.6 | 9.6 | 4.7 | 12.3 | 6.8 | 16.7 | 1.9 | 0.49518 | 7.8 | 9.8 | 9.4 | 10.7 | 7.9 | 16.5 | 20.7 | 2.8 | 0.55864 |
| Ibr 0.01 µM | 18.2 | 4.6 | 20.9 | 5.9 | 14.6 | 5.4 | 23.2 | 2.8 | 0.57847 | 8.6 | 6.2 | 9.5 | 4.1 | 13.1 | 4.4 | 15.3 | 1.6 | 0.23054 | 7.8 | 8.5 | 10.5 | 9.2 | 8.9 | 14.2 | 19.7 | 2.8 | 0.85273 |
| Ibr 0.1 µM | 18.7 | 4.5 | 19.3 | 5.6 | 15.3 | 6.4 | 23.3 | 1.3 | 0.38917 | 8.7 | 5.4 | 9.6 | 4.7 | 13.4 | 5.5 | 15.8 | 2.2 | 0.33186 | 7.9 | 7.8 | 14.8 | 9.3 | 10.0 | 9.9 | 19.9 | 3.4 | 0.83207 |
| Ibr 0.5 µM | 19.6 | 6.5 | 20.2 | 7.5 | 17.1 | 8.8 | 26.6 | 0.8 | 0.12167 | 9.5 | 5.8 | 10.2 | 5.7 | 13.1 | 6.2 | 16.8 | 1.8 | 0.50496 | 8.8 | 8.3 | 16.0 | 6.3 | 5.6 | 7.3 | 17.4 | 3.9 | 0.56136 |
| Ibr 1 µM | 22.7 | 8.1 | 16.3 | 6.5 | 18.0 | 11.2 | 27.6 | 3.5 | 0.33888 | 11.3 | 7.2 | 13.2 | 9.7 | 16.6 | 8.4 | 22.2 | 2.7 | 0.23614 | 10.4 | 6.7 | 14.8 | 7.3 | 13.9 | 7.4 | 20.1 | 2.2 | 0.66164 |
| Ibr 2.5 µM | 25.7 | 17.9 | 24.9 | 14.3 | 17.9 | 21.4 | 40.7 | 2.0 | 0.00178 | 31.5 | 25.4 | 24.4 | 26.2 | 26.8 | 32.6 | 55.6 | 3.7 | 0.00056 | 37.3 | 22.2 | 30.2 | 37.7 | 15.5 | 31.3 | 58.1 | 8.7 | 0.02329 |
| Ibr 5 µM | 25.1 | 29.8 | 24.0 | 20.1 | 17.4 | 30.1 | 48.9 | 4.6 | 0.01285 | 38.7 | 35.9 | 32.7 | 40.2 | 36.4 | 42.7 | 75.5 | 2.7 | 0.00003 | 48.6 | 28.6 | 36.4 | 47.1 | 33.9 | 50.1 | 81.5 | 3.1 | 0.00064 |
| Ibr 10 µM | 39.1 | 37.9 | 18.2 | 46.5 | 21.0 | 42.7 | 68.5 | 6.1 | 0.00772 | 43.4 | 43.4 | 36.8 | 50.2 | 35.5 | 54.8 | 88.0 | 1.6 | 0.00003 | 41.5 | 37.5 | 42.8 | 48.2 | 30.0 | 60.2 | 86.7 | 5.5 | 0.00277 |
| | | | | | | | | | | | | | | | | | | | | | | | | | | | |
| DMSO | 7.7 | 6.5 | 8.3 | 5.6 | 7.8 | 5.7 | 13.8 | 0.3 | | 10.3 | 6.0 | 9.6 | 4.1 | 10.0 | 5.7 | 15.2 | 1.1 | | 6.3 | 12.1 | 5.4 | 10.5 | 7.2 | 12.7 | 18.1 | 1.7 | |
| AS 0.001 µM | 7.2 | 4.2 | 7.3 | 4.7 | 6.4 | 4.6 | 11.5 | 0.4 | 0.00450 | 9.5 | 5.6 | 7.8 | 4.9 | 9.2 | 4.9 | 14.0 | 1.0 | 0.29176 | 6.7 | 10.6 | 4.8 | 8.2 | 2.4 | 9.7 | 14.2 | 2.3 | 0.12798 |
| AS 0.01 µM | 7.7 | 4.4 | 8.2 | 4.6 | 6.8 | 5.1 | 12.3 | 0.4 | 0.01099 | 8.5 | 6.0 | 8.4 | 5.2 | 9.7 | 5.4 | 14.4 | 0.6 | 0.40616 | 6.6 | 11.1 | 5.3 | 9.5 | 6.3 | 13.1 | 17.3 | 1.9 | 0.69424 |
| AS 0.1 µM | 6.6 | 4.3 | 7.6 | 5.0 | 7.9 | 4.8 | 12.0 | 0.8 | 0.07524 | 8.1 | 4.4 | 9.1 | 4.7 | 9.2 | 5.9 | 13.8 | 1.0 | 0.25285 | 6.6 | 8.8 | 4.7 | 12.2 | 1.6 | 6.9 | 13.6 | 3.6 | 0.21764 |
| AS 0.5 µM | 6.7 | 5.1 | 8.0 | 4.4 | 6.6 | 4.5 | 11.7 | 0.5 | 0.01249 | 8.4 | 4.5 | 7.8 | 4.4 | 8.2 | 4.2 | 12.6 | 0.3 | 0.07101 | 6.6 | 9.6 | 5.6 | 11.2 | 2.1 | 6.5 | 13.8 | 3.7 | 0.24622 |
| AS 1 µM | 7.4 | 4.7 | 7.7 | 5.1 | 7.8 | 5.7 | 12.8 | 0.6 | 0.10431 | 9.1 | 4.9 | 10.0 | 4.5 | 10.0 | 5.0 | 14.5 | 0.4 | 0.44822 | 7.4 | 8.3 | 5.3 | 7.6 | 5.0 | 12.1 | 15.3 | 1.8 | 0.17999 |
| AS 2.5 µM | 8.2 | 5.2 | 8.8 | 6.6 | 8.1 | 5.9 | 14.3 | 0.8 | 0.56616 | 8.8 | 3.8 | 10.2 | 4.7 | 10.4 | 4.8 | 14.2 | 1.1 | 0.42122 | 6.0 | 6.2 | 7.1 | 9.2 | 1.9 | 7.3 | 12.5 | 2.9 | 0.09955 |
| AS 5 µM | 9.9 | 6.8 | 9.4 | 7.5 | 9.5 | 8.1 | 17.1 | 0.4 | 0.00075 | 9.9 | 4.7 | 11.8 | 6.4 | 10.4 | 5.3 | 16.1 | 1.5 | 0.55081 | 6.7 | 4.4 | 8.1 | 6.6 | 8.6 | 7.5 | 14.0 | 2.1 | 0.10071 |
| AS 10 µM | 13.3 | 13.5 | 12.6 | 11.2 | 12.0 | 13.9 | 25.5 | 1.2 | 0.00409 | 18.8 | 11.3 | 19.1 | 12.8 | 18.3 | 11.9 | 30.7 | 0.8 | 0.00018 | 15.3 | 9.7 | 13.9 | 14.8 | 14.4 | 13.7 | 27.3 | 1.6 | 0.00502 |

## Slide 8
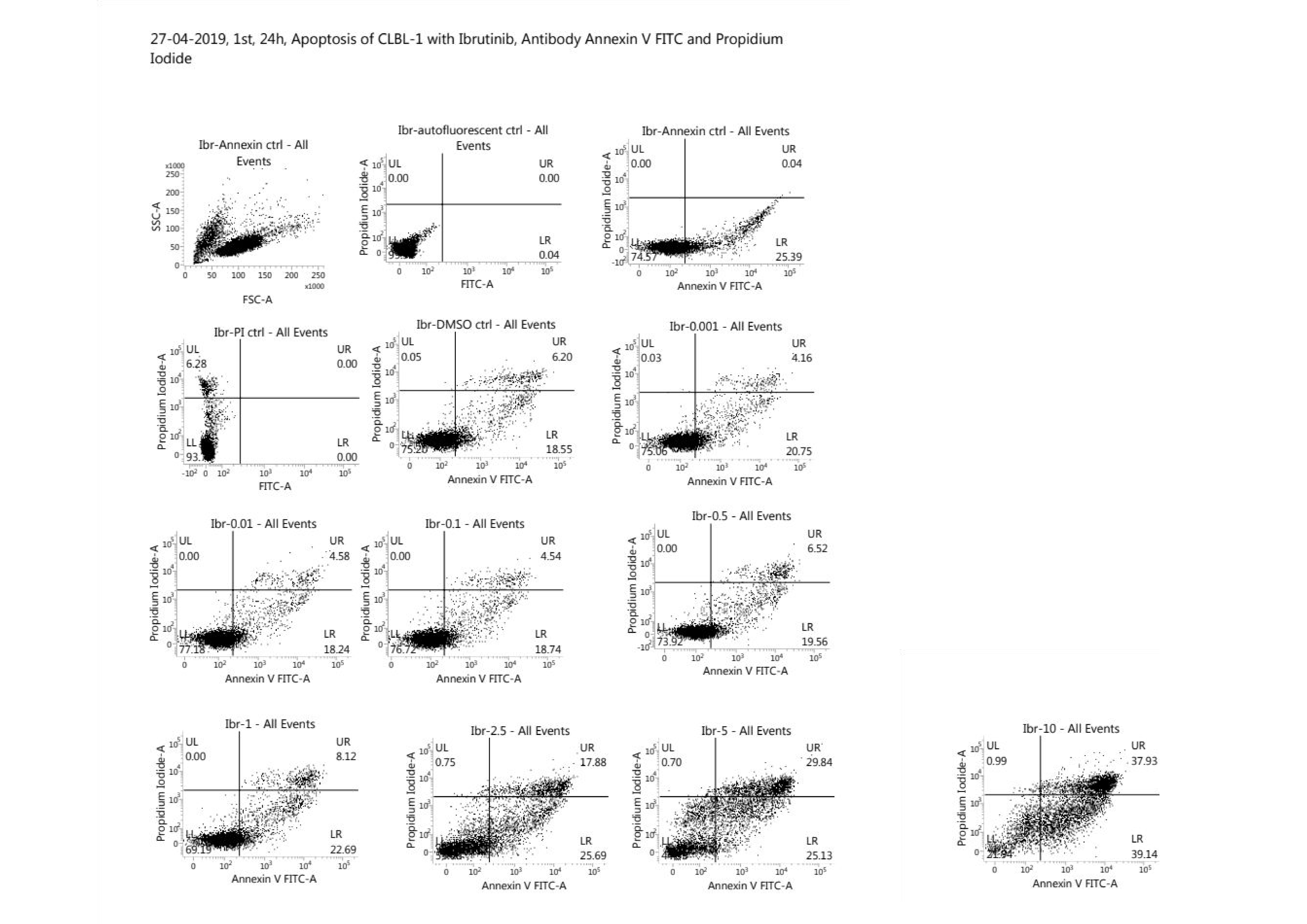

## Slide 9
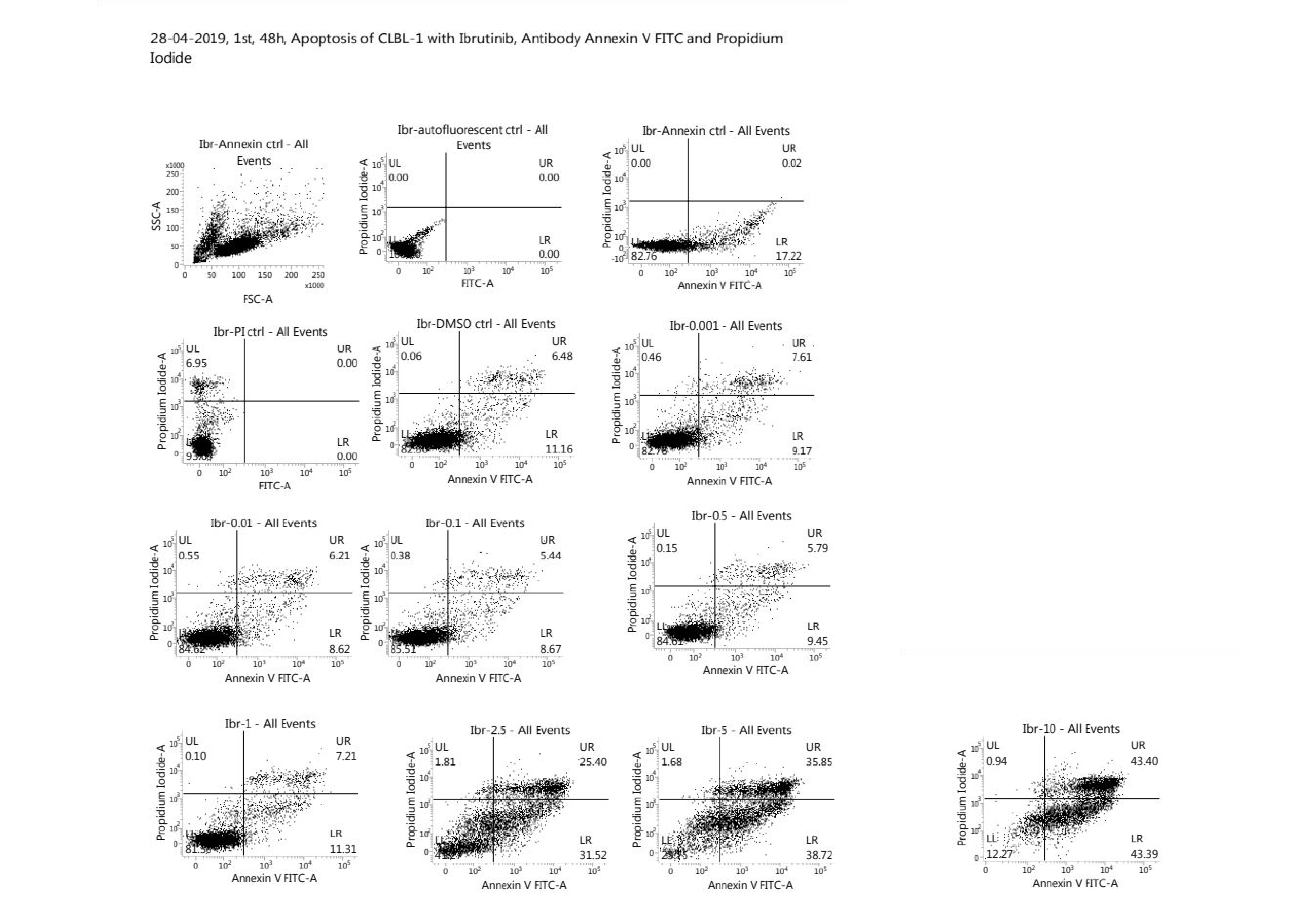

## Slide 10
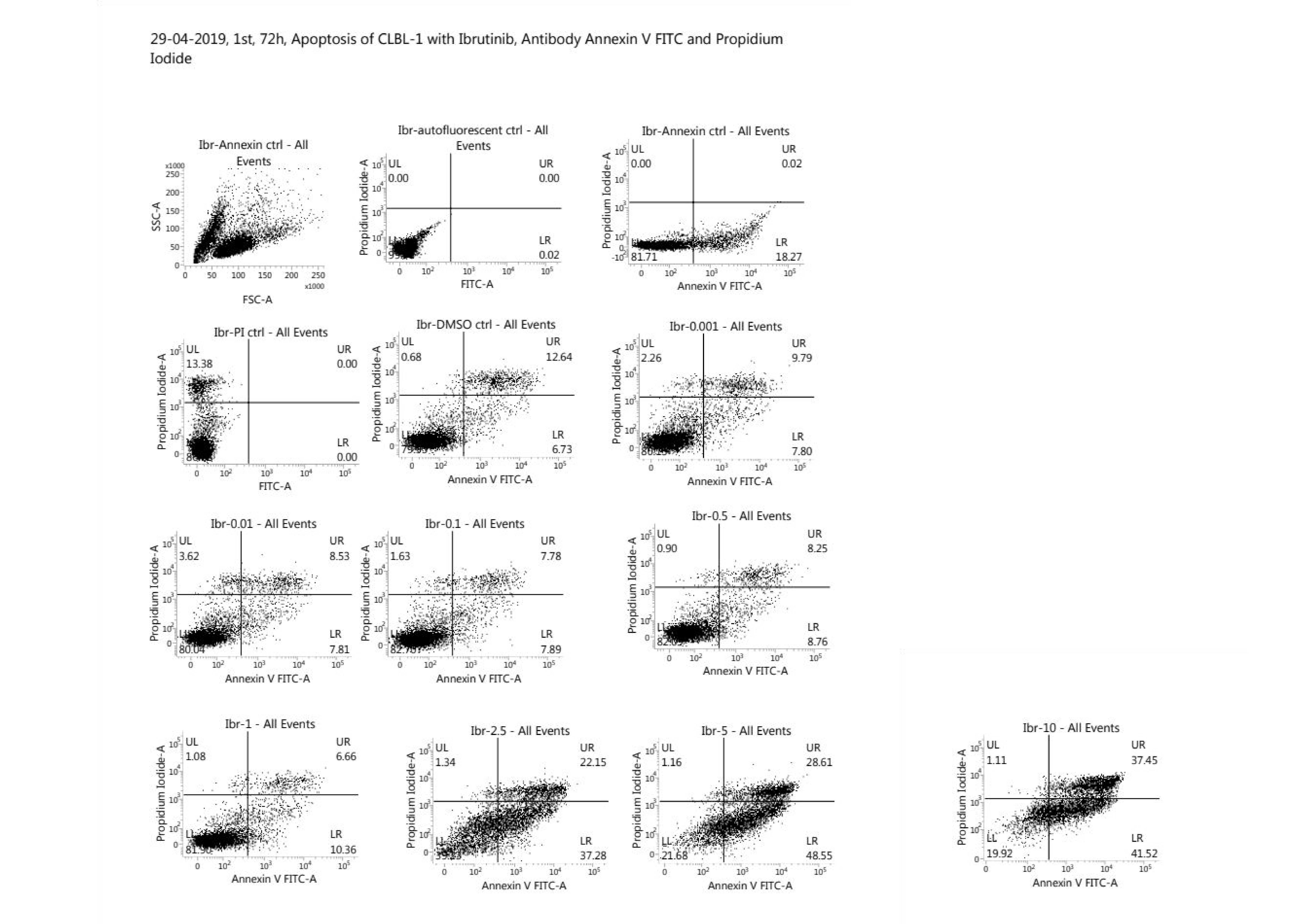

## Slide 11
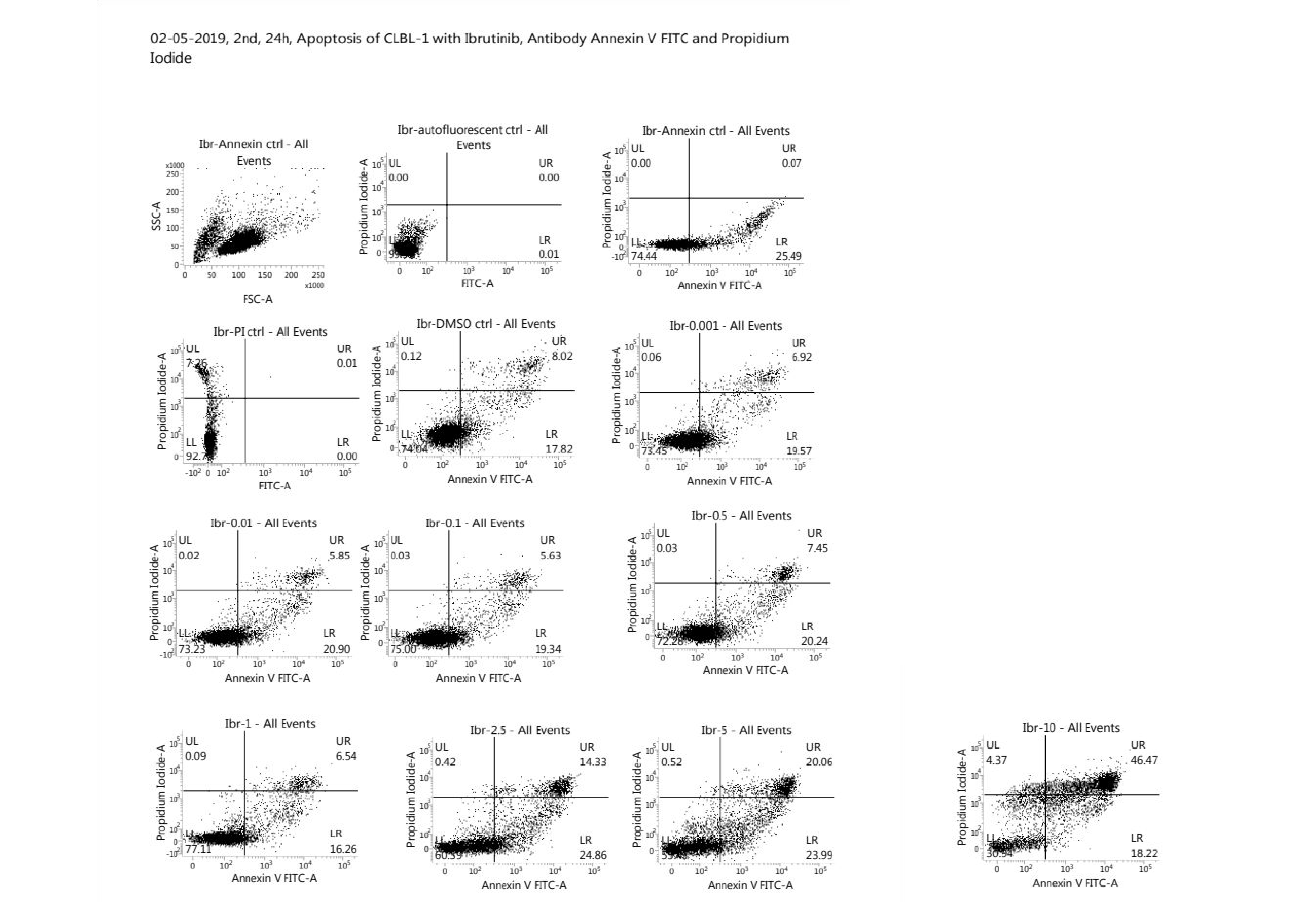

## Slide 12
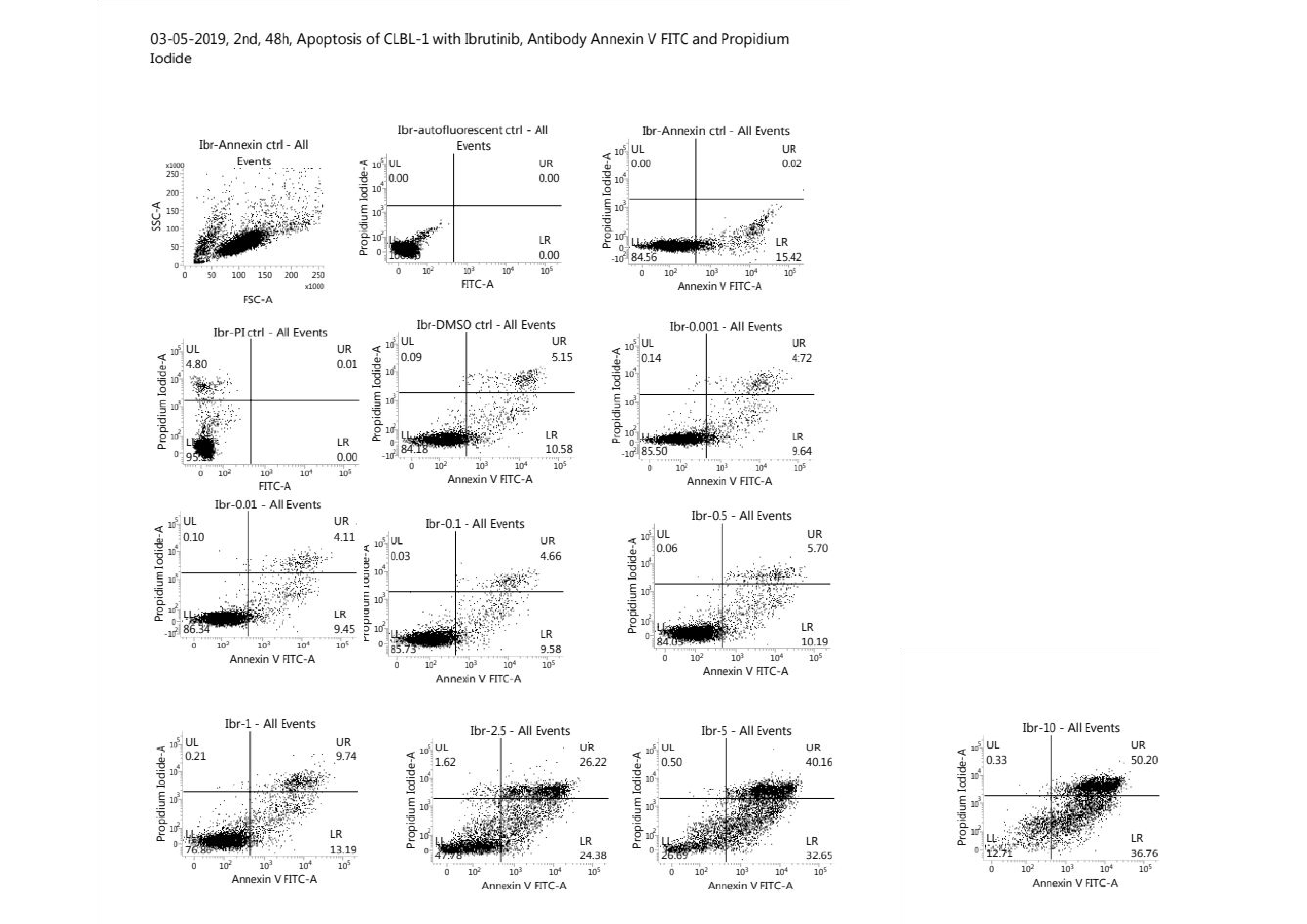

## Slide 13
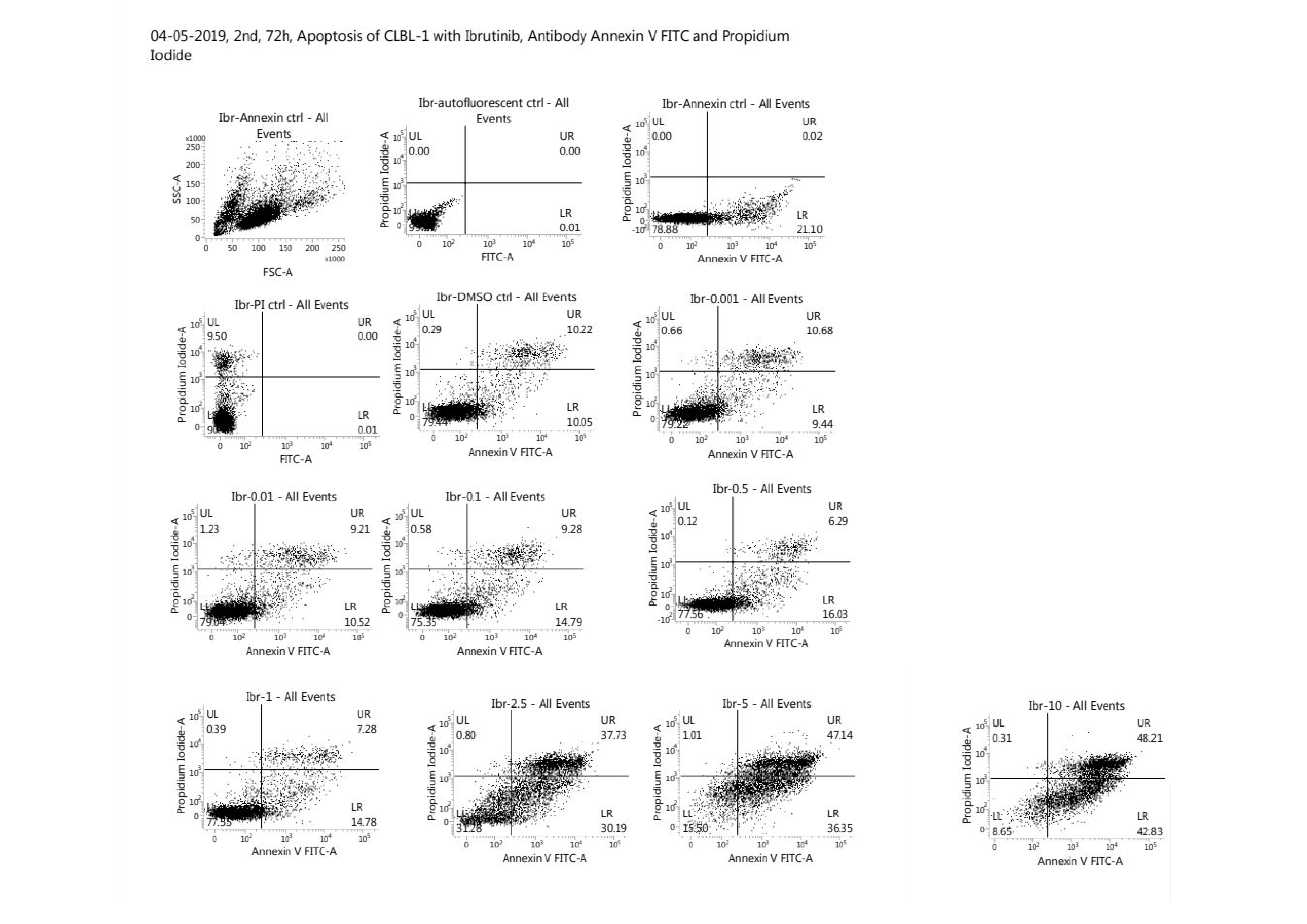

## Slide 14
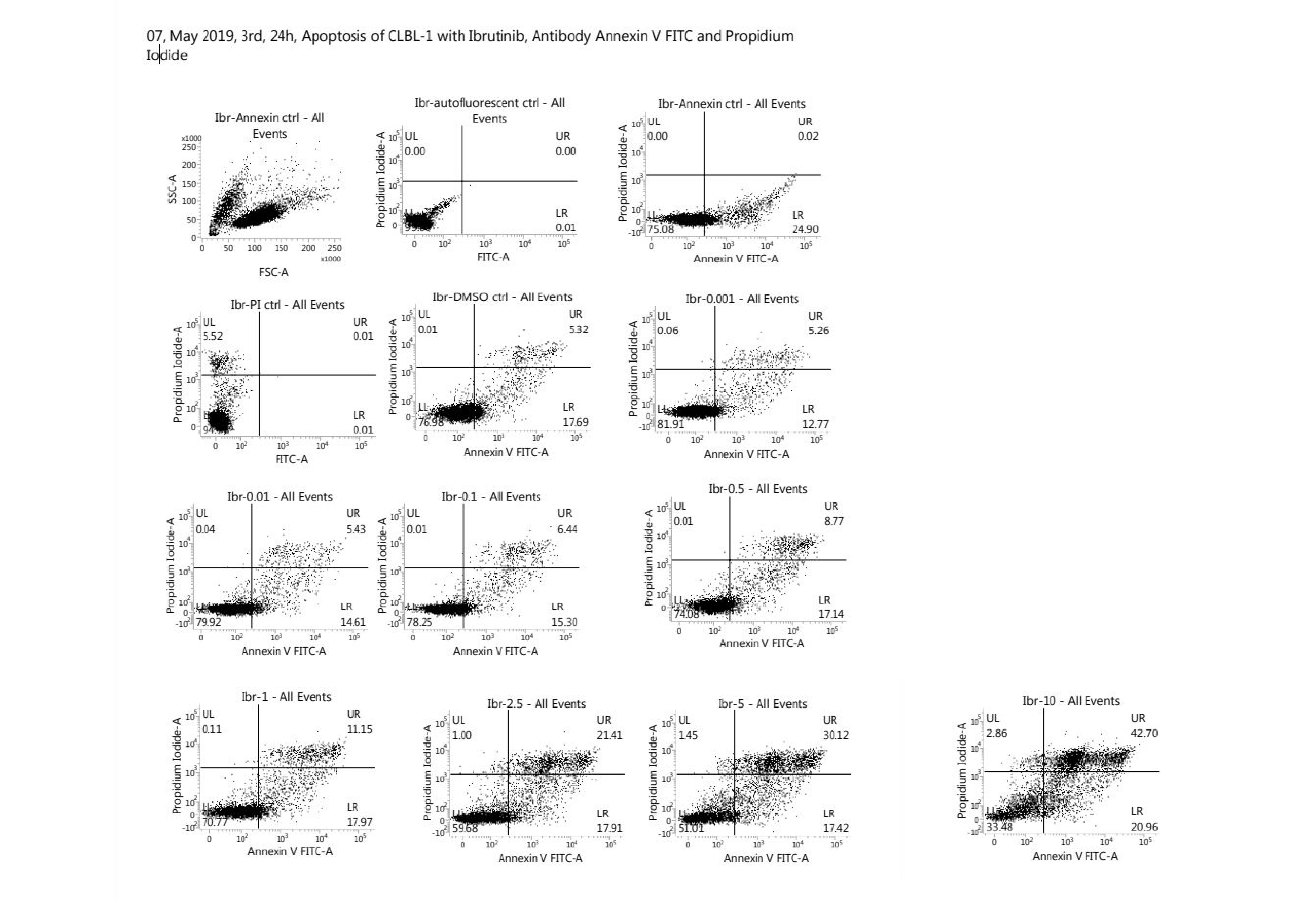

## Slide 15
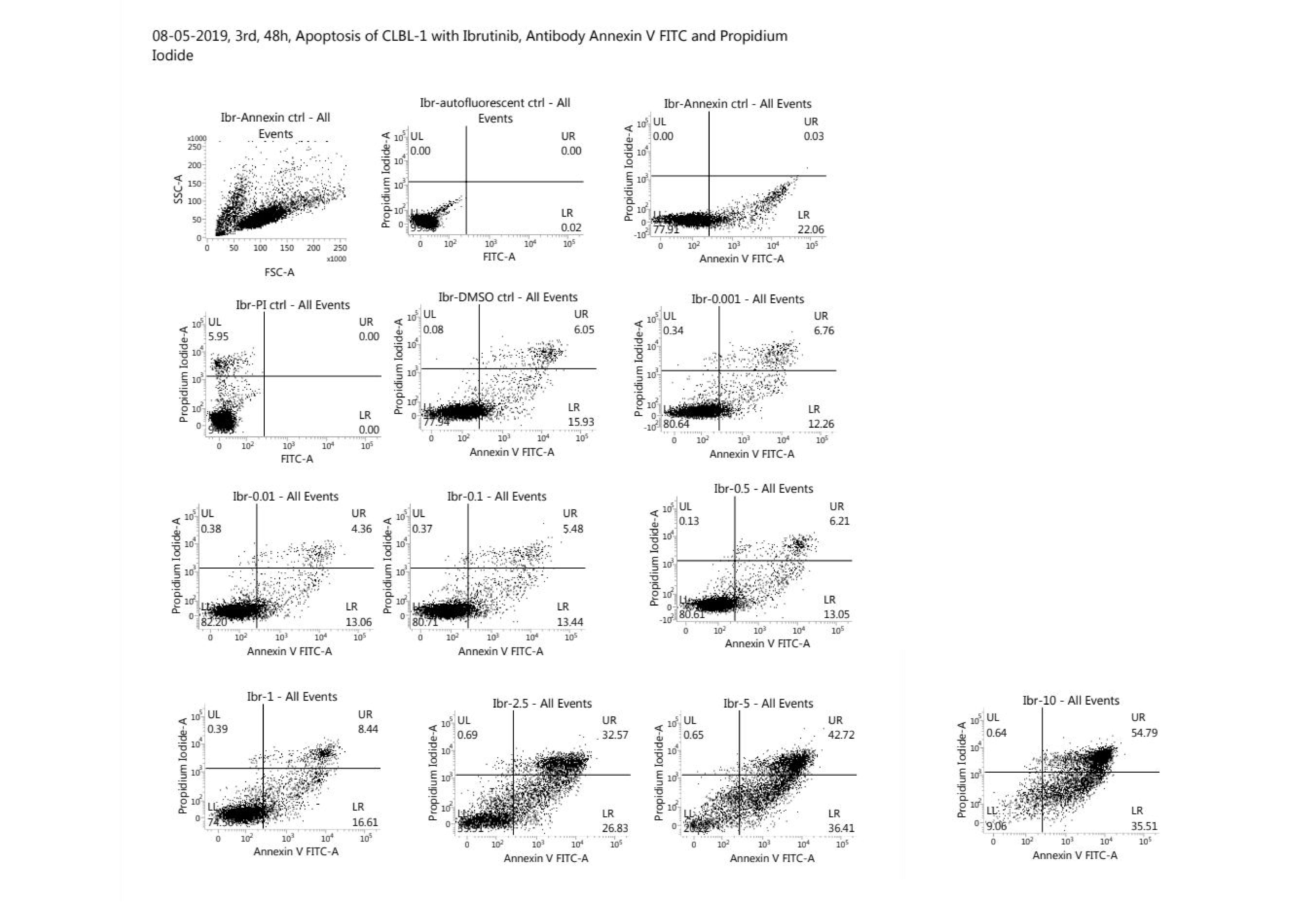

## Slide 16
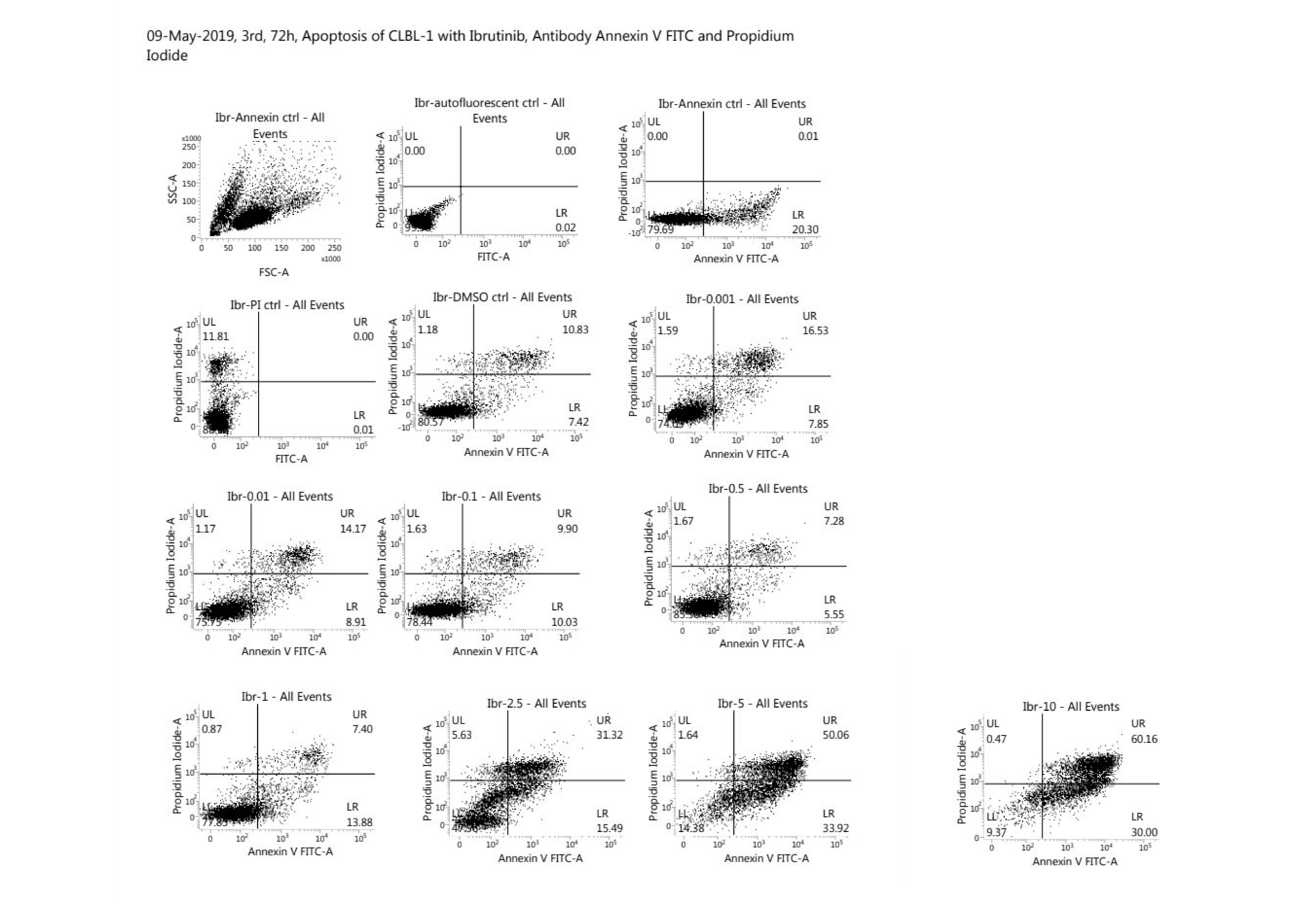

## Slide 17
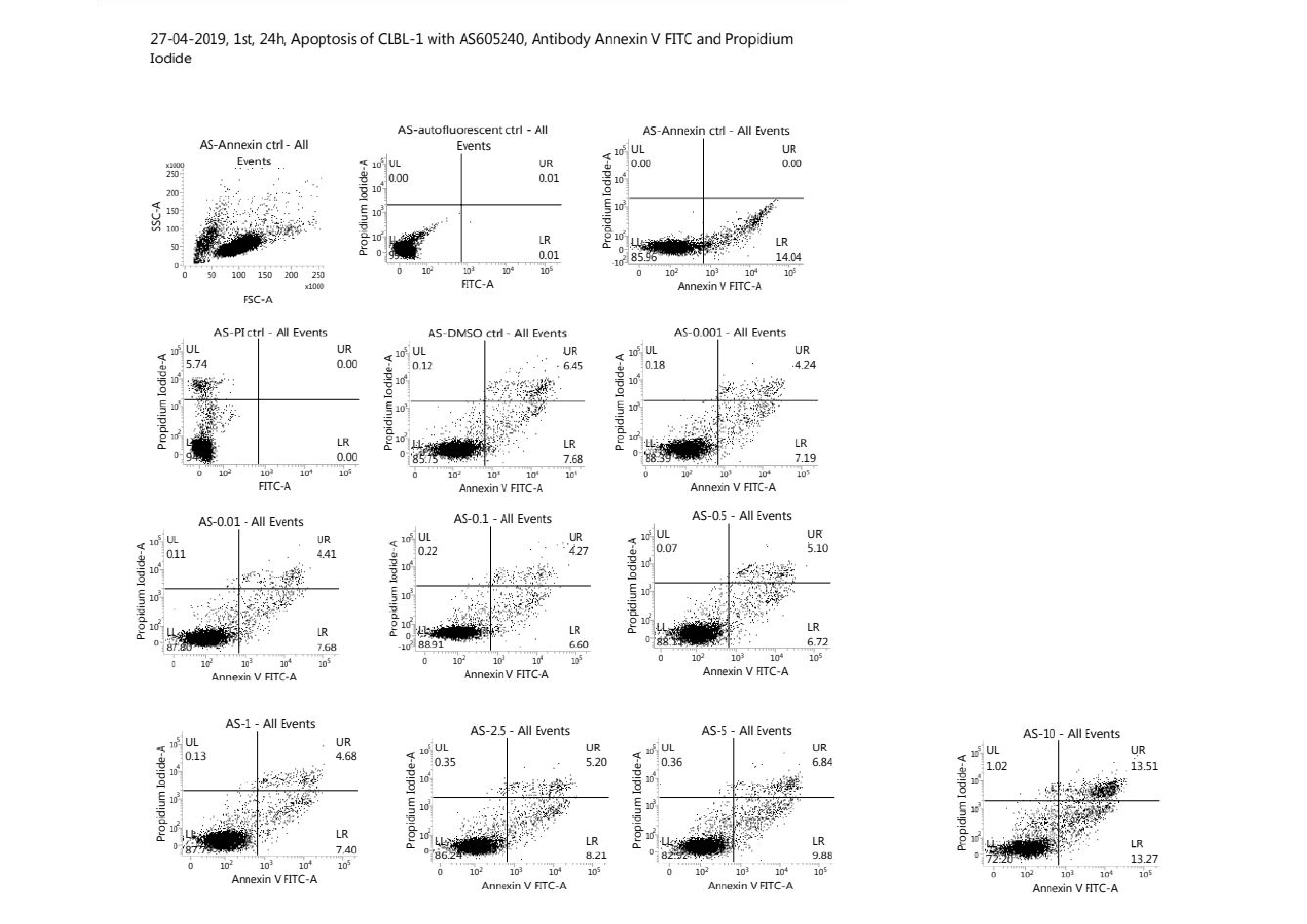

## Slide 18
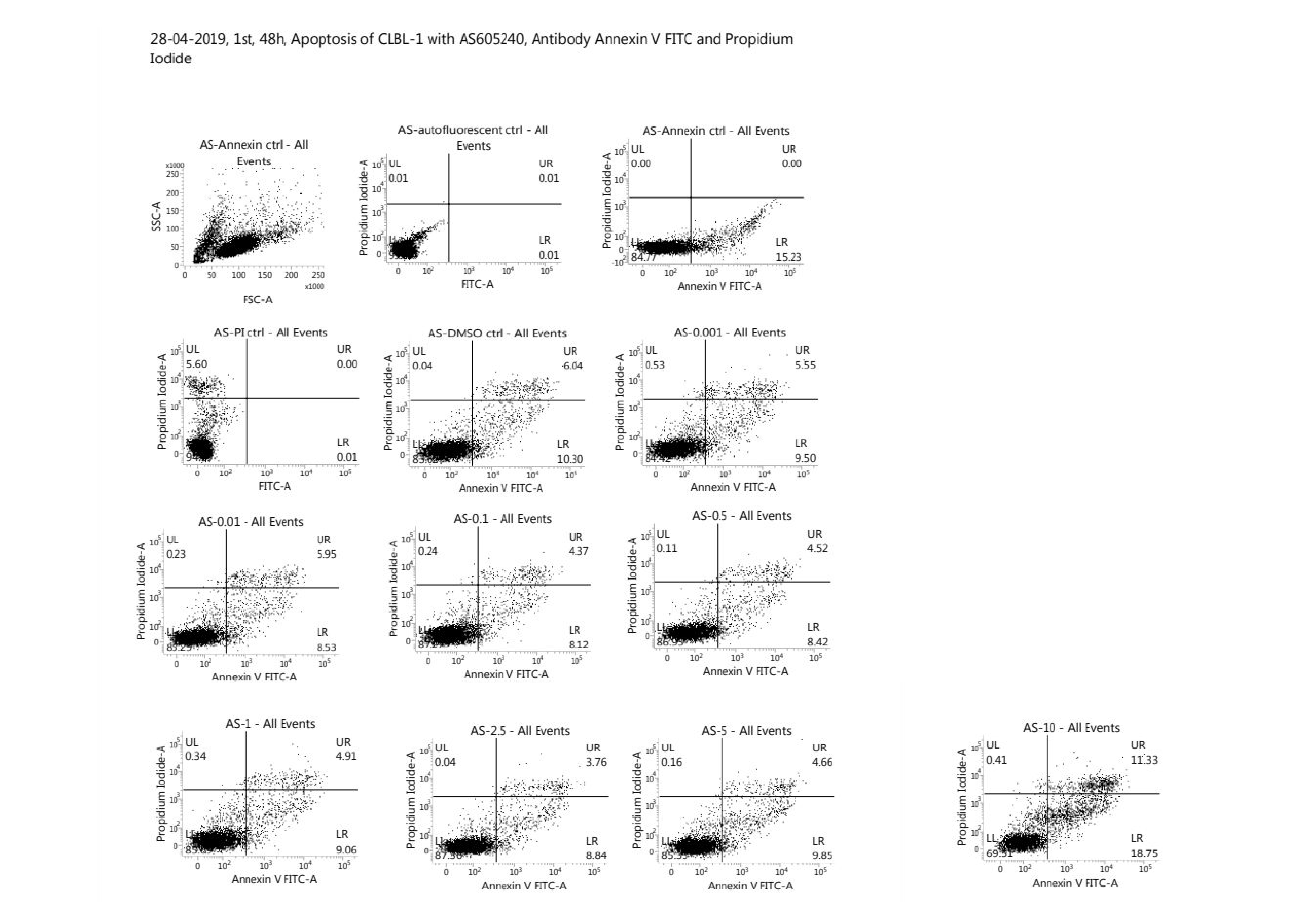

## Slide 19
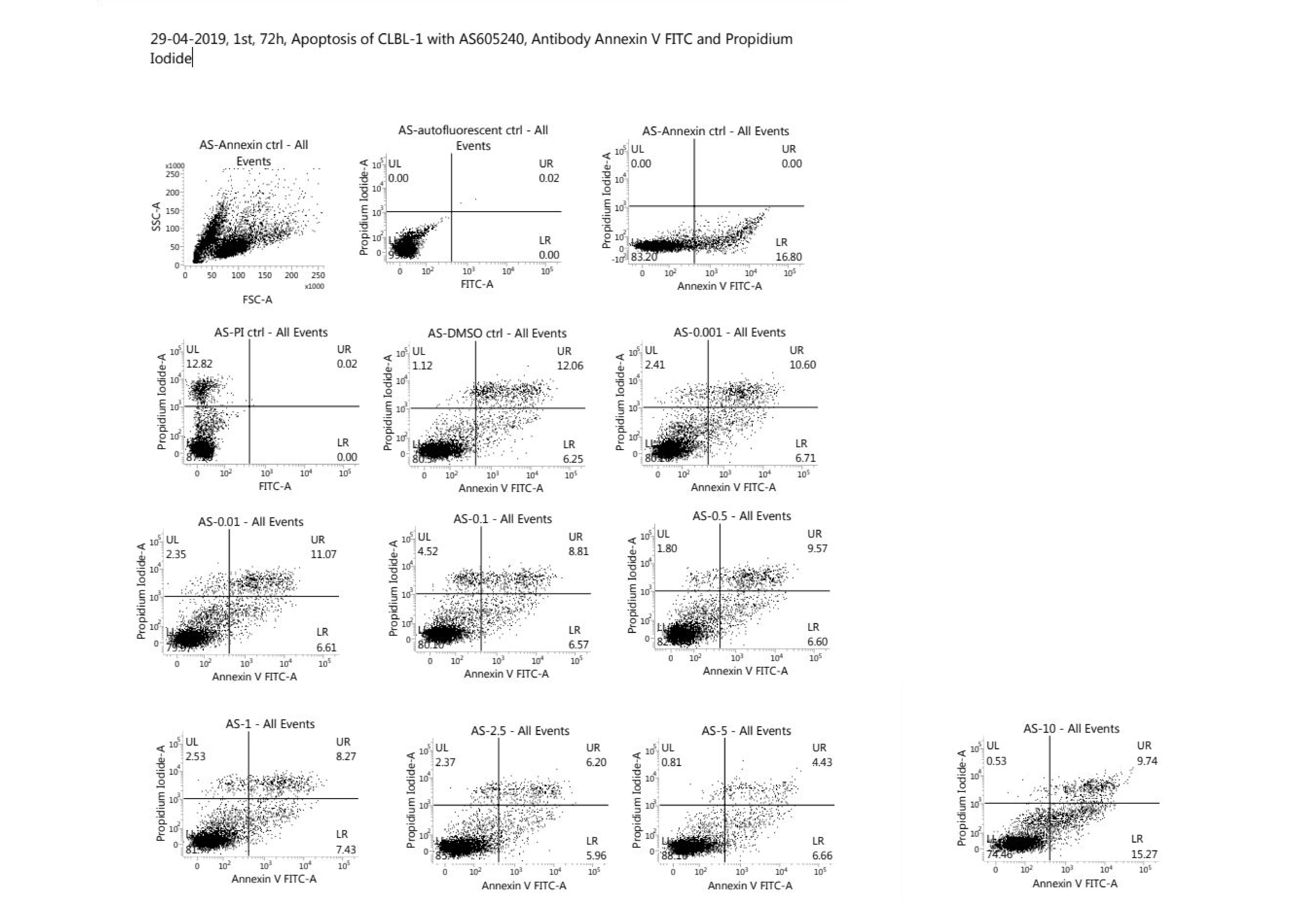

## Slide 20
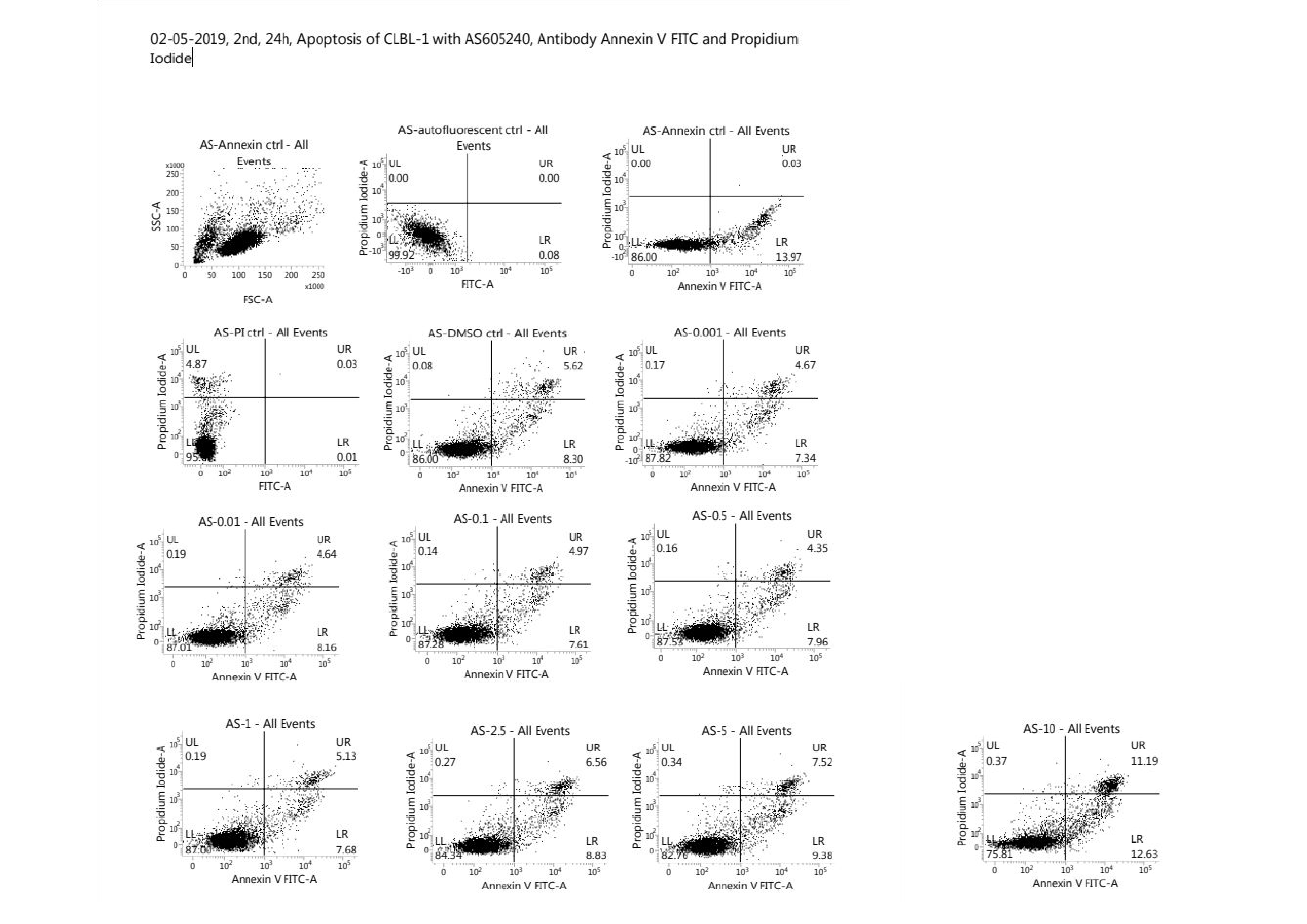

## Slide 21
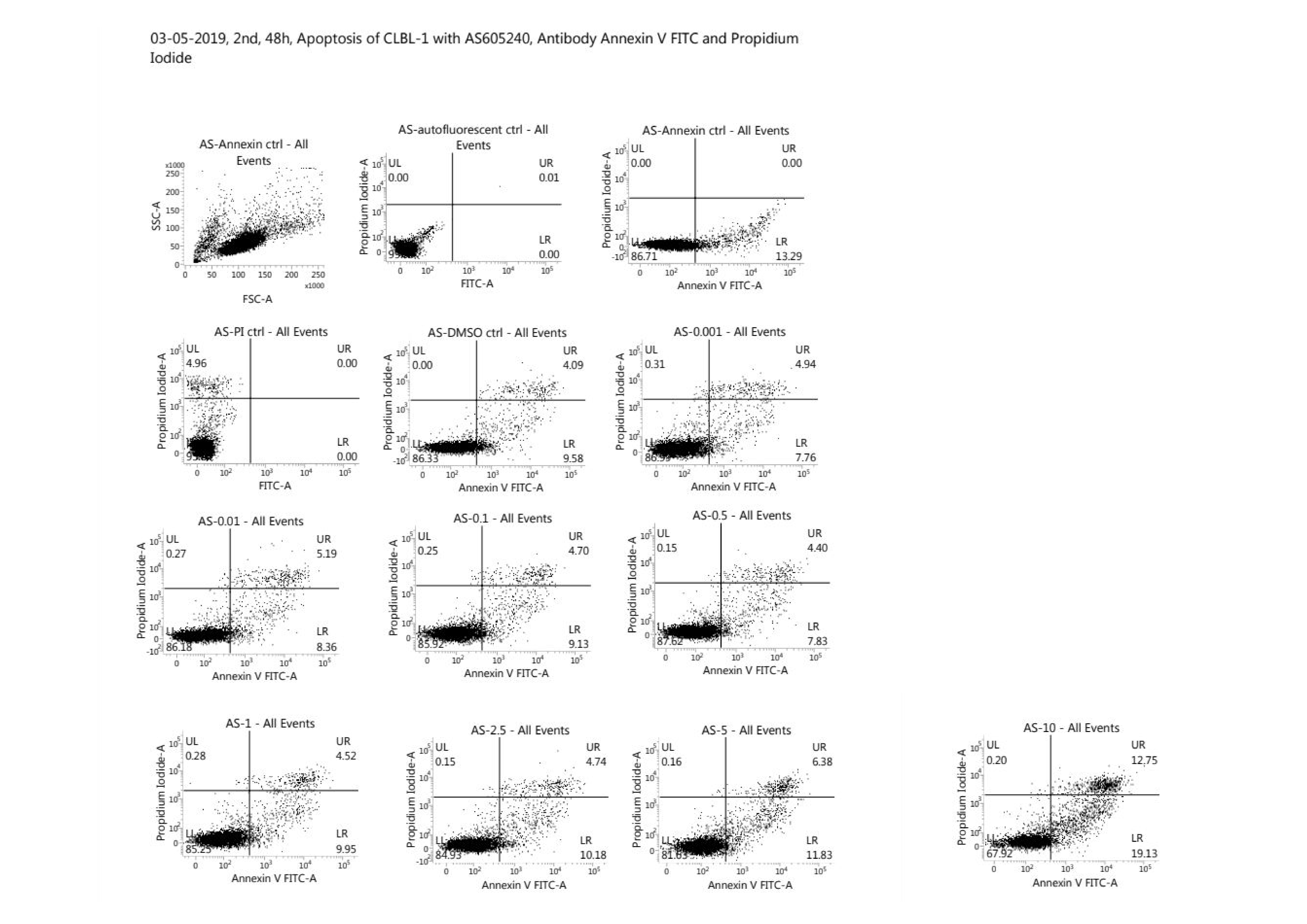

## Slide 22
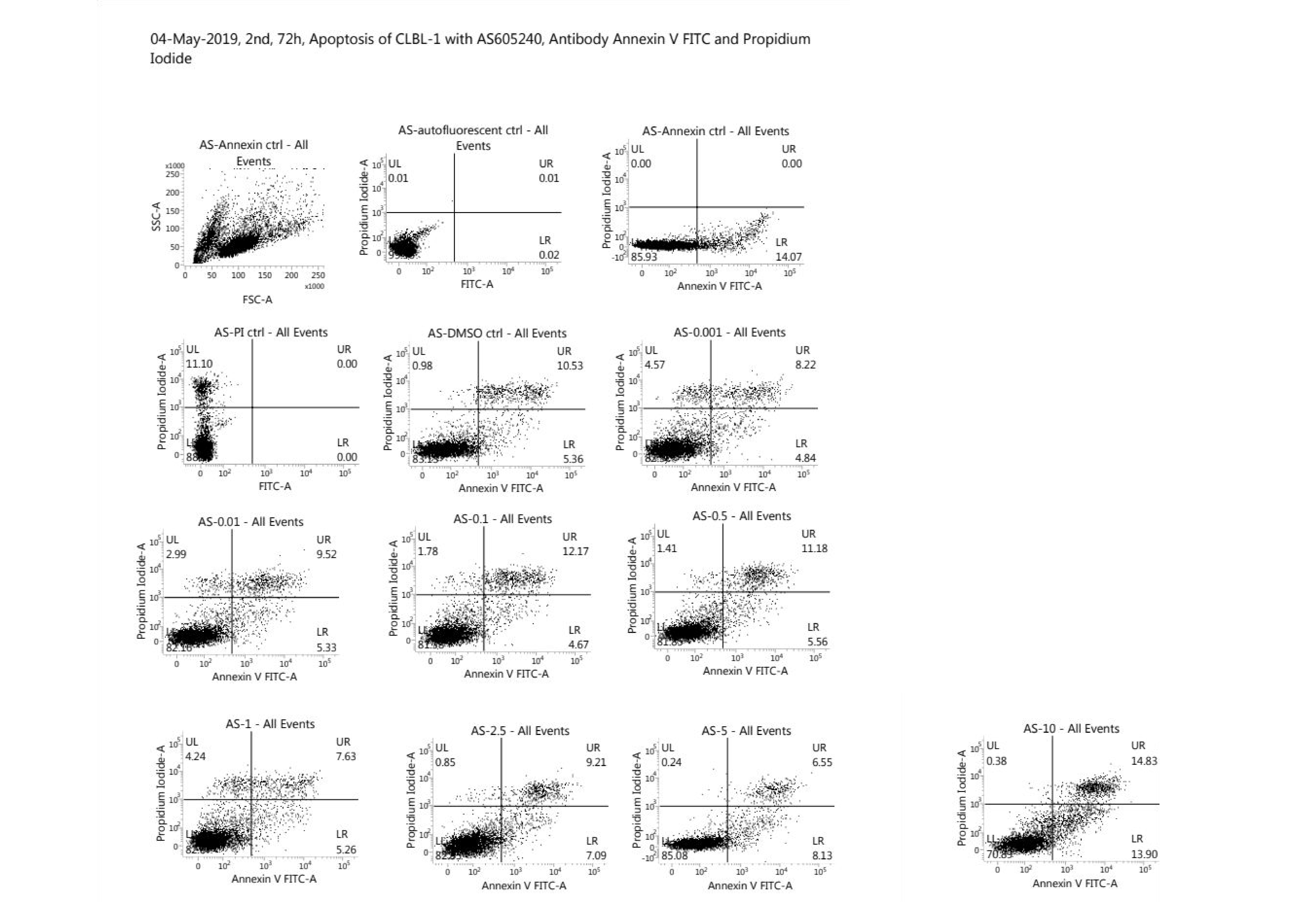

## Slide 23
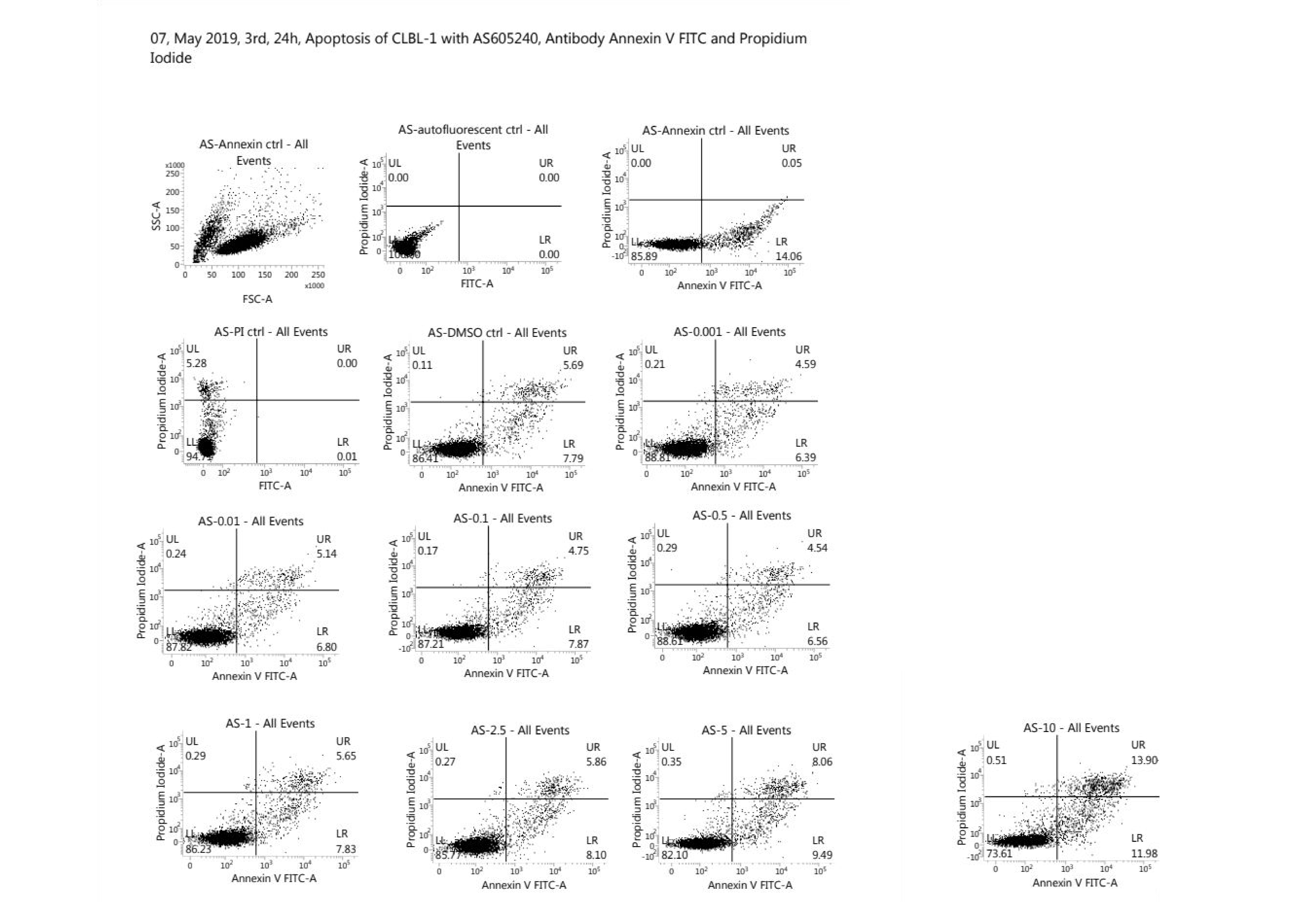

## Slide 24
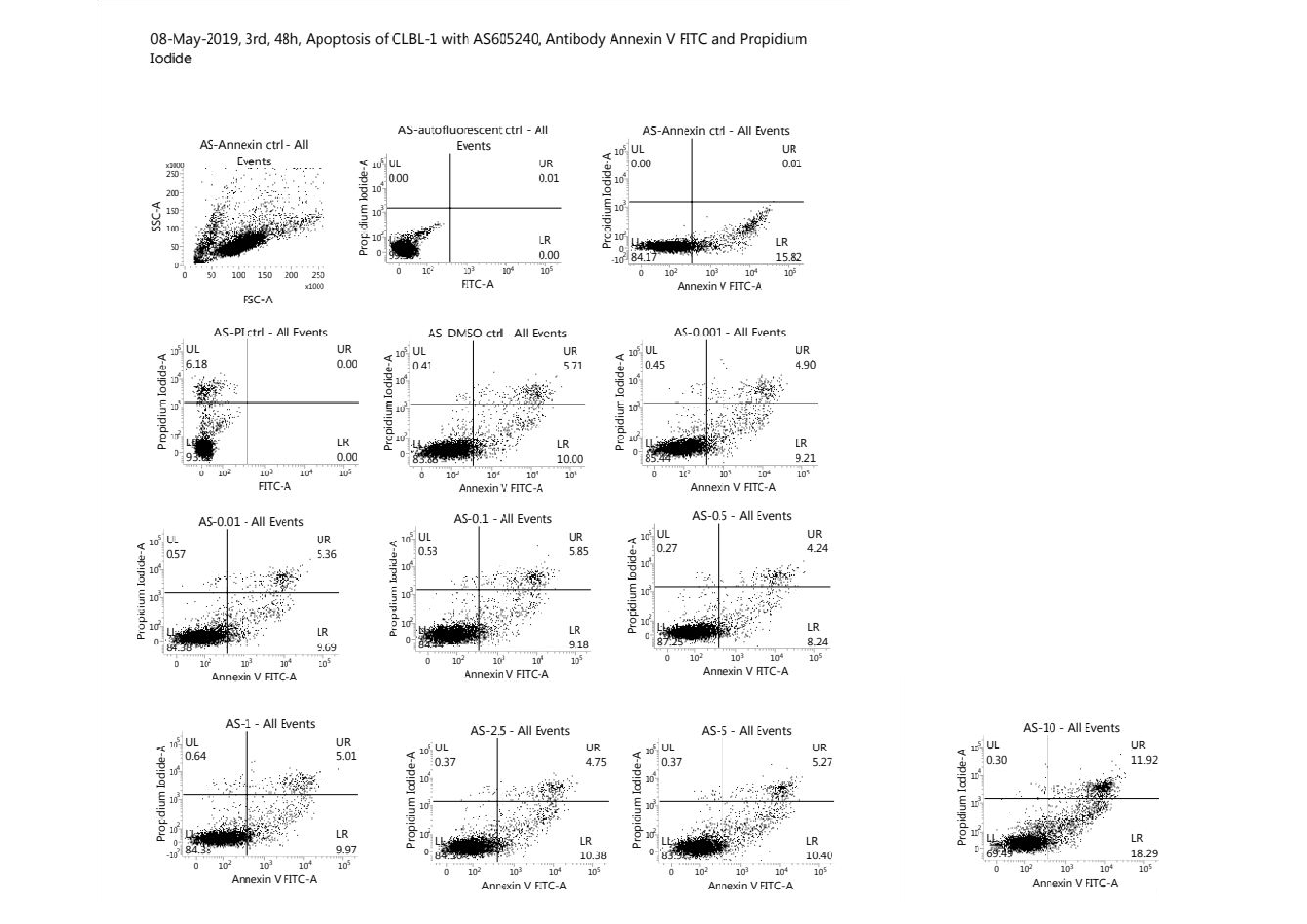

## Slide 25
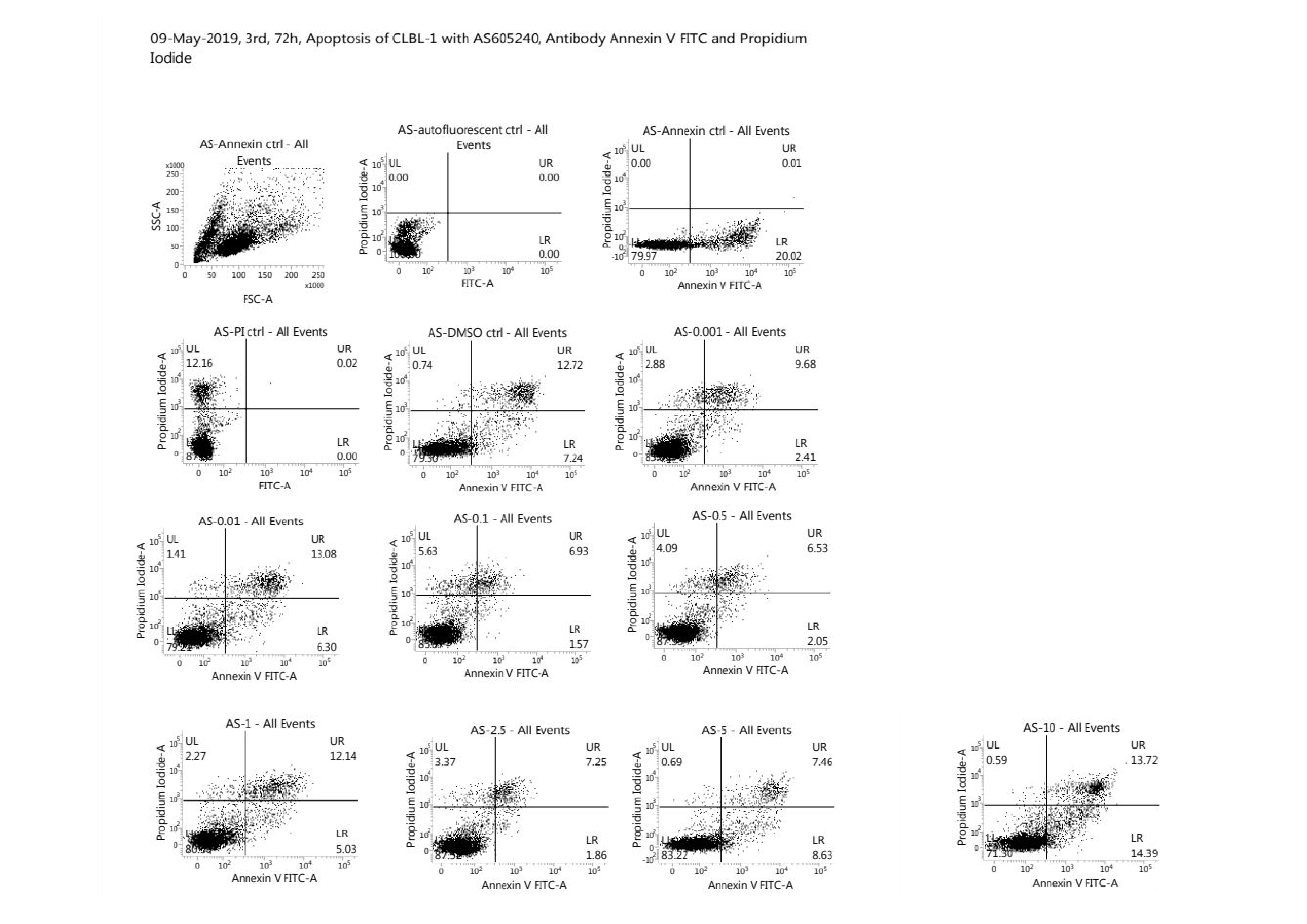

## Slide 26
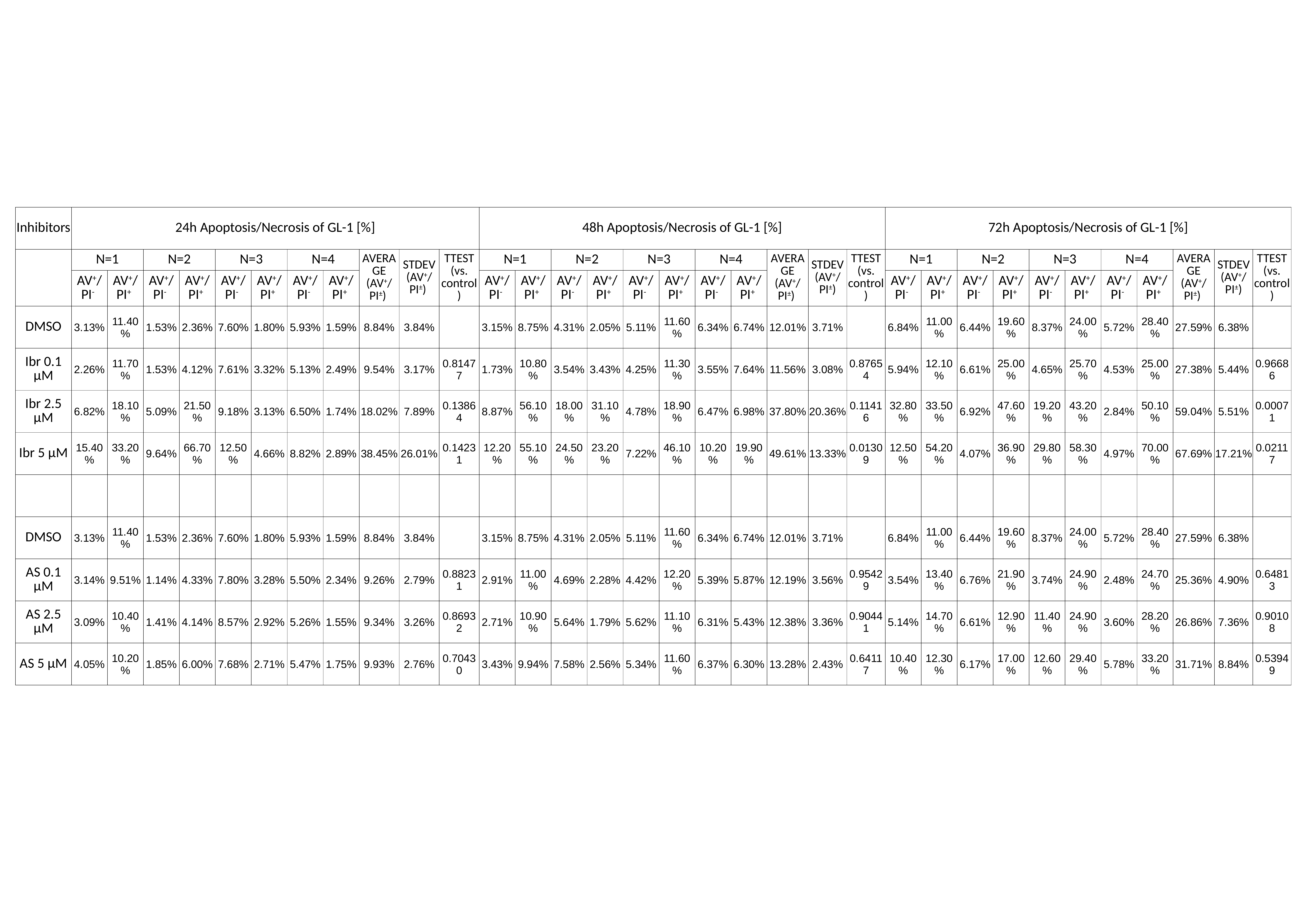

| Inhibitors | 24h Apoptosis/Necrosis of GL-1 [%] | | | | | | | | | | | 48h Apoptosis/Necrosis of GL-1 [%] | | | | | | | | | | | 72h Apoptosis/Necrosis of GL-1 [%] | | | | | | | | | | |
| --- | --- | --- | --- | --- | --- | --- | --- | --- | --- | --- | --- | --- | --- | --- | --- | --- | --- | --- | --- | --- | --- | --- | --- | --- | --- | --- | --- | --- | --- | --- | --- | --- | --- |
| | N=1 | | N=2 | | N=3 | | N=4 | | AVERAGE (AV+/ PI±) | STDEV (AV+/ PI±) | TTEST (vs. control) | N=1 | | N=2 | | N=3 | | N=4 | | AVERAGE (AV+/ PI±) | STDEV (AV+/ PI±) | TTEST (vs. control) | N=1 | | N=2 | | N=3 | | N=4 | | AVERAGE (AV+/ PI±) | STDEV (AV+/ PI±) | TTEST (vs. control) |
| | AV+/ PI- | AV+/ PI+ | AV+/ PI- | AV+/ PI+ | AV+/ PI- | AV+/ PI+ | AV+/ PI- | AV+/ PI+ | | | | AV+/ PI- | AV+/ PI+ | AV+/ PI- | AV+/ PI+ | AV+/ PI- | AV+/ PI+ | AV+/ PI- | AV+/ PI+ | | | | AV+/ PI- | AV+/ PI+ | AV+/ PI- | AV+/ PI+ | AV+/ PI- | AV+/ PI+ | AV+/ PI- | AV+/ PI+ | | | |
| DMSO | 3.13% | 11.40% | 1.53% | 2.36% | 7.60% | 1.80% | 5.93% | 1.59% | 8.84% | 3.84% | | 3.15% | 8.75% | 4.31% | 2.05% | 5.11% | 11.60% | 6.34% | 6.74% | 12.01% | 3.71% | | 6.84% | 11.00% | 6.44% | 19.60% | 8.37% | 24.00% | 5.72% | 28.40% | 27.59% | 6.38% | |
| Ibr 0.1 µM | 2.26% | 11.70% | 1.53% | 4.12% | 7.61% | 3.32% | 5.13% | 2.49% | 9.54% | 3.17% | 0.81477 | 1.73% | 10.80% | 3.54% | 3.43% | 4.25% | 11.30% | 3.55% | 7.64% | 11.56% | 3.08% | 0.87654 | 5.94% | 12.10% | 6.61% | 25.00% | 4.65% | 25.70% | 4.53% | 25.00% | 27.38% | 5.44% | 0.96686 |
| Ibr 2.5 µM | 6.82% | 18.10% | 5.09% | 21.50% | 9.18% | 3.13% | 6.50% | 1.74% | 18.02% | 7.89% | 0.13864 | 8.87% | 56.10% | 18.00% | 31.10% | 4.78% | 18.90% | 6.47% | 6.98% | 37.80% | 20.36% | 0.11416 | 32.80% | 33.50% | 6.92% | 47.60% | 19.20% | 43.20% | 2.84% | 50.10% | 59.04% | 5.51% | 0.00071 |
| Ibr 5 µM | 15.40% | 33.20% | 9.64% | 66.70% | 12.50% | 4.66% | 8.82% | 2.89% | 38.45% | 26.01% | 0.14231 | 12.20% | 55.10% | 24.50% | 23.20% | 7.22% | 46.10% | 10.20% | 19.90% | 49.61% | 13.33% | 0.01309 | 12.50% | 54.20% | 4.07% | 36.90% | 29.80% | 58.30% | 4.97% | 70.00% | 67.69% | 17.21% | 0.02117 |
| | | | | | | | | | | | | | | | | | | | | | | | | | | | | | | | | | |
| DMSO | 3.13% | 11.40% | 1.53% | 2.36% | 7.60% | 1.80% | 5.93% | 1.59% | 8.84% | 3.84% | | 3.15% | 8.75% | 4.31% | 2.05% | 5.11% | 11.60% | 6.34% | 6.74% | 12.01% | 3.71% | | 6.84% | 11.00% | 6.44% | 19.60% | 8.37% | 24.00% | 5.72% | 28.40% | 27.59% | 6.38% | |
| AS 0.1 µM | 3.14% | 9.51% | 1.14% | 4.33% | 7.80% | 3.28% | 5.50% | 2.34% | 9.26% | 2.79% | 0.88231 | 2.91% | 11.00% | 4.69% | 2.28% | 4.42% | 12.20% | 5.39% | 5.87% | 12.19% | 3.56% | 0.95429 | 3.54% | 13.40% | 6.76% | 21.90% | 3.74% | 24.90% | 2.48% | 24.70% | 25.36% | 4.90% | 0.64813 |
| AS 2.5 µM | 3.09% | 10.40% | 1.41% | 4.14% | 8.57% | 2.92% | 5.26% | 1.55% | 9.34% | 3.26% | 0.86932 | 2.71% | 10.90% | 5.64% | 1.79% | 5.62% | 11.10% | 6.31% | 5.43% | 12.38% | 3.36% | 0.90441 | 5.14% | 14.70% | 6.61% | 12.90% | 11.40% | 24.90% | 3.60% | 28.20% | 26.86% | 7.36% | 0.90108 |
| AS 5 µM | 4.05% | 10.20% | 1.85% | 6.00% | 7.68% | 2.71% | 5.47% | 1.75% | 9.93% | 2.76% | 0.70430 | 3.43% | 9.94% | 7.58% | 2.56% | 5.34% | 11.60% | 6.37% | 6.30% | 13.28% | 2.43% | 0.64117 | 10.40% | 12.30% | 6.17% | 17.00% | 12.60% | 29.40% | 5.78% | 33.20% | 31.71% | 8.84% | 0.53949 |

## Slide 27
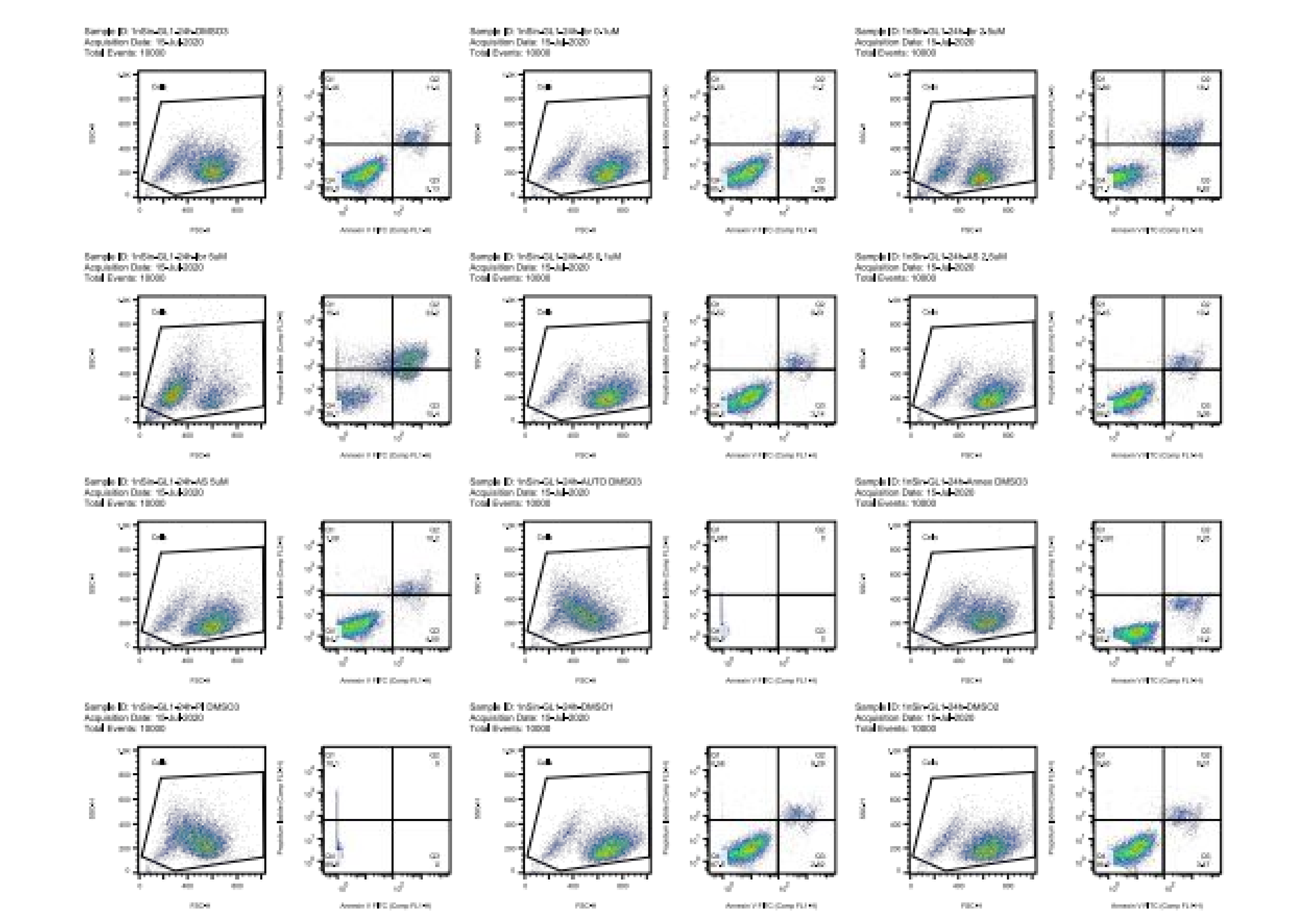

## Slide 28
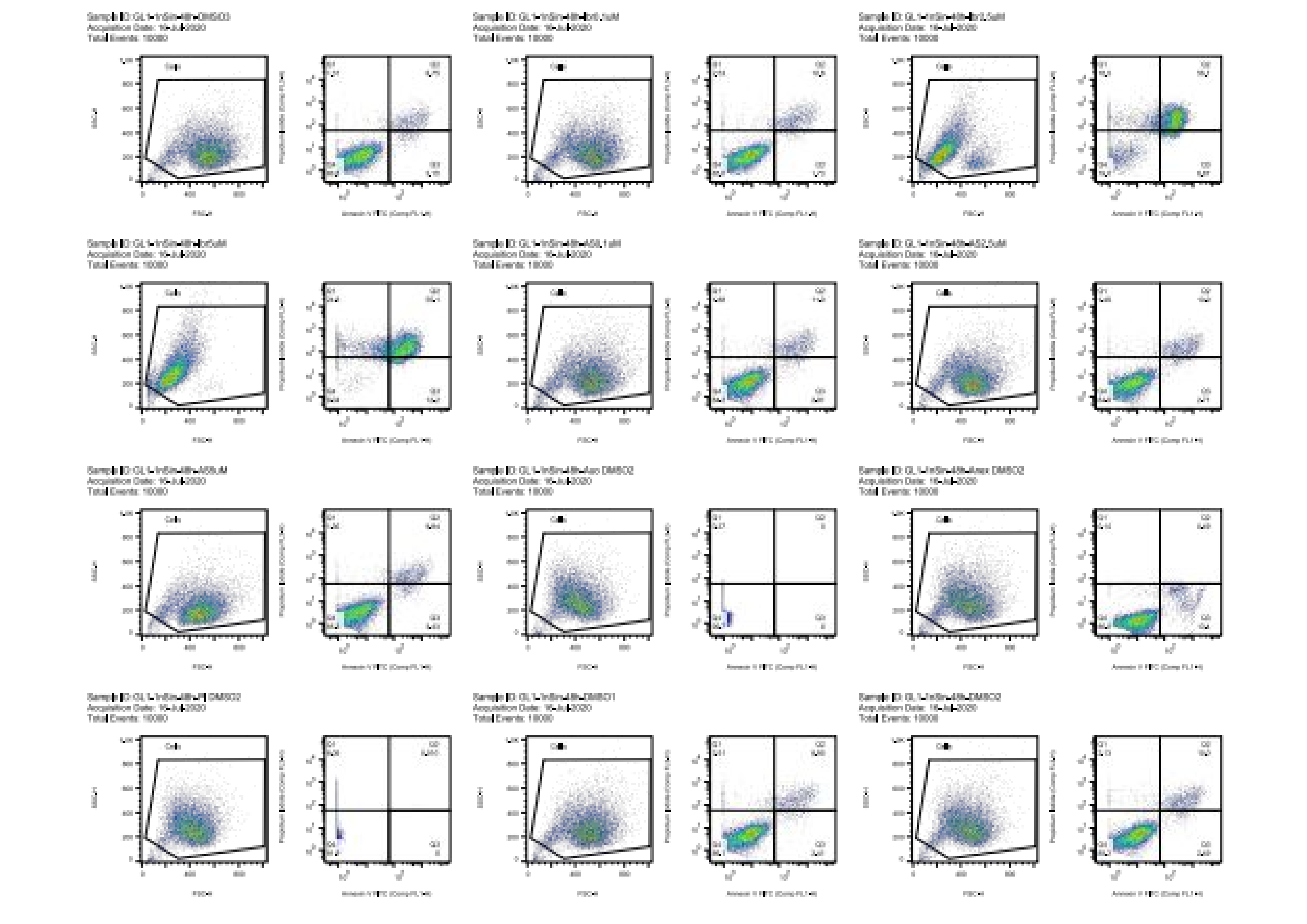

## Slide 29
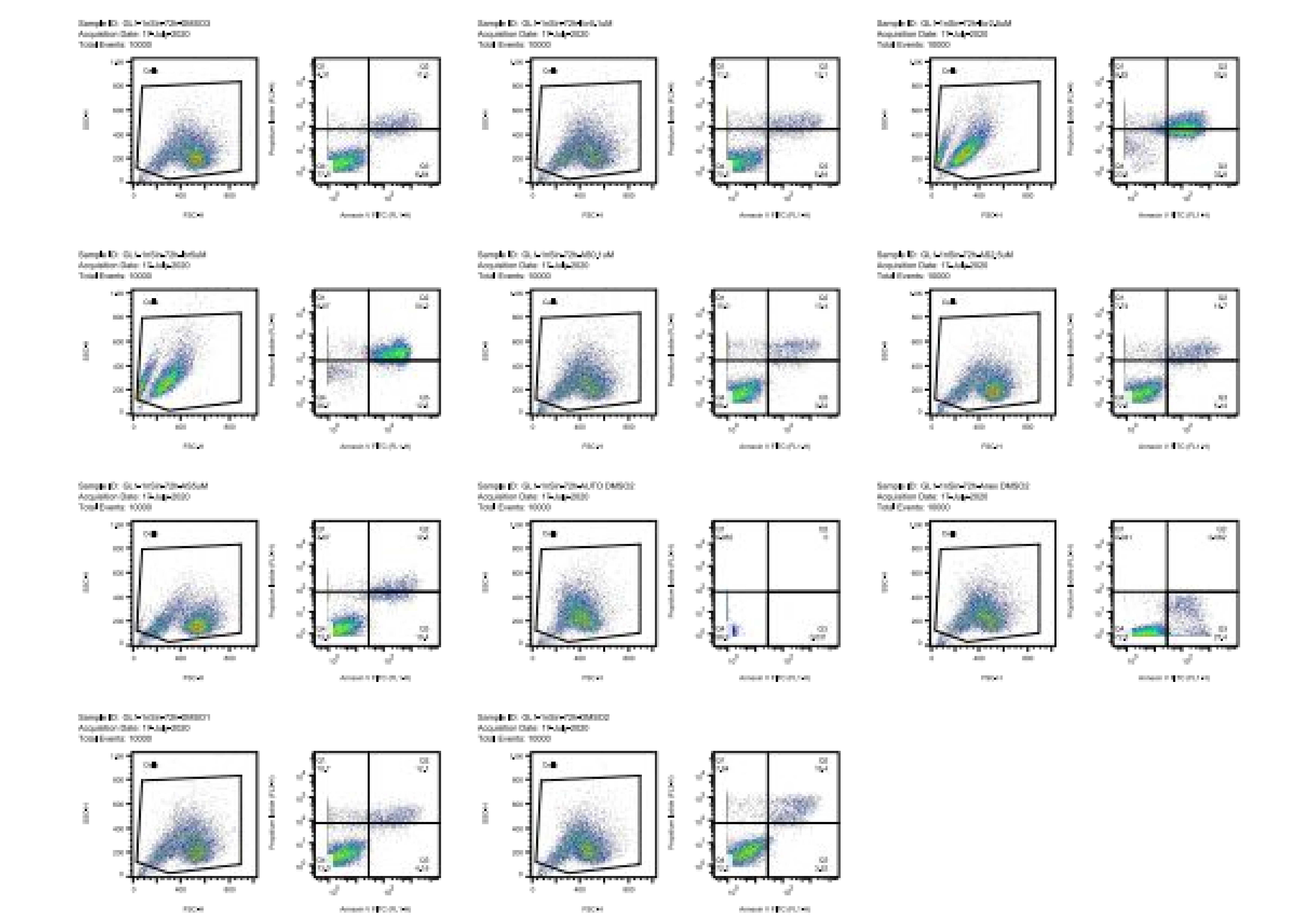

## Slide 30
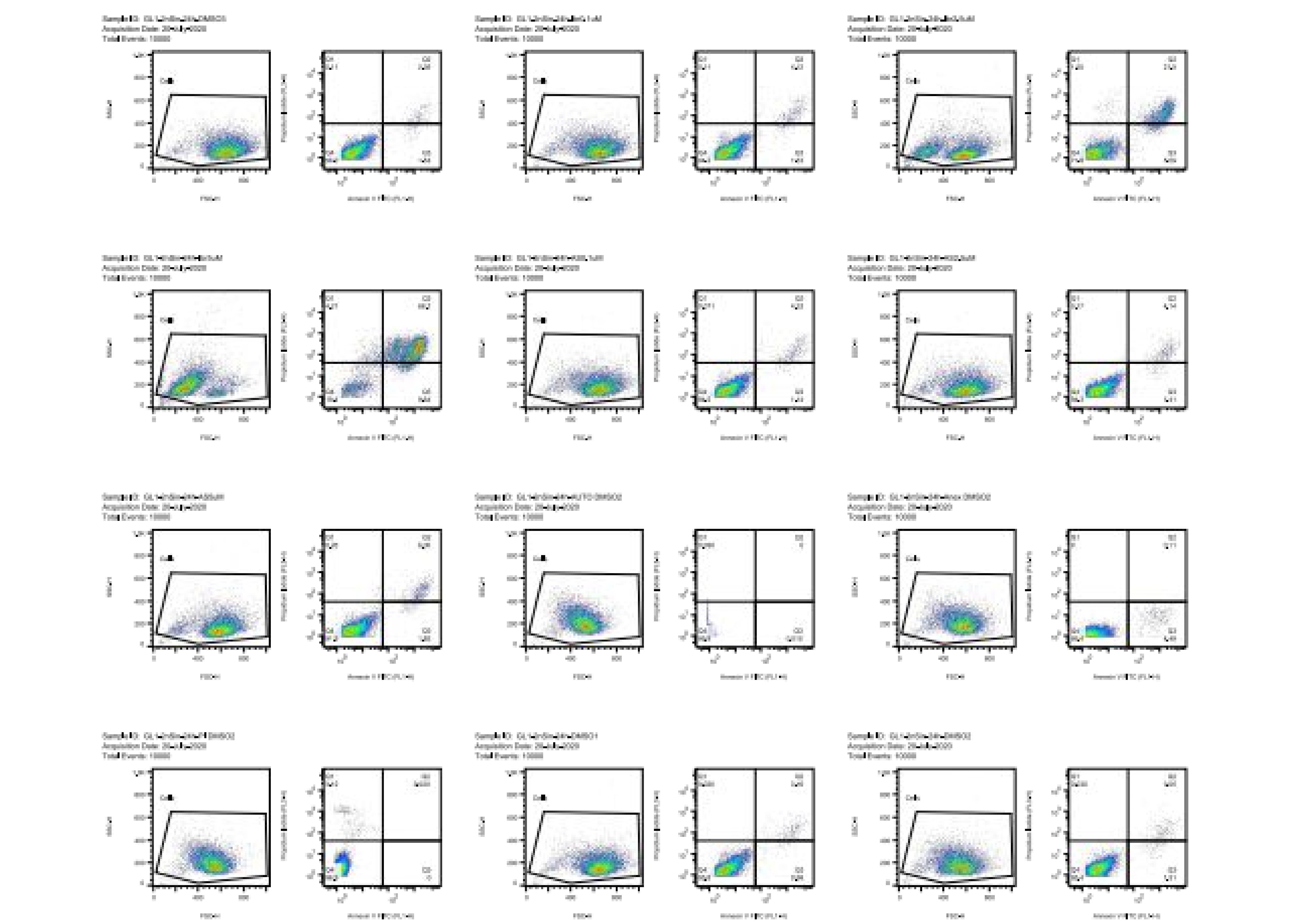

## Slide 31
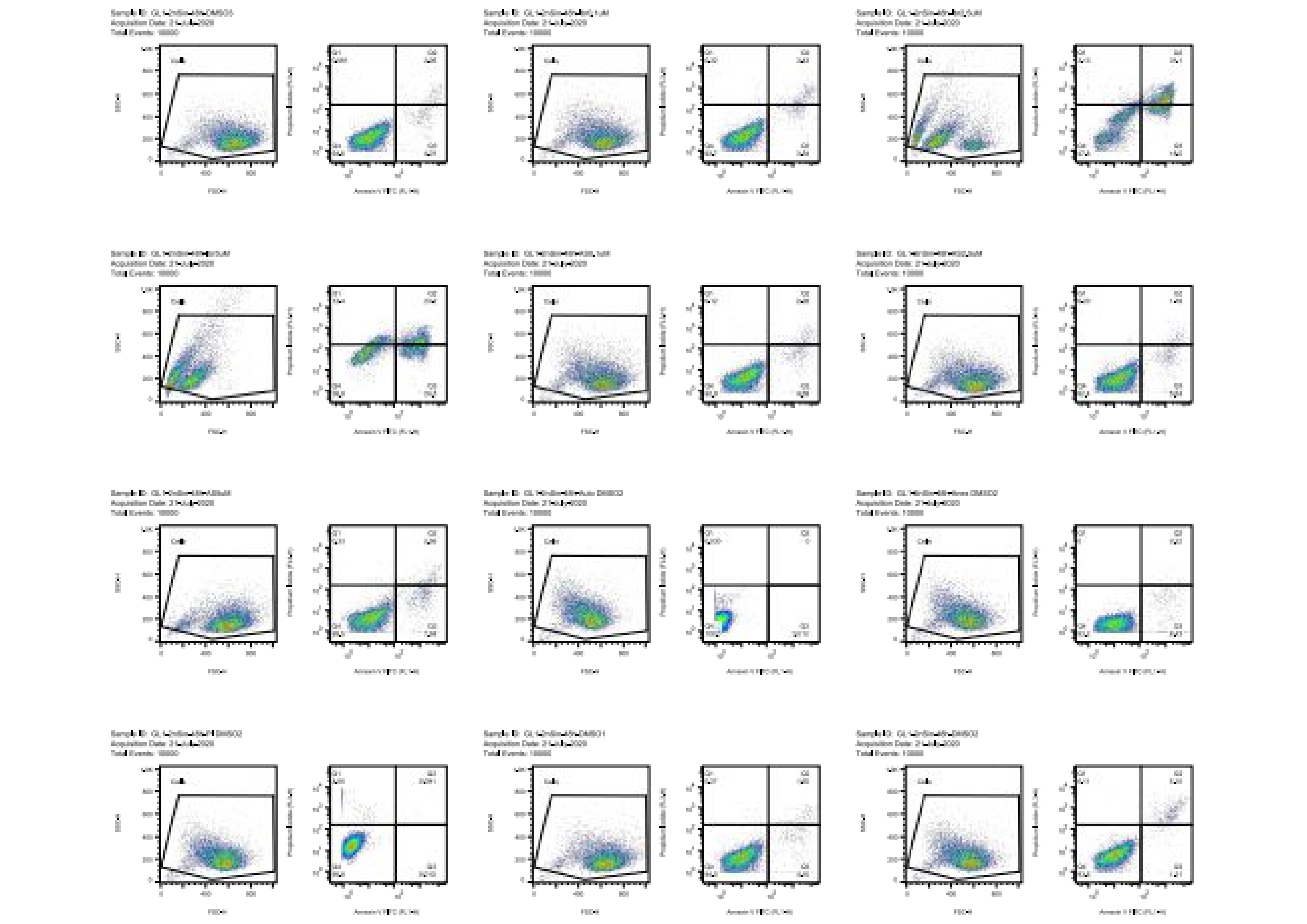

## Slide 32
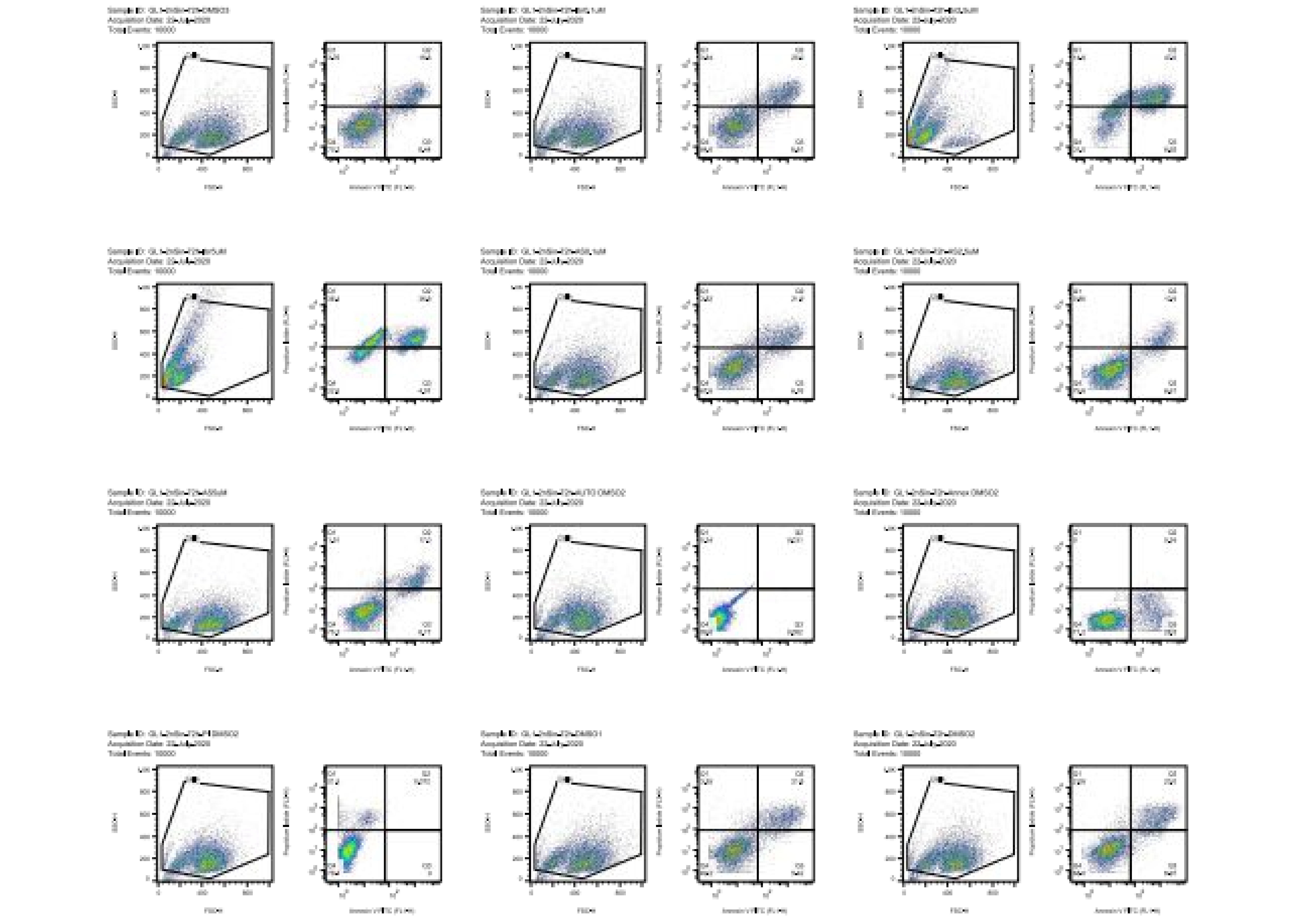

## Slide 33
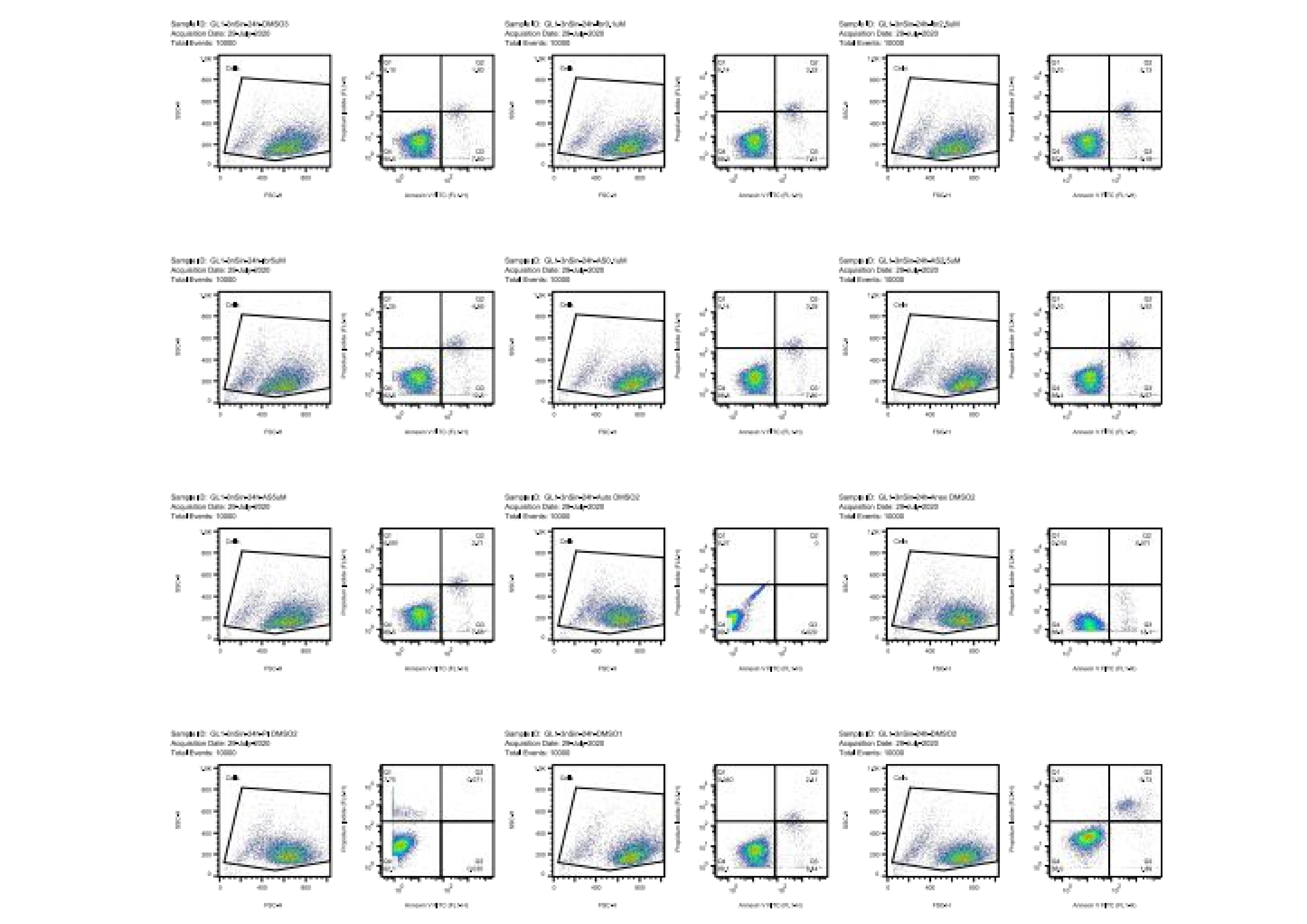

## Slide 34
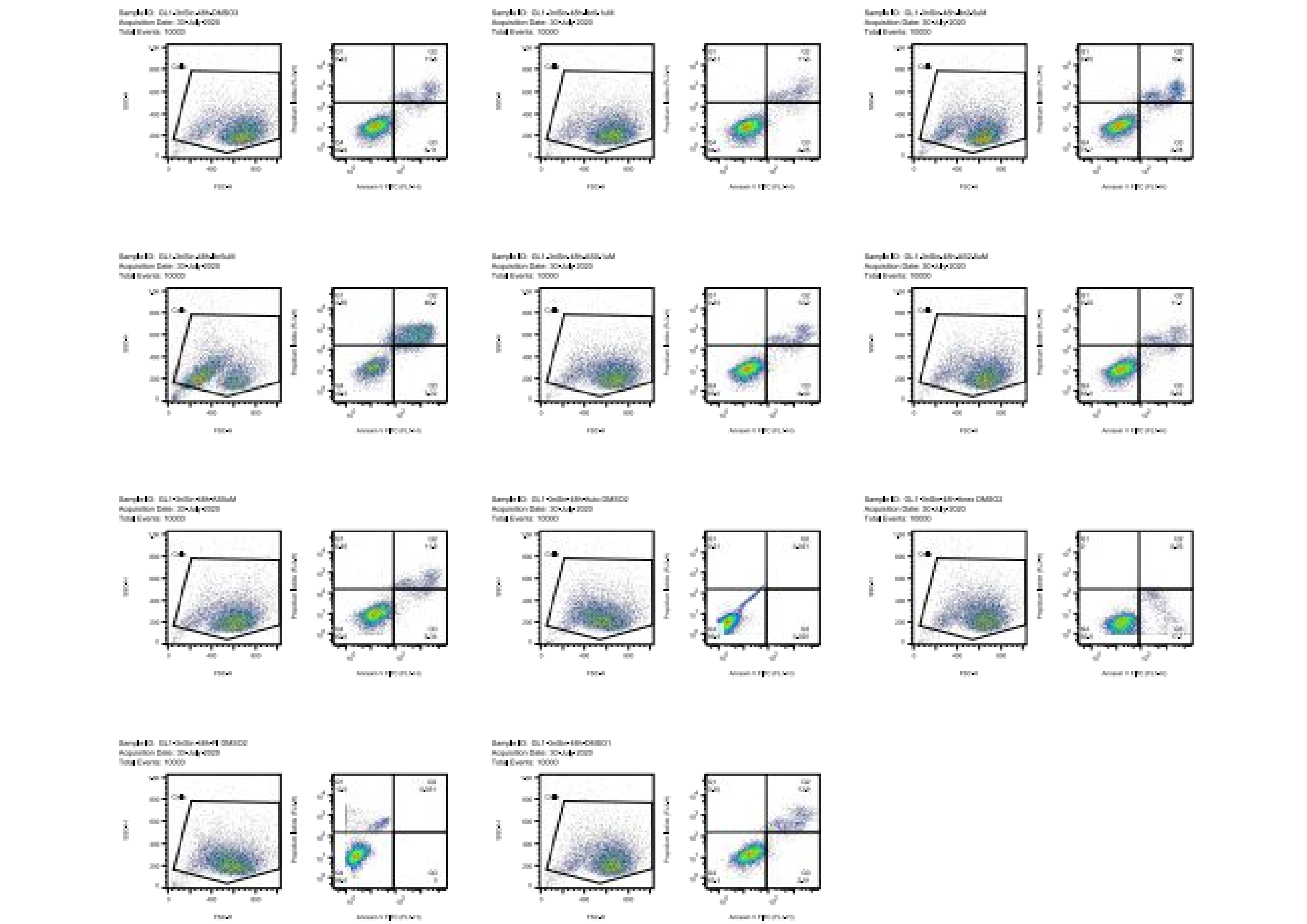

## Slide 35
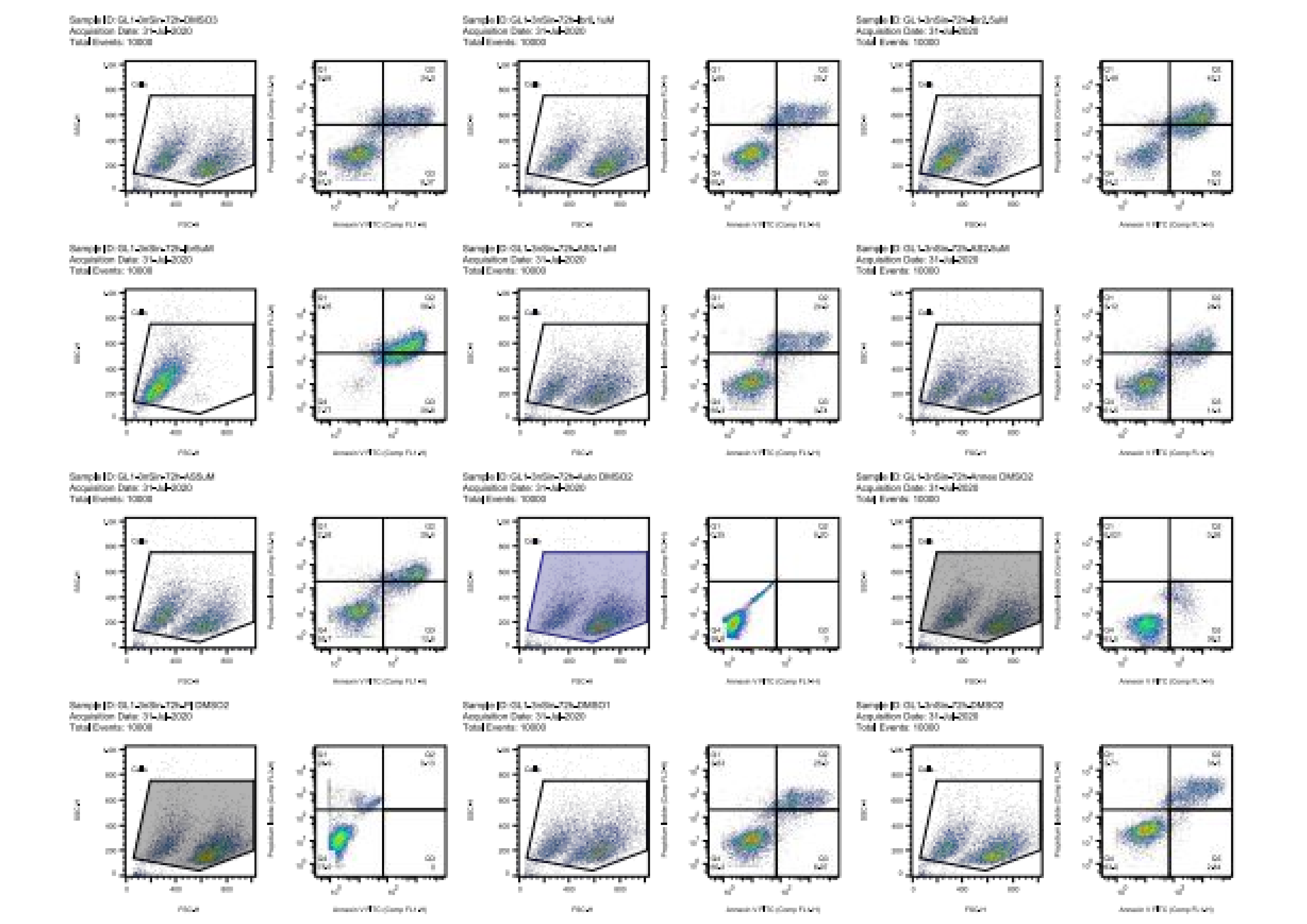

## Slide 36
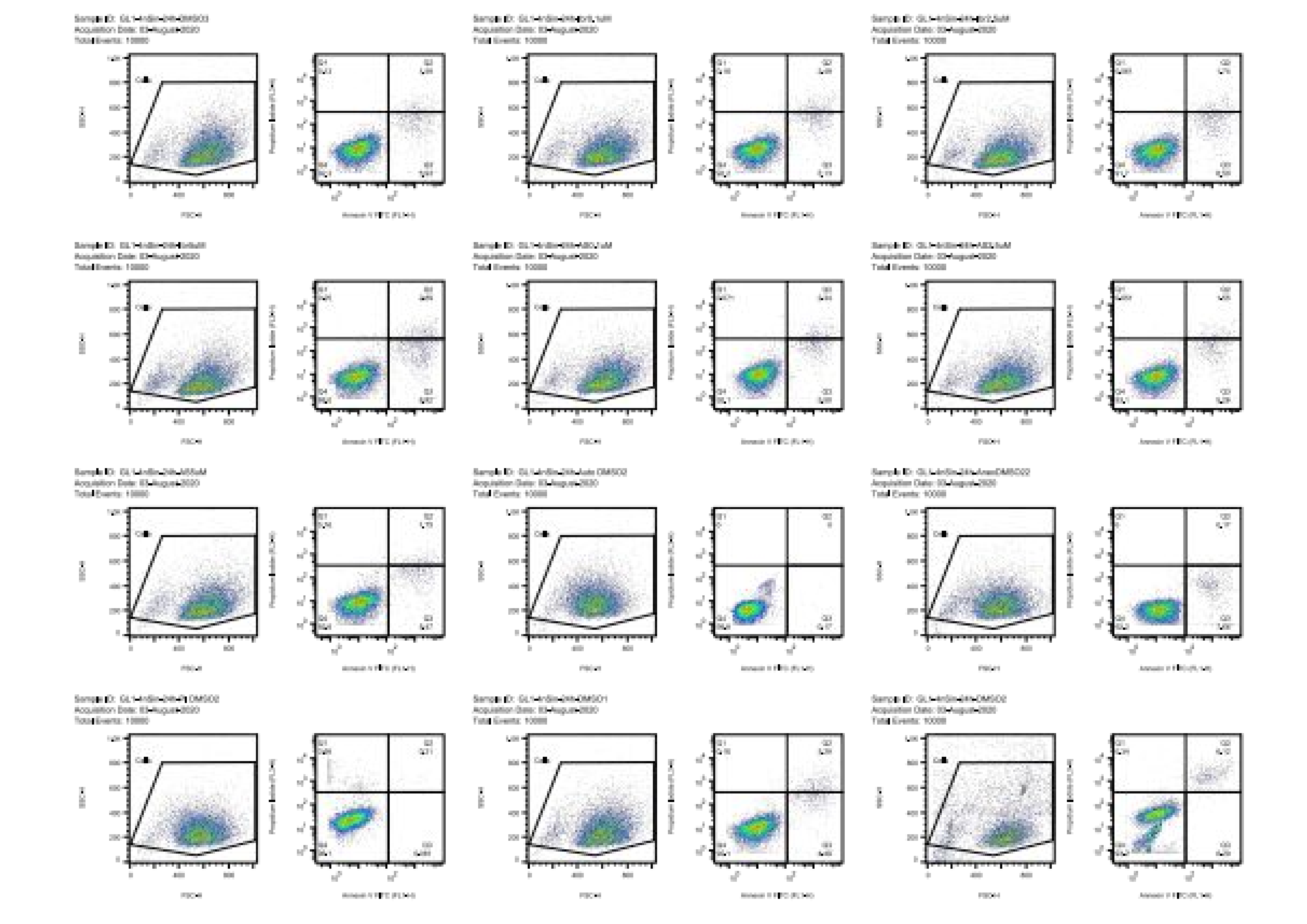

## Slide 37
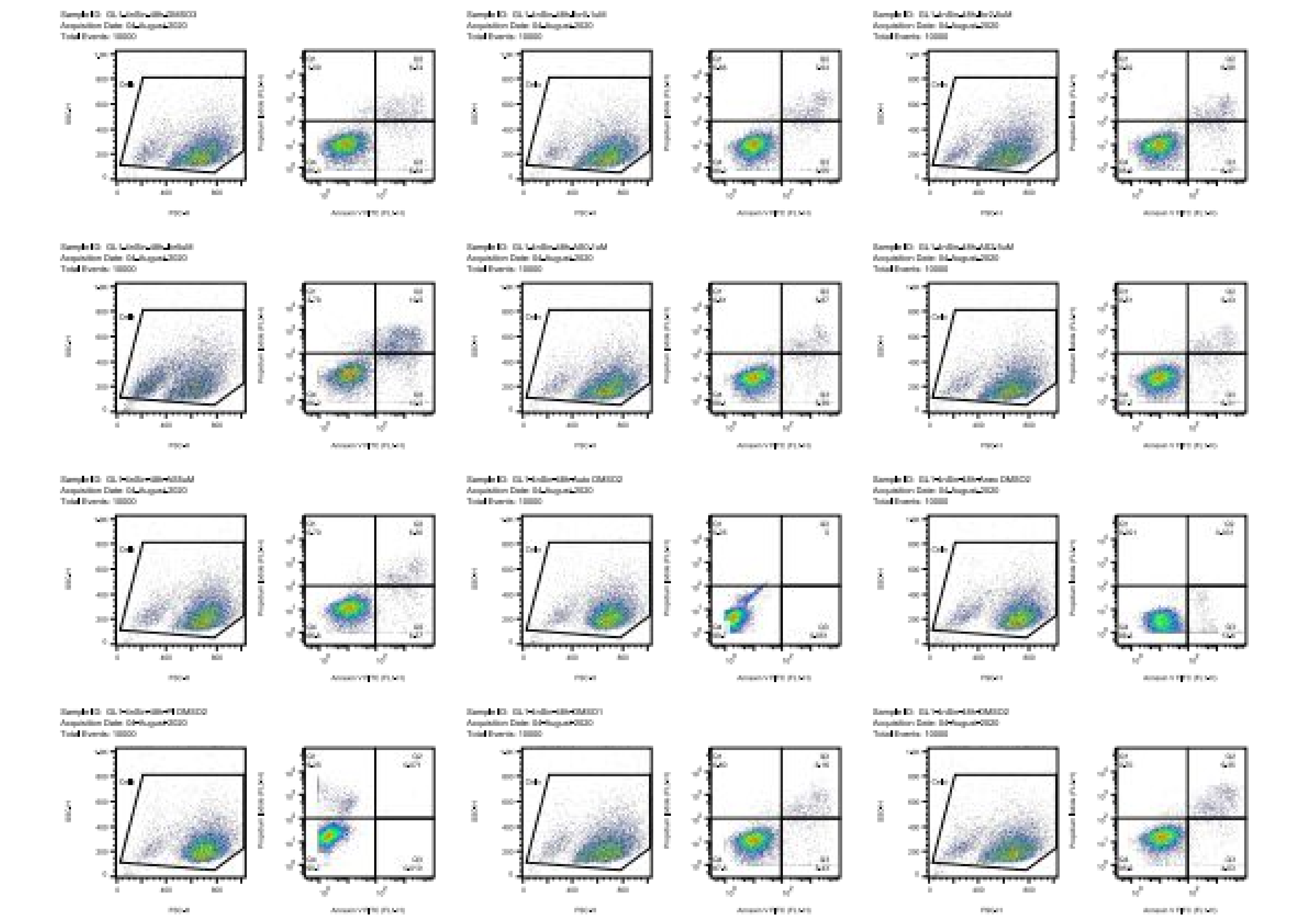

## Slide 38
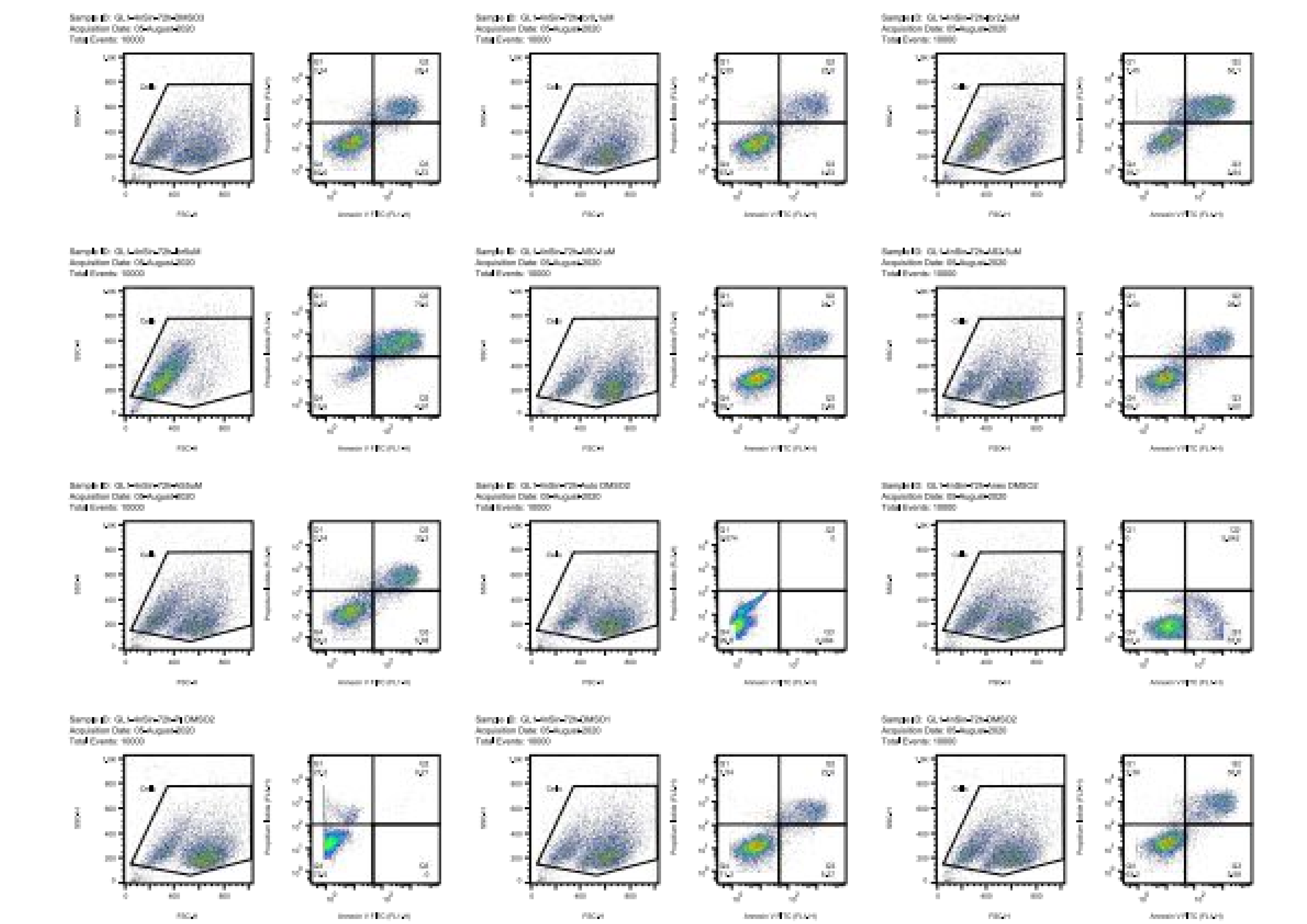

## Slide 39
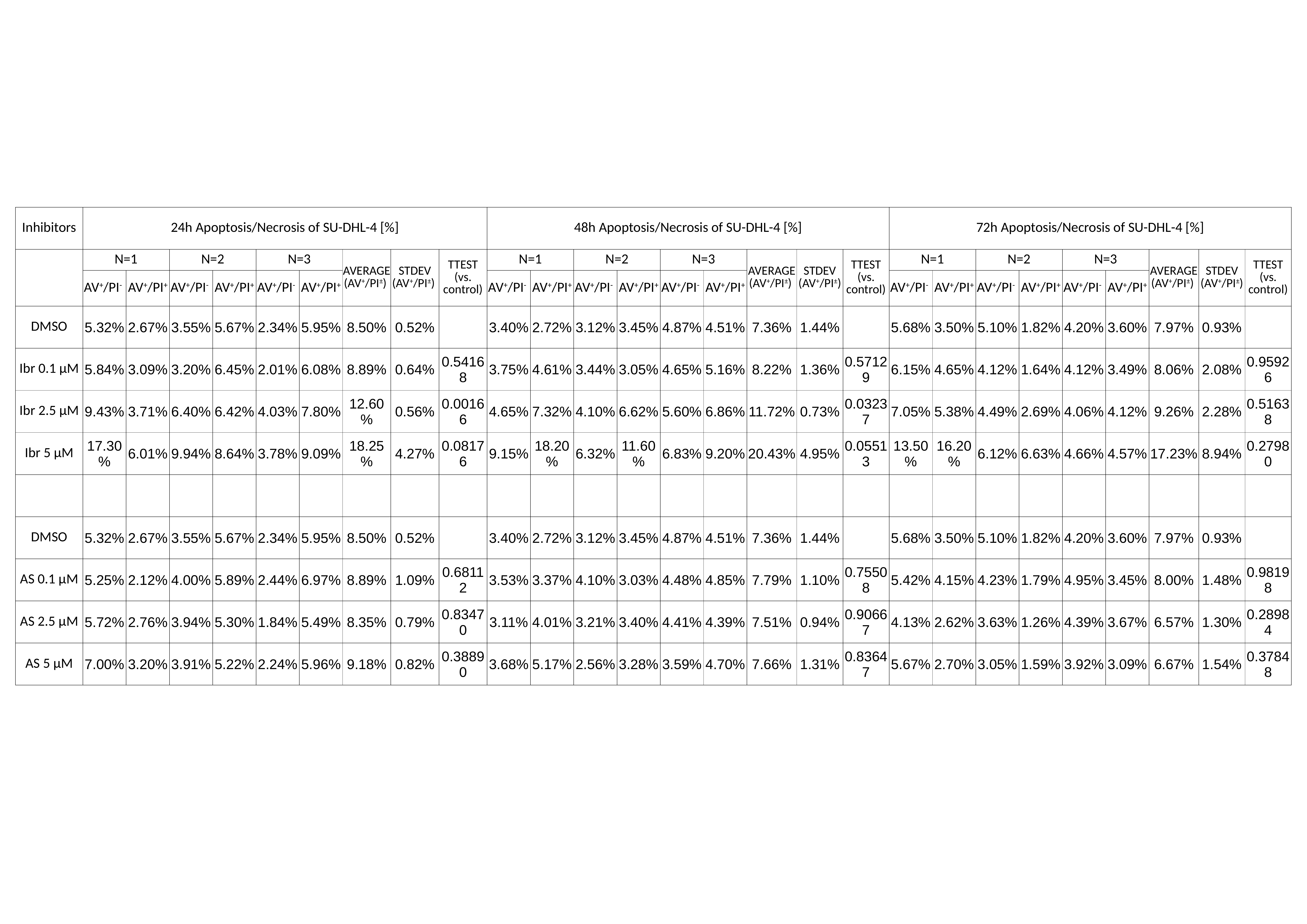

| Inhibitors | 24h Apoptosis/Necrosis of SU-DHL-4 [%] | | | | | | | | | 48h Apoptosis/Necrosis of SU-DHL-4 [%] | | | | | | | | | 72h Apoptosis/Necrosis of SU-DHL-4 [%] | | | | | | | | |
| --- | --- | --- | --- | --- | --- | --- | --- | --- | --- | --- | --- | --- | --- | --- | --- | --- | --- | --- | --- | --- | --- | --- | --- | --- | --- | --- | --- |
| | N=1 | | N=2 | | N=3 | | AVERAGE (AV+/PI±) | STDEV (AV+/PI±) | TTEST (vs. control) | N=1 | | N=2 | | N=3 | | AVERAGE (AV+/PI±) | STDEV (AV+/PI±) | TTEST (vs. control) | N=1 | | N=2 | | N=3 | | AVERAGE (AV+/PI±) | STDEV (AV+/PI±) | TTEST (vs. control) |
| | AV+/PI- | AV+/PI+ | AV+/PI- | AV+/PI+ | AV+/PI- | AV+/PI+ | | | | AV+/PI- | AV+/PI+ | AV+/PI- | AV+/PI+ | AV+/PI- | AV+/PI+ | | | | AV+/PI- | AV+/PI+ | AV+/PI- | AV+/PI+ | AV+/PI- | AV+/PI+ | | | |
| DMSO | 5.32% | 2.67% | 3.55% | 5.67% | 2.34% | 5.95% | 8.50% | 0.52% | | 3.40% | 2.72% | 3.12% | 3.45% | 4.87% | 4.51% | 7.36% | 1.44% | | 5.68% | 3.50% | 5.10% | 1.82% | 4.20% | 3.60% | 7.97% | 0.93% | |
| Ibr 0.1 µM | 5.84% | 3.09% | 3.20% | 6.45% | 2.01% | 6.08% | 8.89% | 0.64% | 0.54168 | 3.75% | 4.61% | 3.44% | 3.05% | 4.65% | 5.16% | 8.22% | 1.36% | 0.57129 | 6.15% | 4.65% | 4.12% | 1.64% | 4.12% | 3.49% | 8.06% | 2.08% | 0.95926 |
| Ibr 2.5 µM | 9.43% | 3.71% | 6.40% | 6.42% | 4.03% | 7.80% | 12.60% | 0.56% | 0.00166 | 4.65% | 7.32% | 4.10% | 6.62% | 5.60% | 6.86% | 11.72% | 0.73% | 0.03237 | 7.05% | 5.38% | 4.49% | 2.69% | 4.06% | 4.12% | 9.26% | 2.28% | 0.51638 |
| Ibr 5 µM | 17.30% | 6.01% | 9.94% | 8.64% | 3.78% | 9.09% | 18.25% | 4.27% | 0.08176 | 9.15% | 18.20% | 6.32% | 11.60% | 6.83% | 9.20% | 20.43% | 4.95% | 0.05513 | 13.50% | 16.20% | 6.12% | 6.63% | 4.66% | 4.57% | 17.23% | 8.94% | 0.27980 |
| | | | | | | | | | | | | | | | | | | | | | | | | | | | |
| DMSO | 5.32% | 2.67% | 3.55% | 5.67% | 2.34% | 5.95% | 8.50% | 0.52% | | 3.40% | 2.72% | 3.12% | 3.45% | 4.87% | 4.51% | 7.36% | 1.44% | | 5.68% | 3.50% | 5.10% | 1.82% | 4.20% | 3.60% | 7.97% | 0.93% | |
| AS 0.1 µM | 5.25% | 2.12% | 4.00% | 5.89% | 2.44% | 6.97% | 8.89% | 1.09% | 0.68112 | 3.53% | 3.37% | 4.10% | 3.03% | 4.48% | 4.85% | 7.79% | 1.10% | 0.75508 | 5.42% | 4.15% | 4.23% | 1.79% | 4.95% | 3.45% | 8.00% | 1.48% | 0.98198 |
| AS 2.5 µM | 5.72% | 2.76% | 3.94% | 5.30% | 1.84% | 5.49% | 8.35% | 0.79% | 0.83470 | 3.11% | 4.01% | 3.21% | 3.40% | 4.41% | 4.39% | 7.51% | 0.94% | 0.90667 | 4.13% | 2.62% | 3.63% | 1.26% | 4.39% | 3.67% | 6.57% | 1.30% | 0.28984 |
| AS 5 µM | 7.00% | 3.20% | 3.91% | 5.22% | 2.24% | 5.96% | 9.18% | 0.82% | 0.38890 | 3.68% | 5.17% | 2.56% | 3.28% | 3.59% | 4.70% | 7.66% | 1.31% | 0.83647 | 5.67% | 2.70% | 3.05% | 1.59% | 3.92% | 3.09% | 6.67% | 1.54% | 0.37848 |

## Slide 40
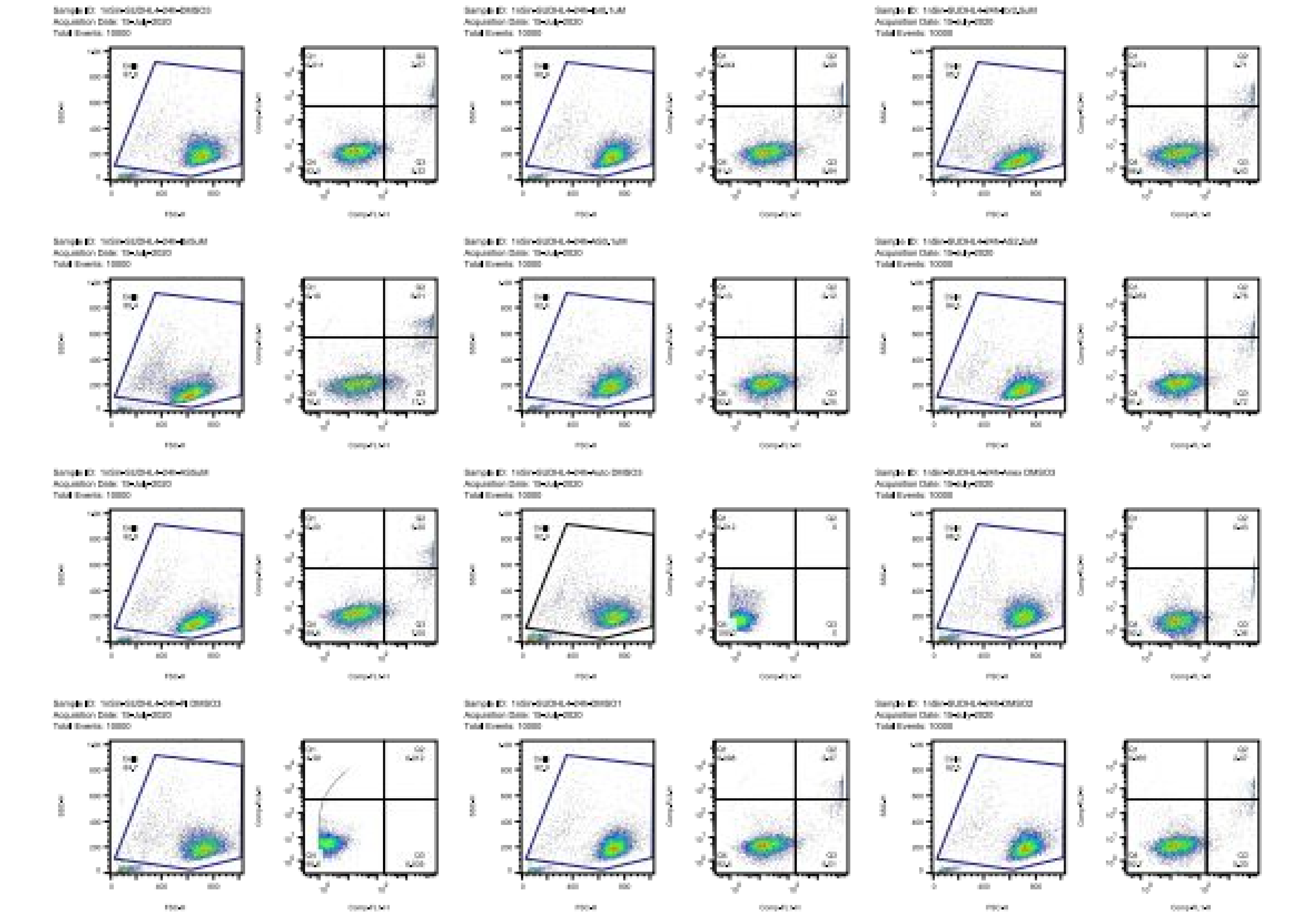

## Slide 41
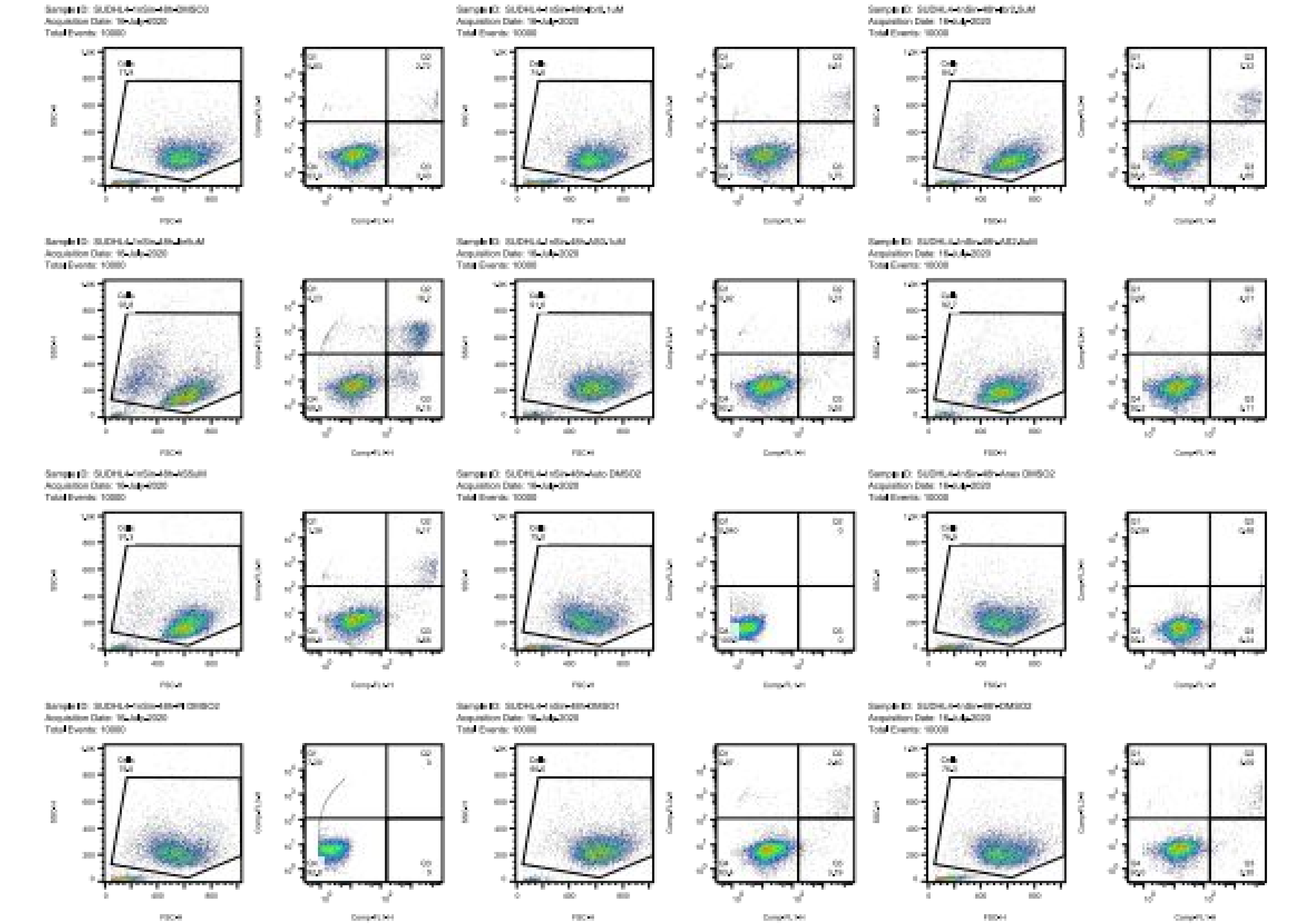

## Slide 42
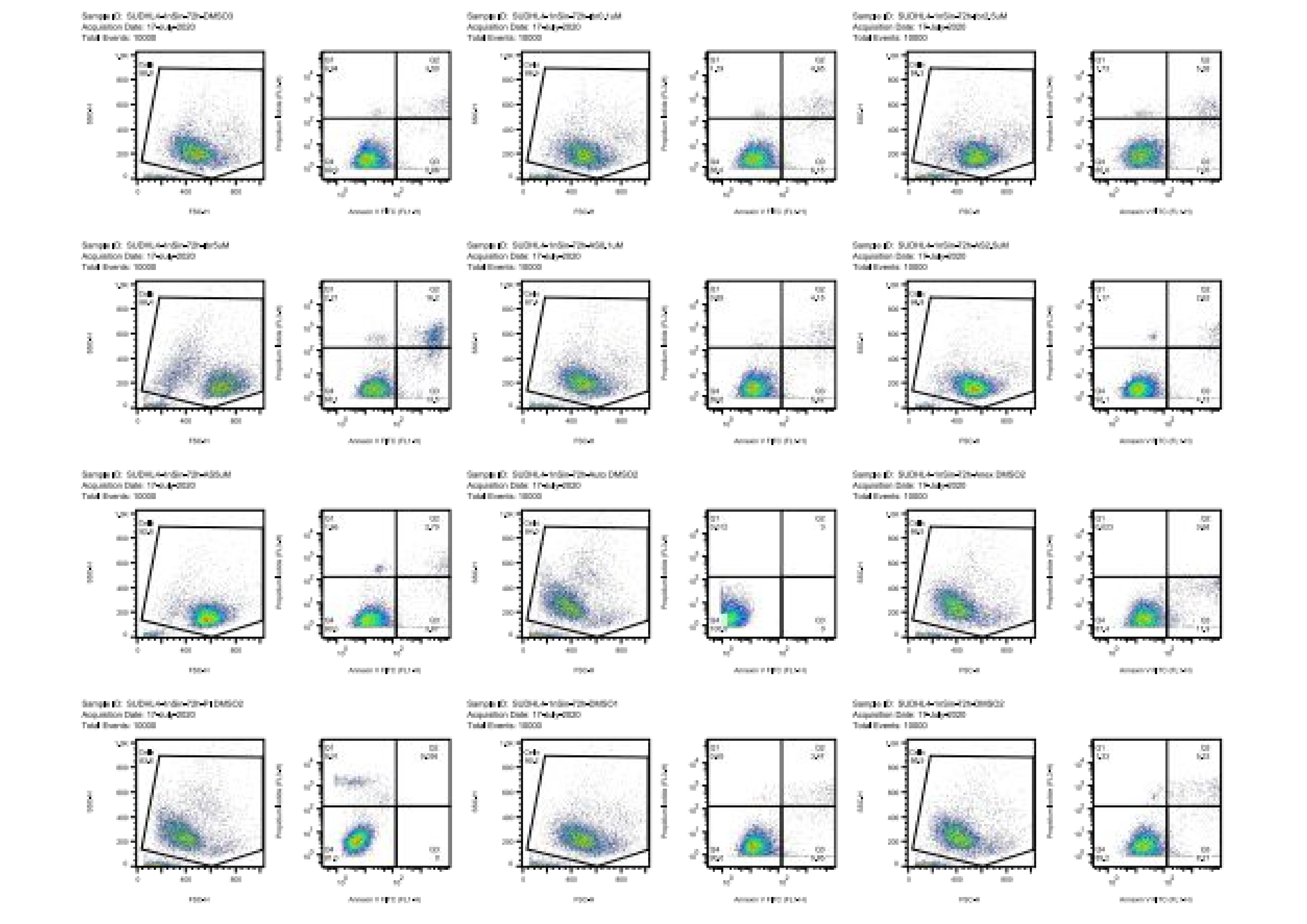

## Slide 43
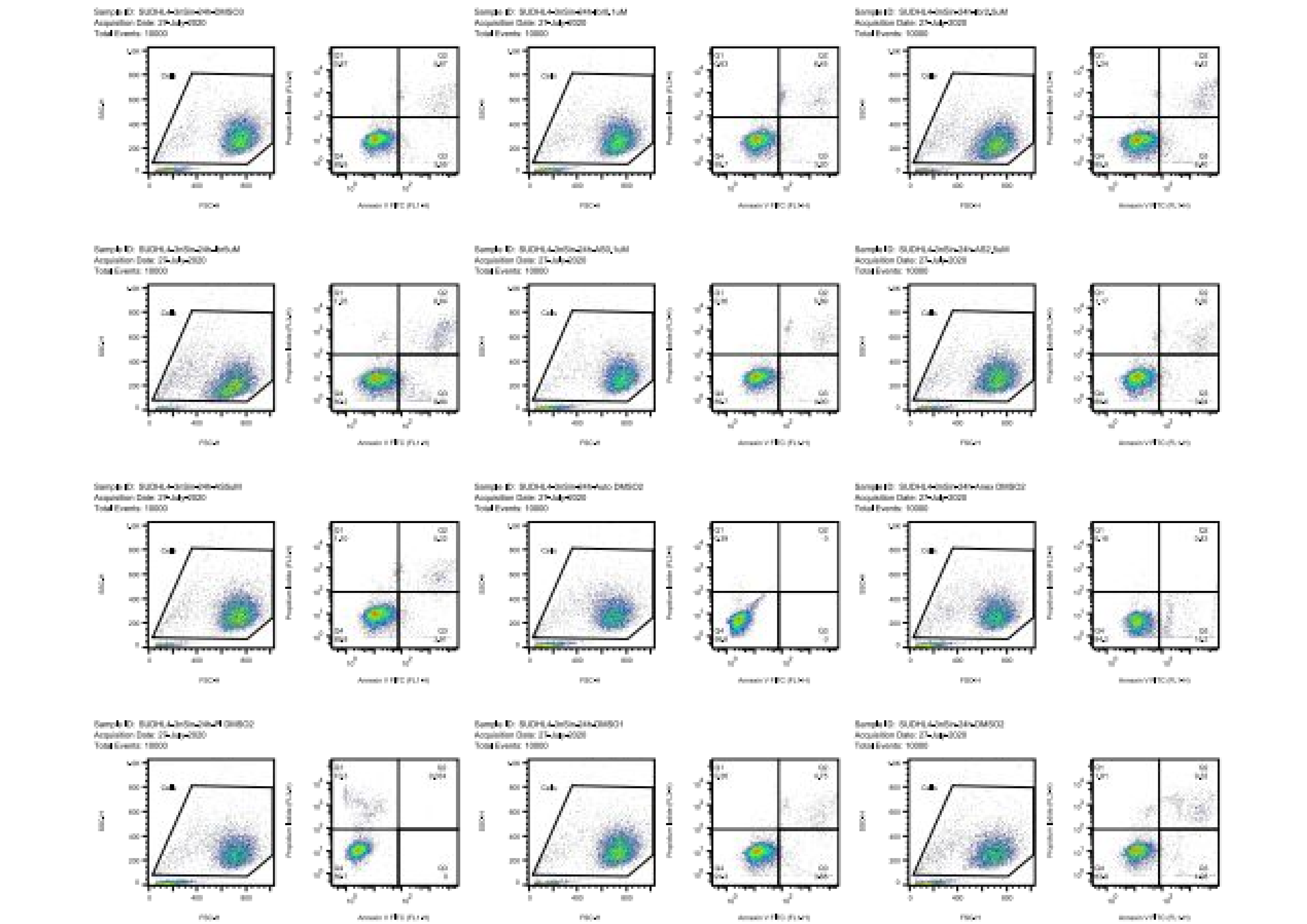

## Slide 44
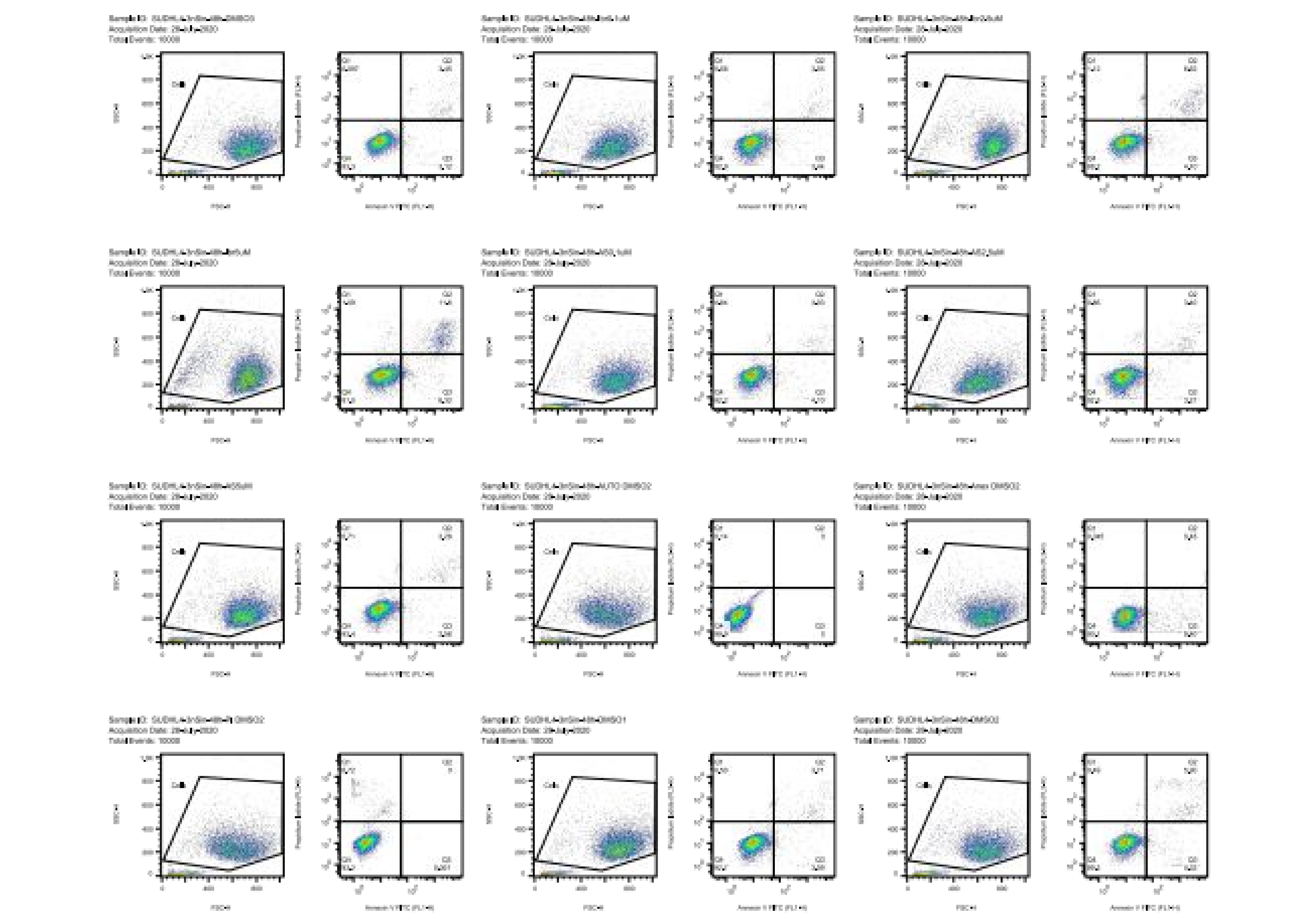

## Slide 45
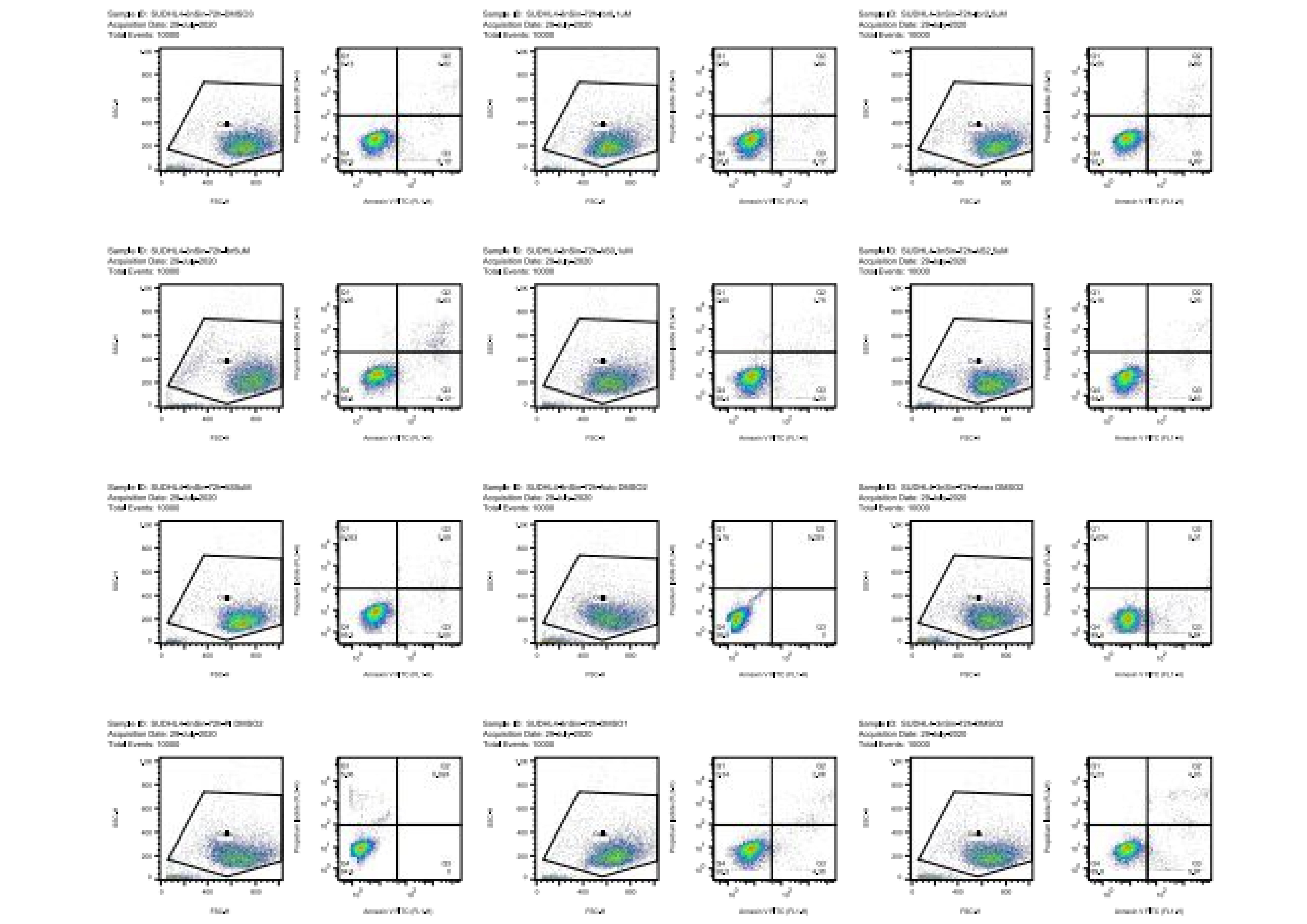

## Slide 46
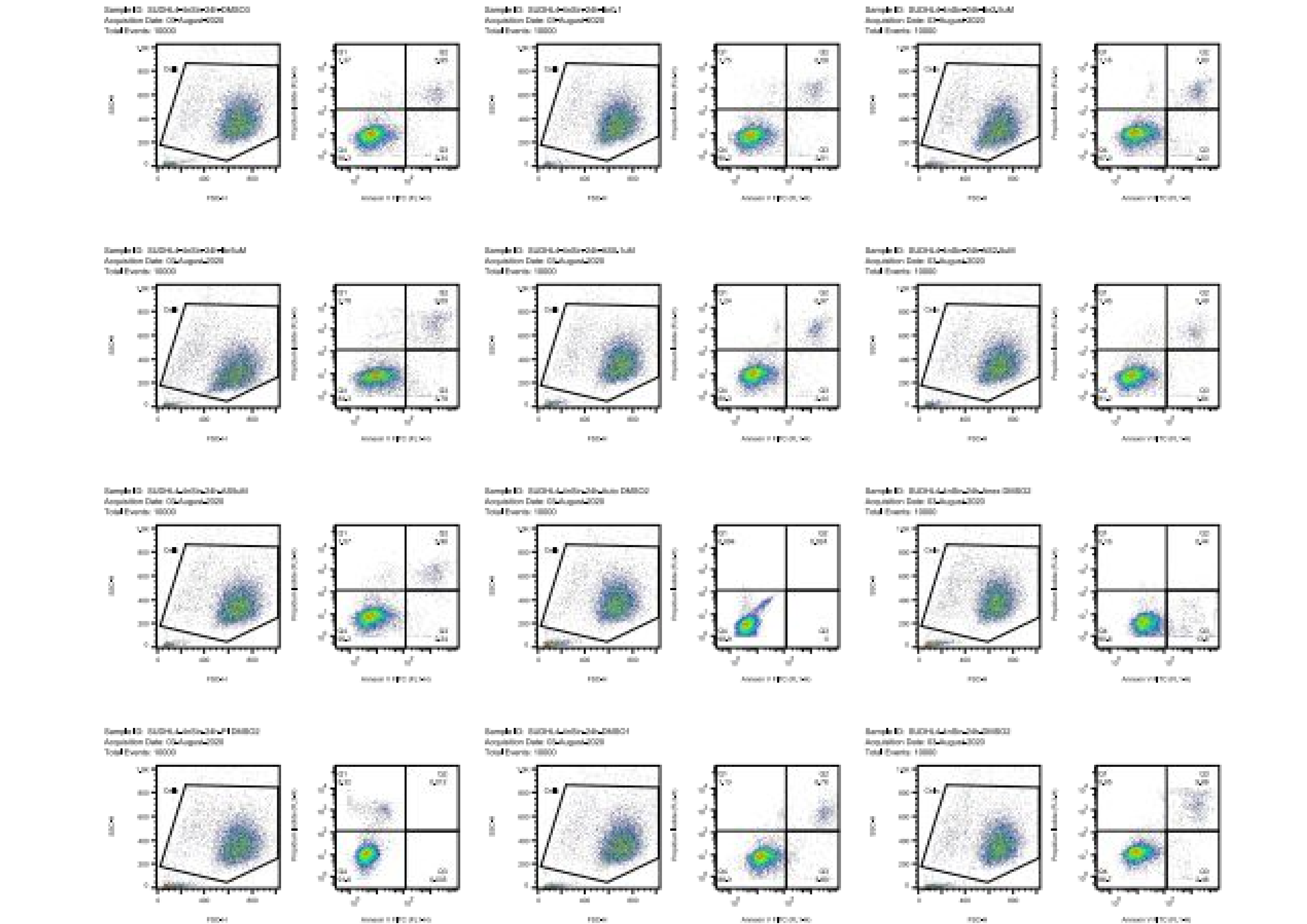

## Slide 47
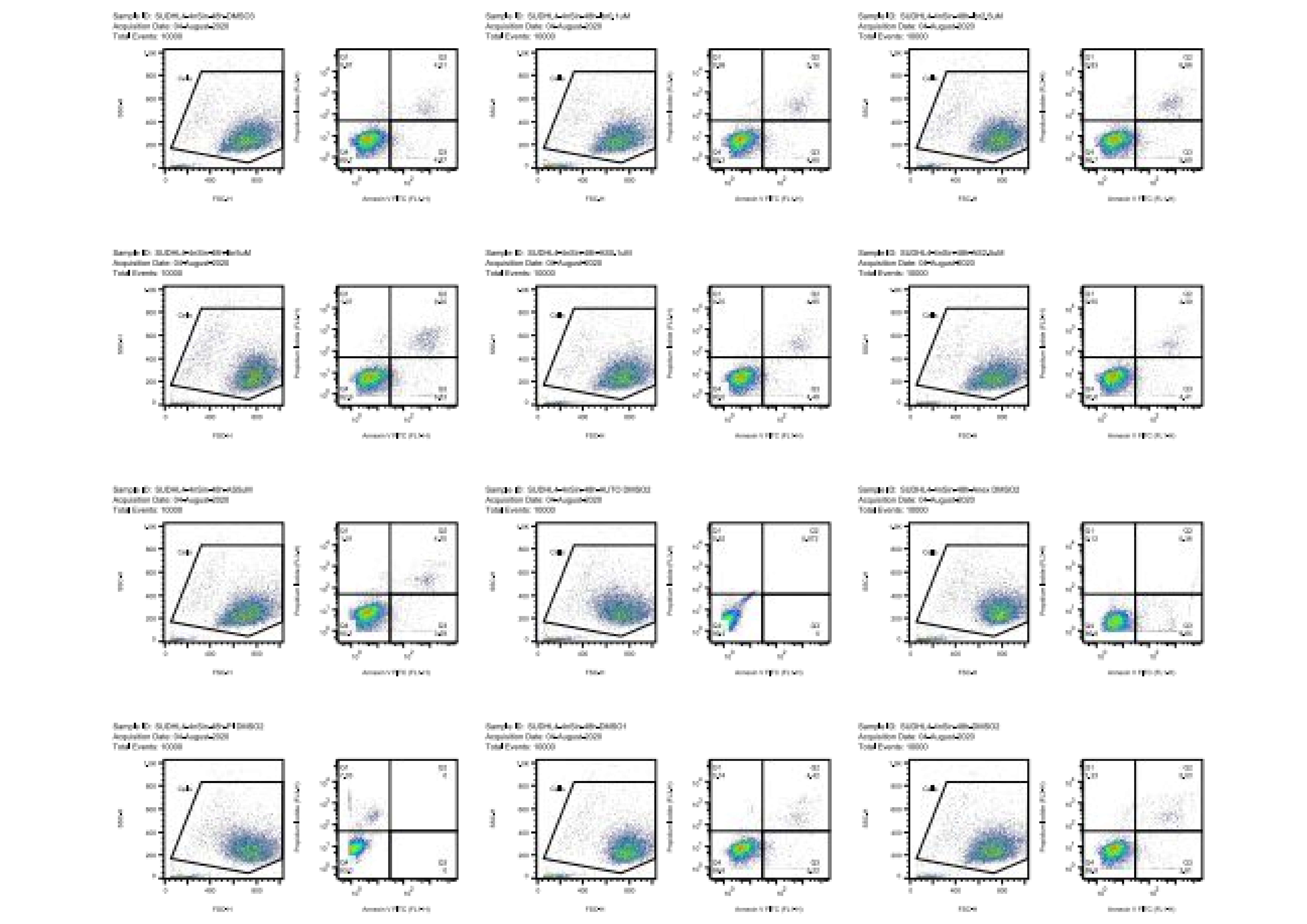

## Slide 48
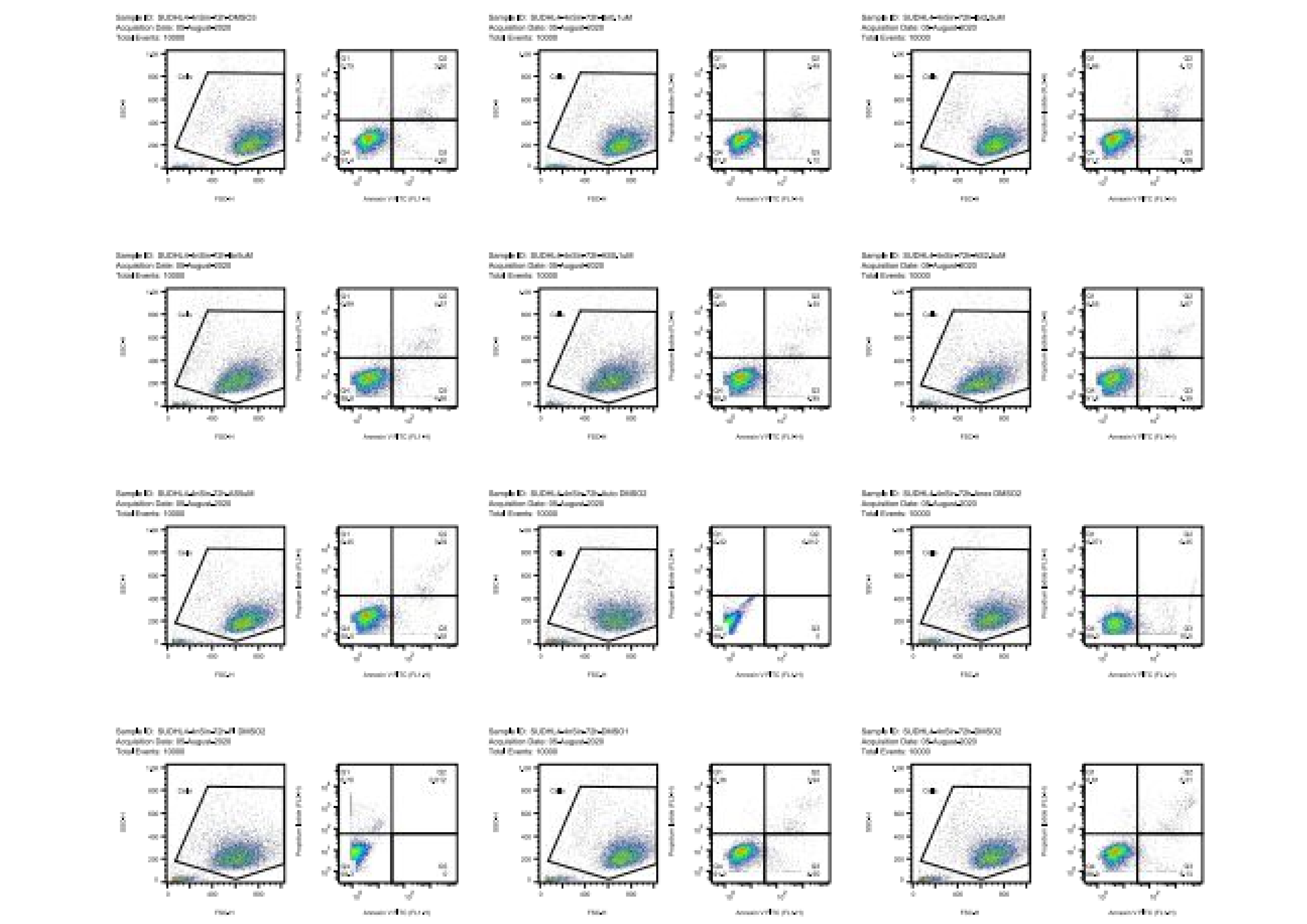

## Slide 49
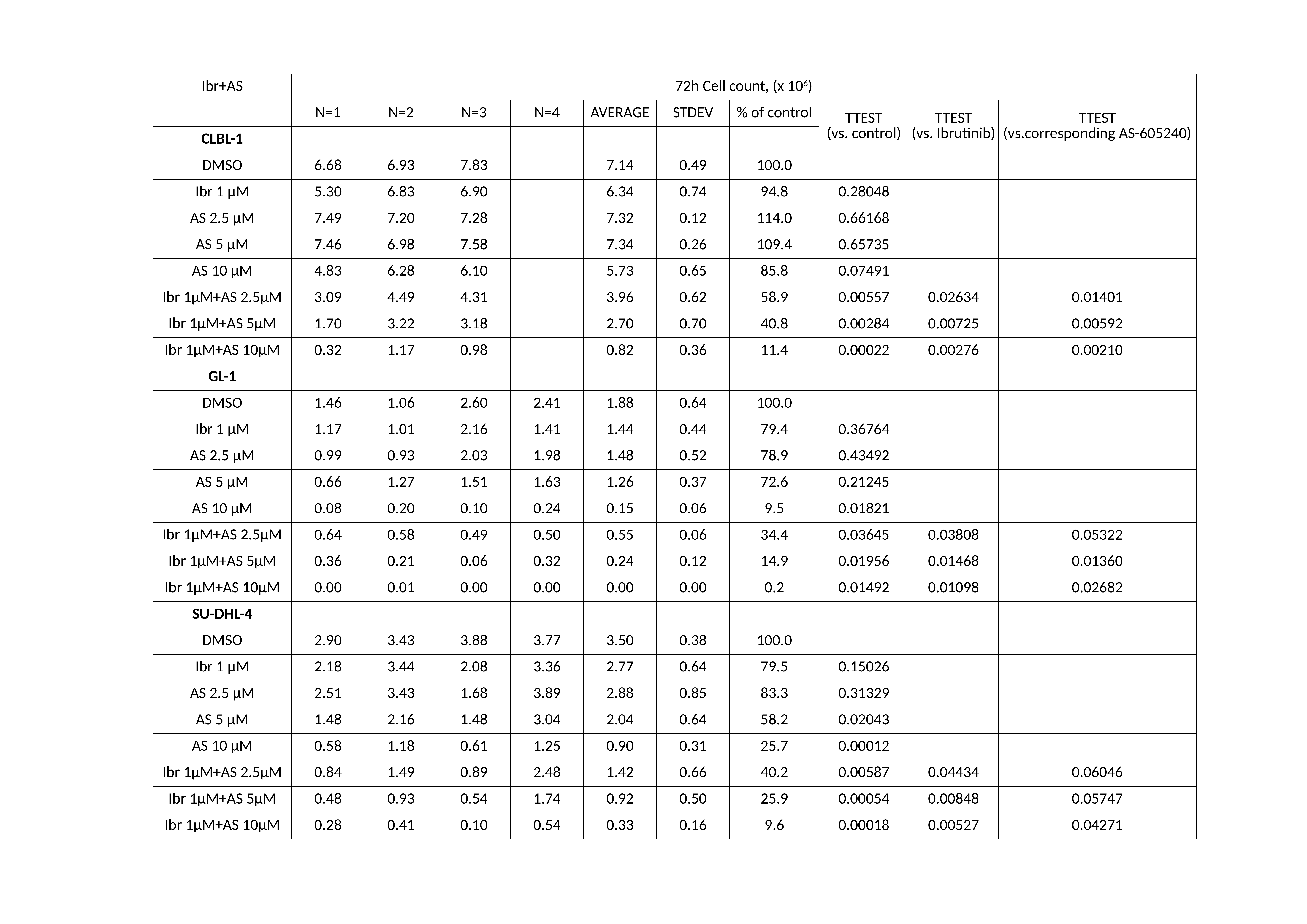

| Ibr+AS | 72h Cell count, (x 106) | | | | | | | | | |
| --- | --- | --- | --- | --- | --- | --- | --- | --- | --- | --- |
| | N=1 | N=2 | N=3 | N=4 | AVERAGE | STDEV | % of control | TTEST (vs. control) | TTEST (vs. Ibrutinib) | TTEST (vs.corresponding AS-605240) |
| CLBL-1 | | | | | | | | | | |
| DMSO | 6.68 | 6.93 | 7.83 | | 7.14 | 0.49 | 100.0 | | | |
| Ibr 1 µM | 5.30 | 6.83 | 6.90 | | 6.34 | 0.74 | 94.8 | 0.28048 | | |
| AS 2.5 µM | 7.49 | 7.20 | 7.28 | | 7.32 | 0.12 | 114.0 | 0.66168 | | |
| AS 5 µM | 7.46 | 6.98 | 7.58 | | 7.34 | 0.26 | 109.4 | 0.65735 | | |
| AS 10 µM | 4.83 | 6.28 | 6.10 | | 5.73 | 0.65 | 85.8 | 0.07491 | | |
| Ibr 1µM+AS 2.5µM | 3.09 | 4.49 | 4.31 | | 3.96 | 0.62 | 58.9 | 0.00557 | 0.02634 | 0.01401 |
| Ibr 1µM+AS 5µM | 1.70 | 3.22 | 3.18 | | 2.70 | 0.70 | 40.8 | 0.00284 | 0.00725 | 0.00592 |
| Ibr 1µM+AS 10µM | 0.32 | 1.17 | 0.98 | | 0.82 | 0.36 | 11.4 | 0.00022 | 0.00276 | 0.00210 |
| GL-1 | | | | | | | | | | |
| DMSO | 1.46 | 1.06 | 2.60 | 2.41 | 1.88 | 0.64 | 100.0 | | | |
| Ibr 1 µM | 1.17 | 1.01 | 2.16 | 1.41 | 1.44 | 0.44 | 79.4 | 0.36764 | | |
| AS 2.5 µM | 0.99 | 0.93 | 2.03 | 1.98 | 1.48 | 0.52 | 78.9 | 0.43492 | | |
| AS 5 µM | 0.66 | 1.27 | 1.51 | 1.63 | 1.26 | 0.37 | 72.6 | 0.21245 | | |
| AS 10 µM | 0.08 | 0.20 | 0.10 | 0.24 | 0.15 | 0.06 | 9.5 | 0.01821 | | |
| Ibr 1µM+AS 2.5µM | 0.64 | 0.58 | 0.49 | 0.50 | 0.55 | 0.06 | 34.4 | 0.03645 | 0.03808 | 0.05322 |
| Ibr 1µM+AS 5µM | 0.36 | 0.21 | 0.06 | 0.32 | 0.24 | 0.12 | 14.9 | 0.01956 | 0.01468 | 0.01360 |
| Ibr 1µM+AS 10µM | 0.00 | 0.01 | 0.00 | 0.00 | 0.00 | 0.00 | 0.2 | 0.01492 | 0.01098 | 0.02682 |
| SU-DHL-4 | | | | | | | | | | |
| DMSO | 2.90 | 3.43 | 3.88 | 3.77 | 3.50 | 0.38 | 100.0 | | | |
| Ibr 1 µM | 2.18 | 3.44 | 2.08 | 3.36 | 2.77 | 0.64 | 79.5 | 0.15026 | | |
| AS 2.5 µM | 2.51 | 3.43 | 1.68 | 3.89 | 2.88 | 0.85 | 83.3 | 0.31329 | | |
| AS 5 µM | 1.48 | 2.16 | 1.48 | 3.04 | 2.04 | 0.64 | 58.2 | 0.02043 | | |
| AS 10 µM | 0.58 | 1.18 | 0.61 | 1.25 | 0.90 | 0.31 | 25.7 | 0.00012 | | |
| Ibr 1µM+AS 2.5µM | 0.84 | 1.49 | 0.89 | 2.48 | 1.42 | 0.66 | 40.2 | 0.00587 | 0.04434 | 0.06046 |
| Ibr 1µM+AS 5µM | 0.48 | 0.93 | 0.54 | 1.74 | 0.92 | 0.50 | 25.9 | 0.00054 | 0.00848 | 0.05747 |
| Ibr 1µM+AS 10µM | 0.28 | 0.41 | 0.10 | 0.54 | 0.33 | 0.16 | 9.6 | 0.00018 | 0.00527 | 0.04271 |

## Slide 50
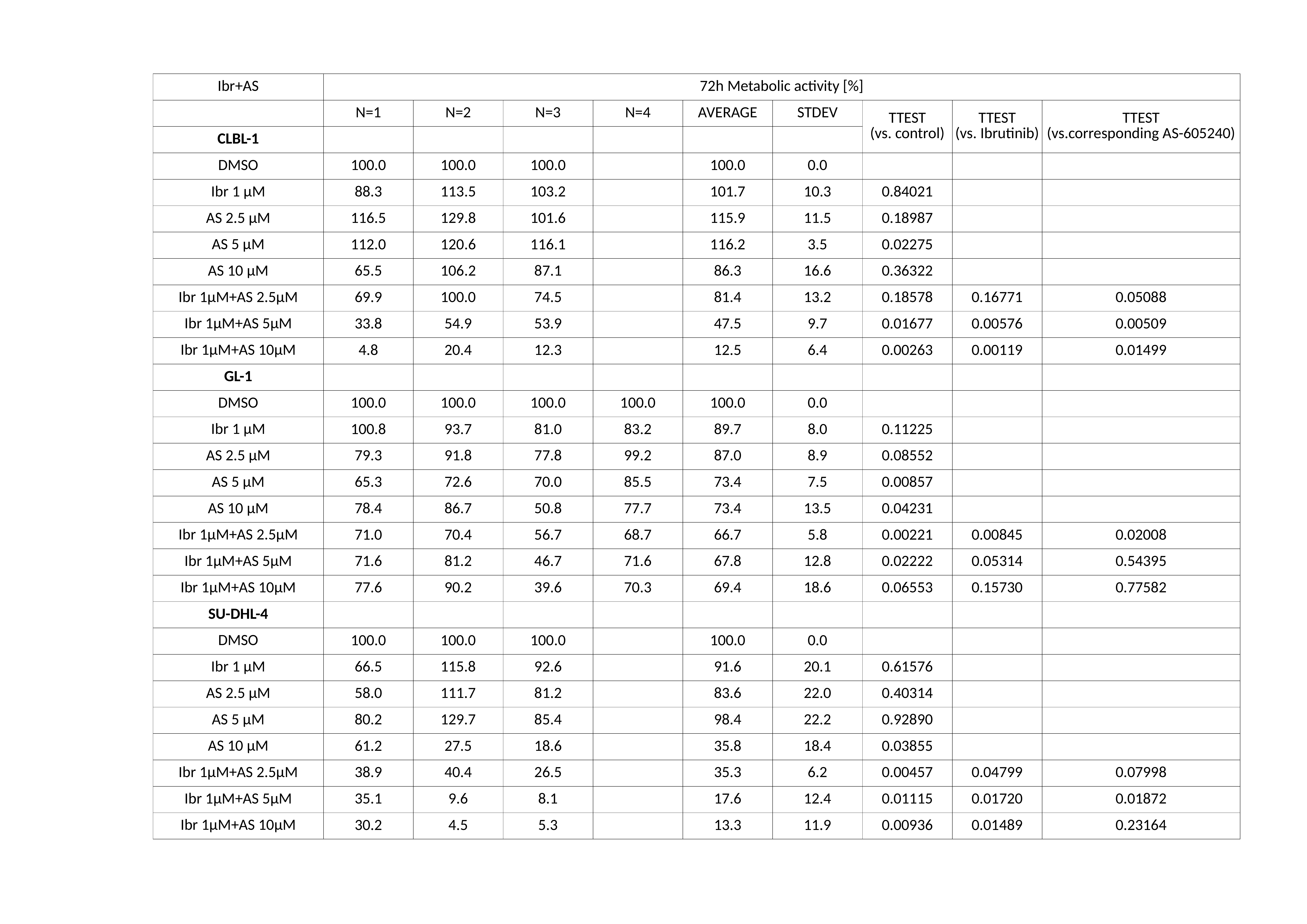

| Ibr+AS | 72h Metabolic activity [%] | | | | | | | | |
| --- | --- | --- | --- | --- | --- | --- | --- | --- | --- |
| | N=1 | N=2 | N=3 | N=4 | AVERAGE | STDEV | TTEST (vs. control) | TTEST (vs. Ibrutinib) | TTEST (vs.corresponding AS-605240) |
| CLBL-1 | | | | | | | | | |
| DMSO | 100.0 | 100.0 | 100.0 | | 100.0 | 0.0 | | | |
| Ibr 1 µM | 88.3 | 113.5 | 103.2 | | 101.7 | 10.3 | 0.84021 | | |
| AS 2.5 µM | 116.5 | 129.8 | 101.6 | | 115.9 | 11.5 | 0.18987 | | |
| AS 5 µM | 112.0 | 120.6 | 116.1 | | 116.2 | 3.5 | 0.02275 | | |
| AS 10 µM | 65.5 | 106.2 | 87.1 | | 86.3 | 16.6 | 0.36322 | | |
| Ibr 1µM+AS 2.5µM | 69.9 | 100.0 | 74.5 | | 81.4 | 13.2 | 0.18578 | 0.16771 | 0.05088 |
| Ibr 1µM+AS 5µM | 33.8 | 54.9 | 53.9 | | 47.5 | 9.7 | 0.01677 | 0.00576 | 0.00509 |
| Ibr 1µM+AS 10µM | 4.8 | 20.4 | 12.3 | | 12.5 | 6.4 | 0.00263 | 0.00119 | 0.01499 |
| GL-1 | | | | | | | | | |
| DMSO | 100.0 | 100.0 | 100.0 | 100.0 | 100.0 | 0.0 | | | |
| Ibr 1 µM | 100.8 | 93.7 | 81.0 | 83.2 | 89.7 | 8.0 | 0.11225 | | |
| AS 2.5 µM | 79.3 | 91.8 | 77.8 | 99.2 | 87.0 | 8.9 | 0.08552 | | |
| AS 5 µM | 65.3 | 72.6 | 70.0 | 85.5 | 73.4 | 7.5 | 0.00857 | | |
| AS 10 µM | 78.4 | 86.7 | 50.8 | 77.7 | 73.4 | 13.5 | 0.04231 | | |
| Ibr 1µM+AS 2.5µM | 71.0 | 70.4 | 56.7 | 68.7 | 66.7 | 5.8 | 0.00221 | 0.00845 | 0.02008 |
| Ibr 1µM+AS 5µM | 71.6 | 81.2 | 46.7 | 71.6 | 67.8 | 12.8 | 0.02222 | 0.05314 | 0.54395 |
| Ibr 1µM+AS 10µM | 77.6 | 90.2 | 39.6 | 70.3 | 69.4 | 18.6 | 0.06553 | 0.15730 | 0.77582 |
| SU-DHL-4 | | | | | | | | | |
| DMSO | 100.0 | 100.0 | 100.0 | | 100.0 | 0.0 | | | |
| Ibr 1 µM | 66.5 | 115.8 | 92.6 | | 91.6 | 20.1 | 0.61576 | | |
| AS 2.5 µM | 58.0 | 111.7 | 81.2 | | 83.6 | 22.0 | 0.40314 | | |
| AS 5 µM | 80.2 | 129.7 | 85.4 | | 98.4 | 22.2 | 0.92890 | | |
| AS 10 µM | 61.2 | 27.5 | 18.6 | | 35.8 | 18.4 | 0.03855 | | |
| Ibr 1µM+AS 2.5µM | 38.9 | 40.4 | 26.5 | | 35.3 | 6.2 | 0.00457 | 0.04799 | 0.07998 |
| Ibr 1µM+AS 5µM | 35.1 | 9.6 | 8.1 | | 17.6 | 12.4 | 0.01115 | 0.01720 | 0.01872 |
| Ibr 1µM+AS 10µM | 30.2 | 4.5 | 5.3 | | 13.3 | 11.9 | 0.00936 | 0.01489 | 0.23164 |

## Slide 51
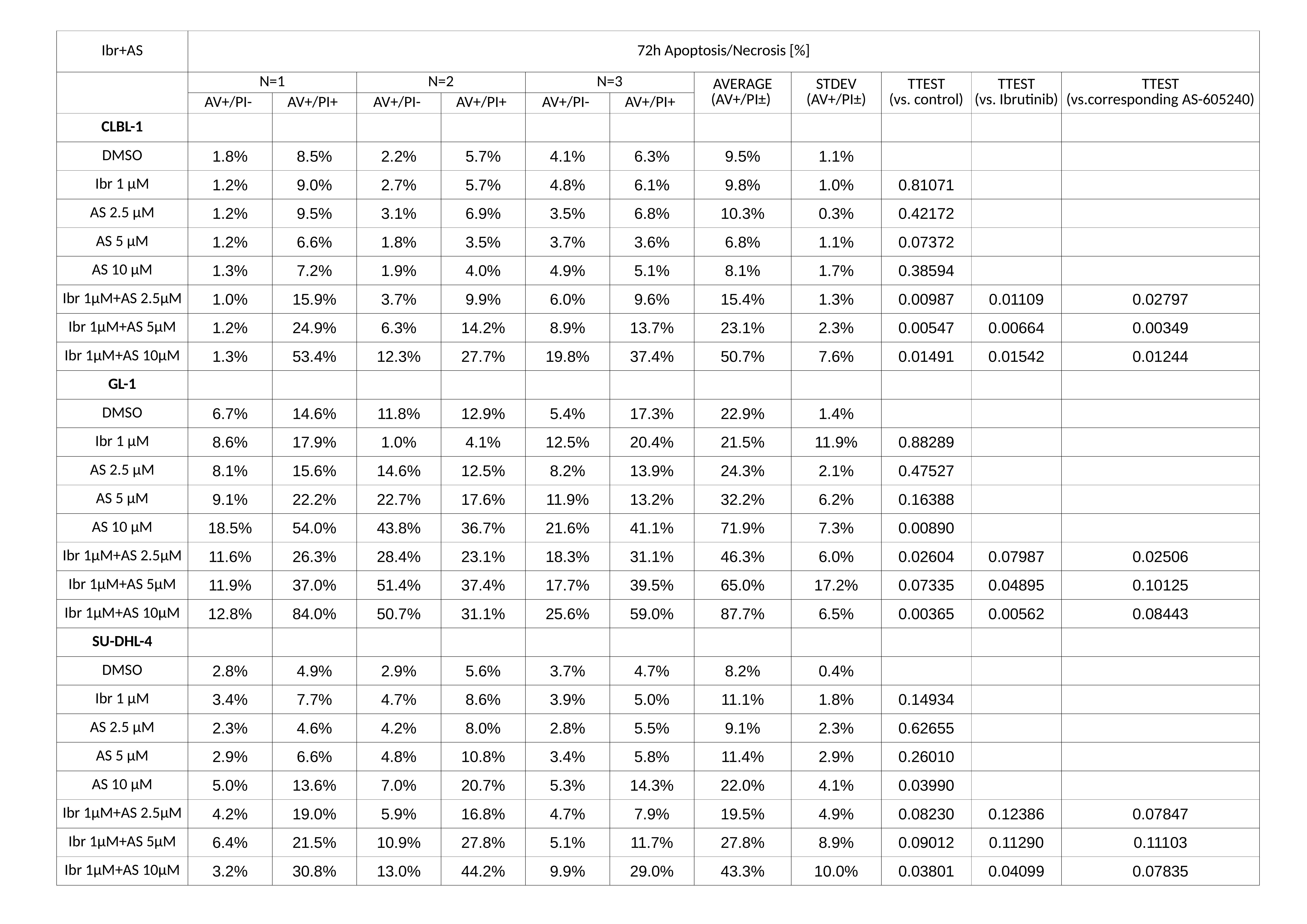

| Ibr+AS | 72h Apoptosis/Necrosis [%] | | | | | | | | | | |
| --- | --- | --- | --- | --- | --- | --- | --- | --- | --- | --- | --- |
| | N=1 | | N=2 | | N=3 | | AVERAGE (AV+/PI±) | STDEV (AV+/PI±) | TTEST (vs. control) | TTEST (vs. Ibrutinib) | TTEST (vs.corresponding AS-605240) |
| | AV+/PI- | AV+/PI+ | AV+/PI- | AV+/PI+ | AV+/PI- | AV+/PI+ | | | | | |
| CLBL-1 | | | | | | | | | | | |
| DMSO | 1.8% | 8.5% | 2.2% | 5.7% | 4.1% | 6.3% | 9.5% | 1.1% | | | |
| Ibr 1 µM | 1.2% | 9.0% | 2.7% | 5.7% | 4.8% | 6.1% | 9.8% | 1.0% | 0.81071 | | |
| AS 2.5 µM | 1.2% | 9.5% | 3.1% | 6.9% | 3.5% | 6.8% | 10.3% | 0.3% | 0.42172 | | |
| AS 5 µM | 1.2% | 6.6% | 1.8% | 3.5% | 3.7% | 3.6% | 6.8% | 1.1% | 0.07372 | | |
| AS 10 µM | 1.3% | 7.2% | 1.9% | 4.0% | 4.9% | 5.1% | 8.1% | 1.7% | 0.38594 | | |
| Ibr 1µM+AS 2.5µM | 1.0% | 15.9% | 3.7% | 9.9% | 6.0% | 9.6% | 15.4% | 1.3% | 0.00987 | 0.01109 | 0.02797 |
| Ibr 1µM+AS 5µM | 1.2% | 24.9% | 6.3% | 14.2% | 8.9% | 13.7% | 23.1% | 2.3% | 0.00547 | 0.00664 | 0.00349 |
| Ibr 1µM+AS 10µM | 1.3% | 53.4% | 12.3% | 27.7% | 19.8% | 37.4% | 50.7% | 7.6% | 0.01491 | 0.01542 | 0.01244 |
| GL-1 | | | | | | | | | | | |
| DMSO | 6.7% | 14.6% | 11.8% | 12.9% | 5.4% | 17.3% | 22.9% | 1.4% | | | |
| Ibr 1 µM | 8.6% | 17.9% | 1.0% | 4.1% | 12.5% | 20.4% | 21.5% | 11.9% | 0.88289 | | |
| AS 2.5 µM | 8.1% | 15.6% | 14.6% | 12.5% | 8.2% | 13.9% | 24.3% | 2.1% | 0.47527 | | |
| AS 5 µM | 9.1% | 22.2% | 22.7% | 17.6% | 11.9% | 13.2% | 32.2% | 6.2% | 0.16388 | | |
| AS 10 µM | 18.5% | 54.0% | 43.8% | 36.7% | 21.6% | 41.1% | 71.9% | 7.3% | 0.00890 | | |
| Ibr 1µM+AS 2.5µM | 11.6% | 26.3% | 28.4% | 23.1% | 18.3% | 31.1% | 46.3% | 6.0% | 0.02604 | 0.07987 | 0.02506 |
| Ibr 1µM+AS 5µM | 11.9% | 37.0% | 51.4% | 37.4% | 17.7% | 39.5% | 65.0% | 17.2% | 0.07335 | 0.04895 | 0.10125 |
| Ibr 1µM+AS 10µM | 12.8% | 84.0% | 50.7% | 31.1% | 25.6% | 59.0% | 87.7% | 6.5% | 0.00365 | 0.00562 | 0.08443 |
| SU-DHL-4 | | | | | | | | | | | |
| DMSO | 2.8% | 4.9% | 2.9% | 5.6% | 3.7% | 4.7% | 8.2% | 0.4% | | | |
| Ibr 1 µM | 3.4% | 7.7% | 4.7% | 8.6% | 3.9% | 5.0% | 11.1% | 1.8% | 0.14934 | | |
| AS 2.5 µM | 2.3% | 4.6% | 4.2% | 8.0% | 2.8% | 5.5% | 9.1% | 2.3% | 0.62655 | | |
| AS 5 µM | 2.9% | 6.6% | 4.8% | 10.8% | 3.4% | 5.8% | 11.4% | 2.9% | 0.26010 | | |
| AS 10 µM | 5.0% | 13.6% | 7.0% | 20.7% | 5.3% | 14.3% | 22.0% | 4.1% | 0.03990 | | |
| Ibr 1µM+AS 2.5µM | 4.2% | 19.0% | 5.9% | 16.8% | 4.7% | 7.9% | 19.5% | 4.9% | 0.08230 | 0.12386 | 0.07847 |
| Ibr 1µM+AS 5µM | 6.4% | 21.5% | 10.9% | 27.8% | 5.1% | 11.7% | 27.8% | 8.9% | 0.09012 | 0.11290 | 0.11103 |
| Ibr 1µM+AS 10µM | 3.2% | 30.8% | 13.0% | 44.2% | 9.9% | 29.0% | 43.3% | 10.0% | 0.03801 | 0.04099 | 0.07835 |

## Slide 52
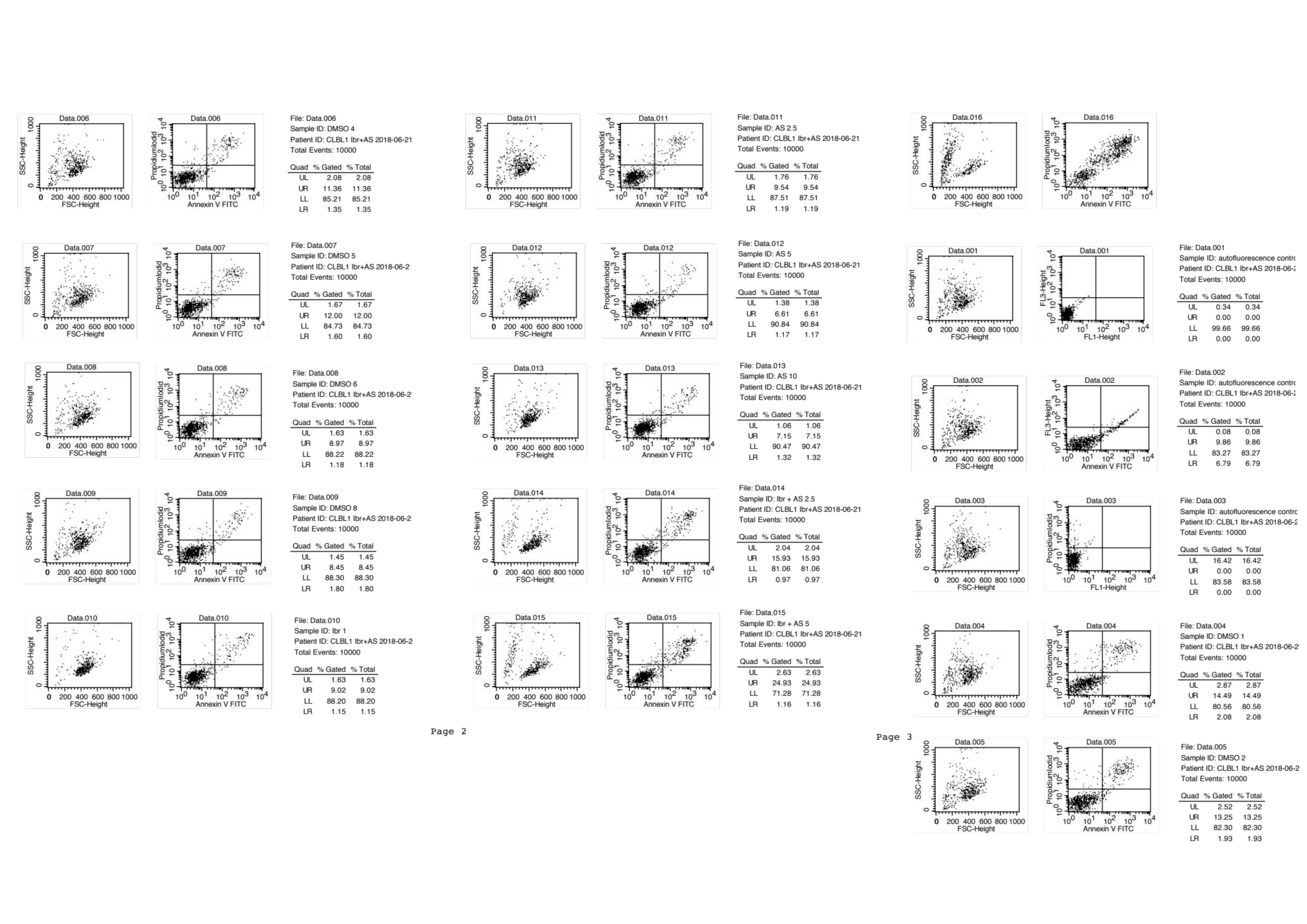

## Slide 53
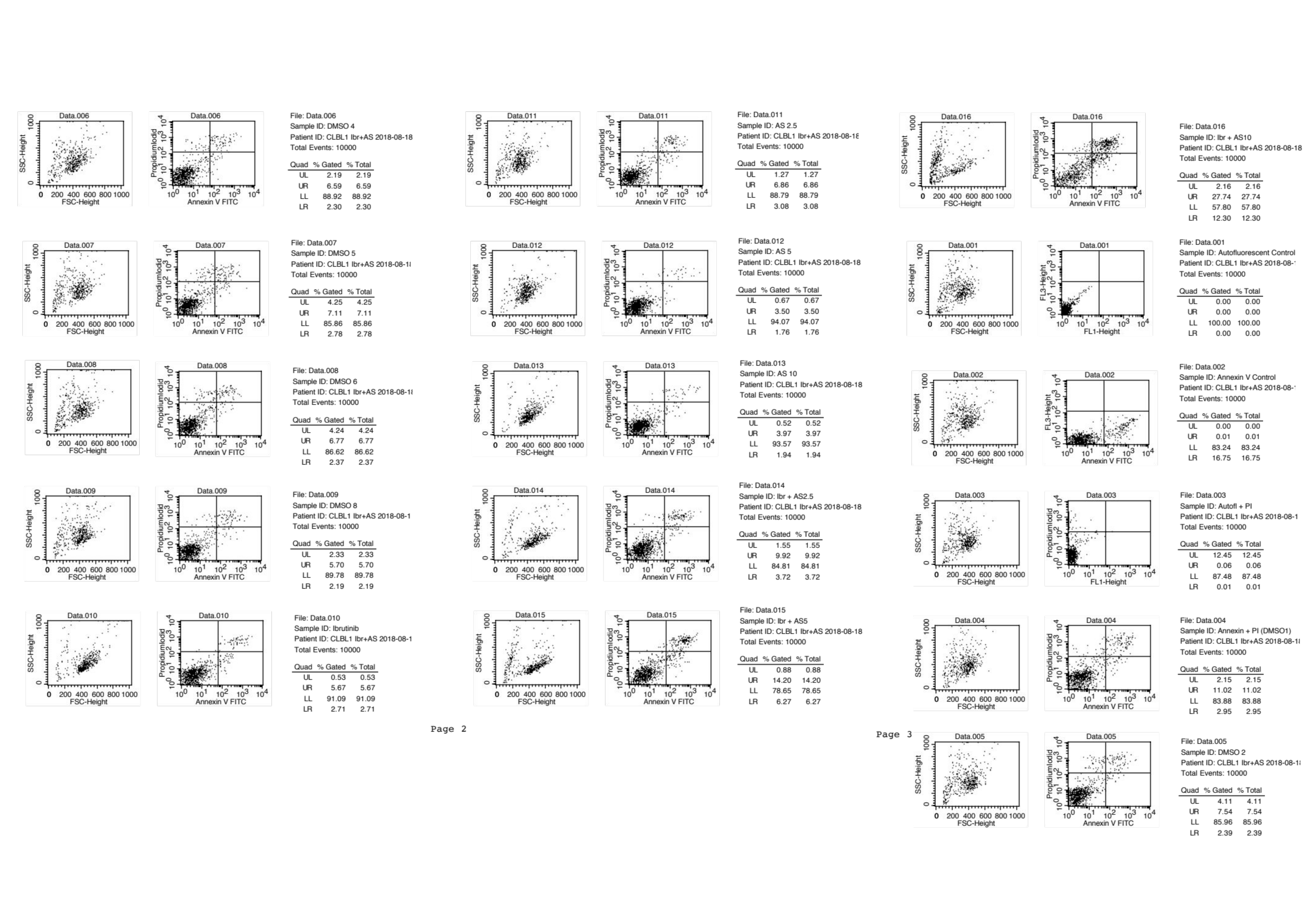

## Slide 54
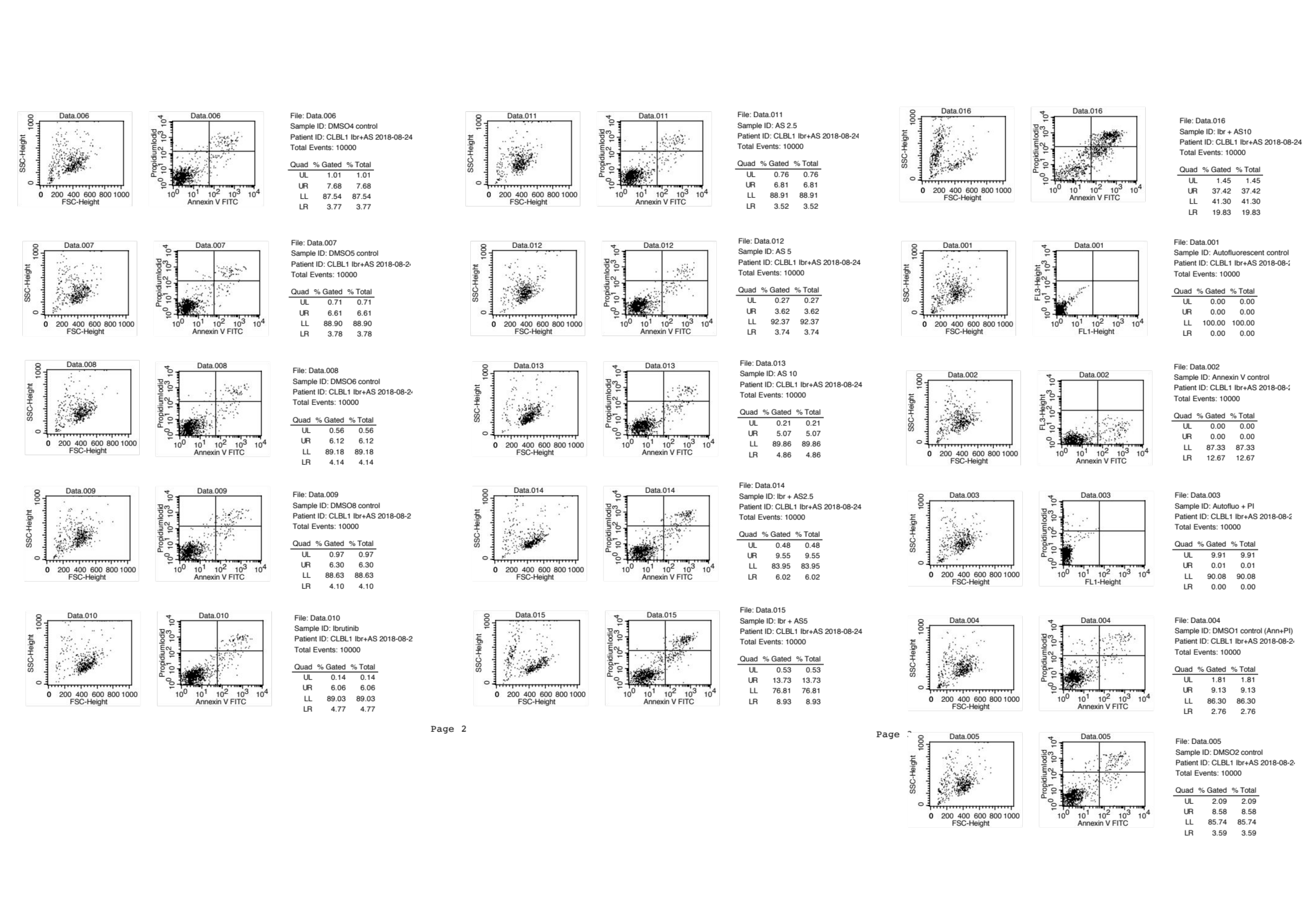

## Slide 55
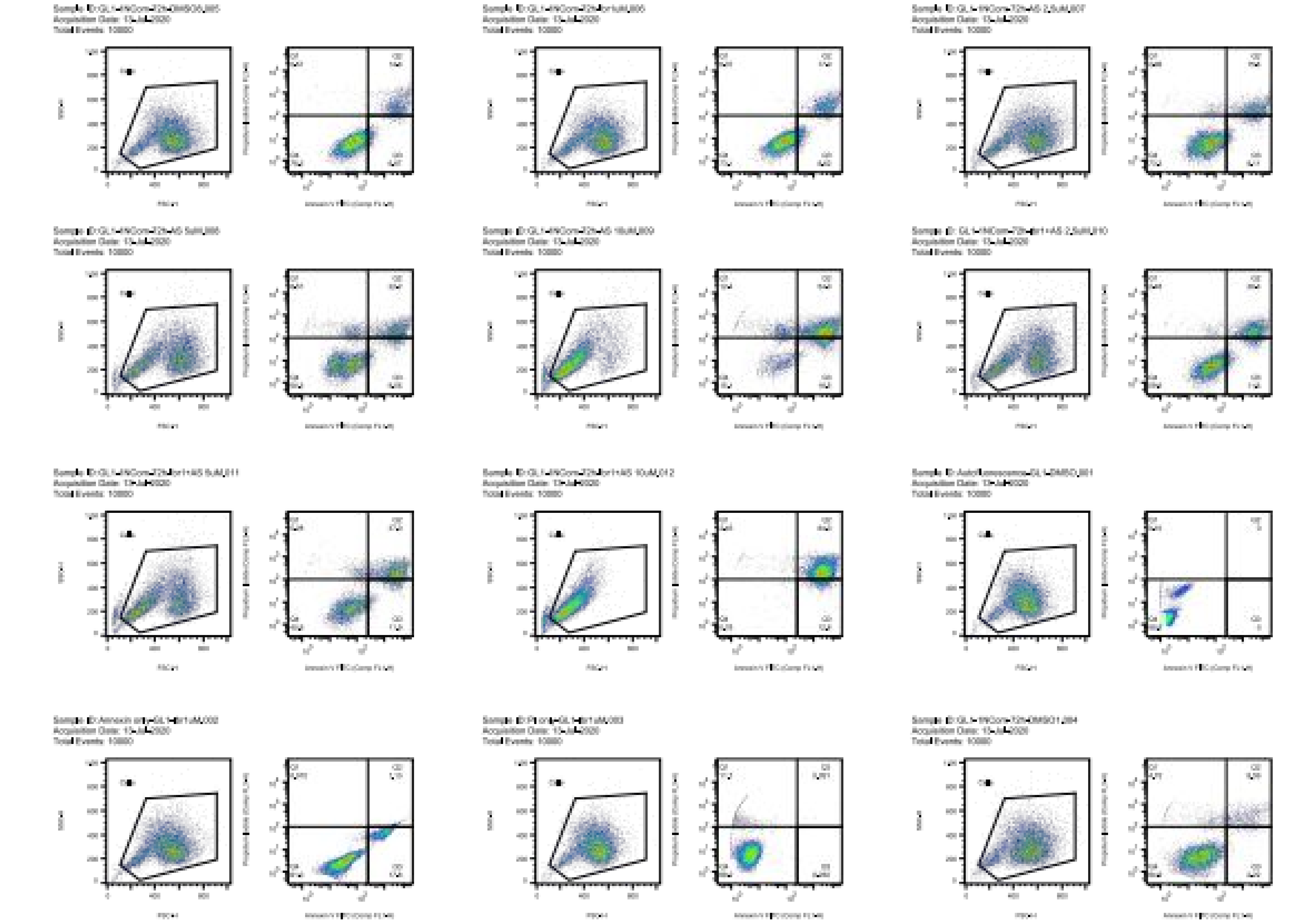

## Slide 56
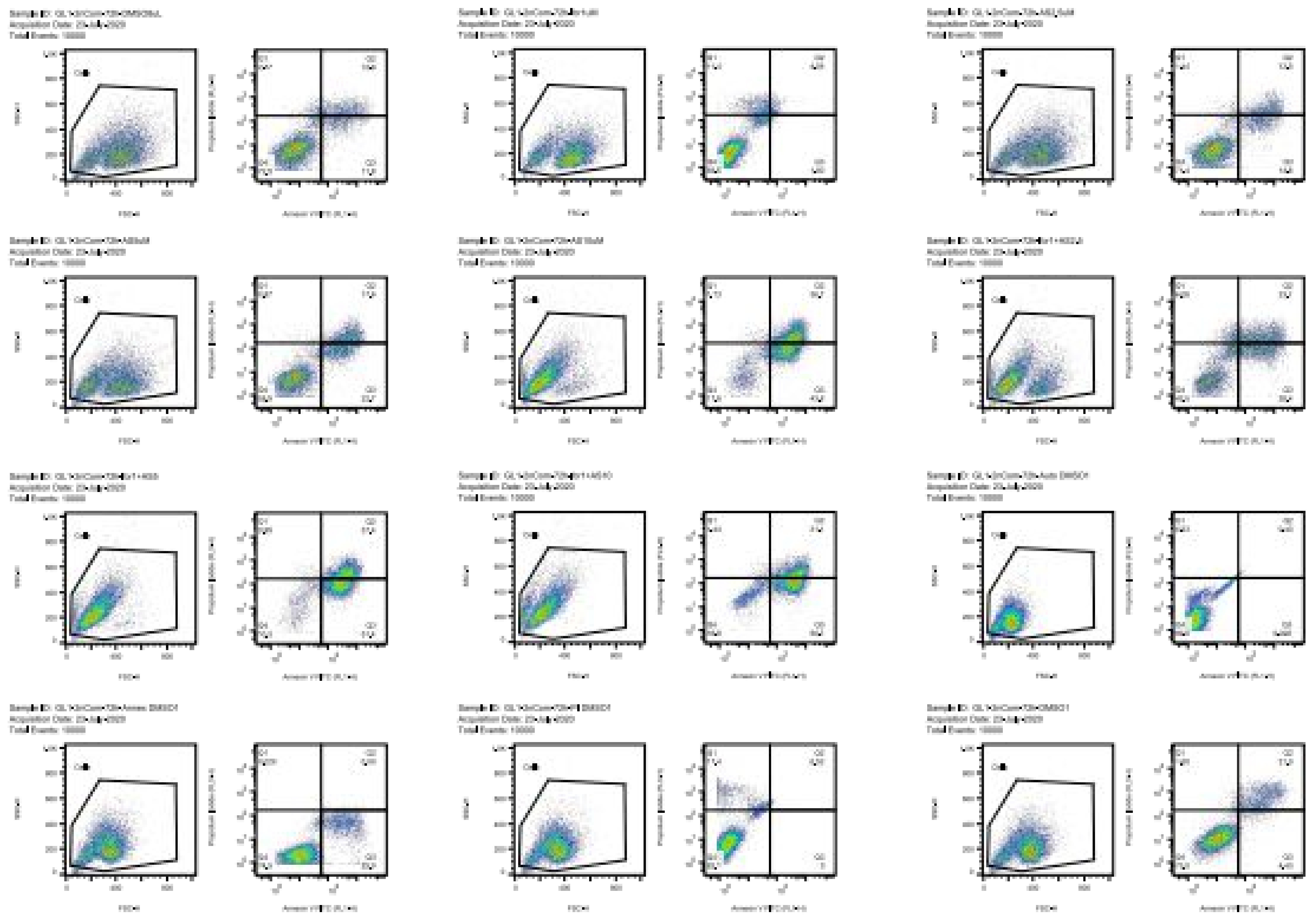

## Slide 57
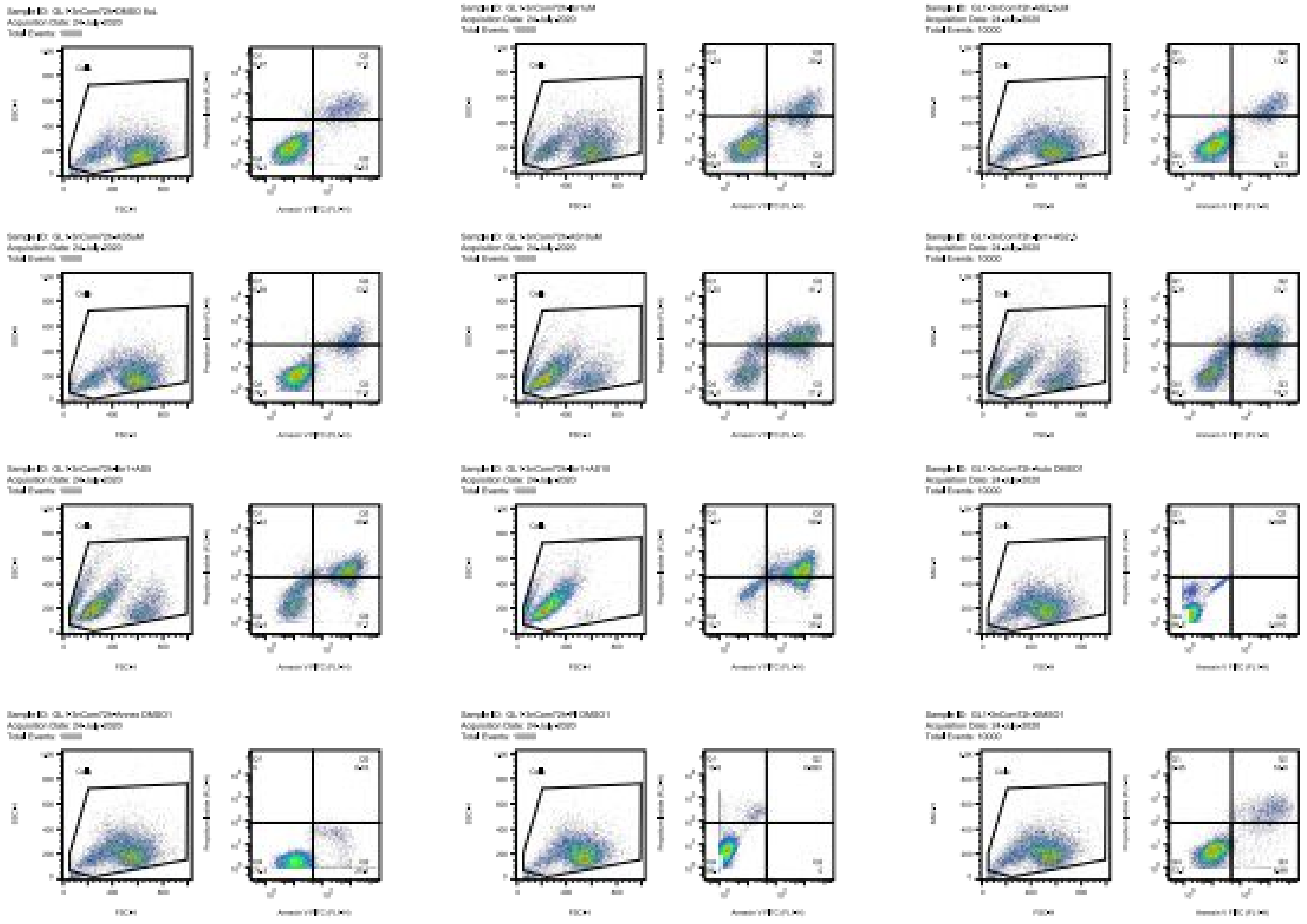

## Slide 58
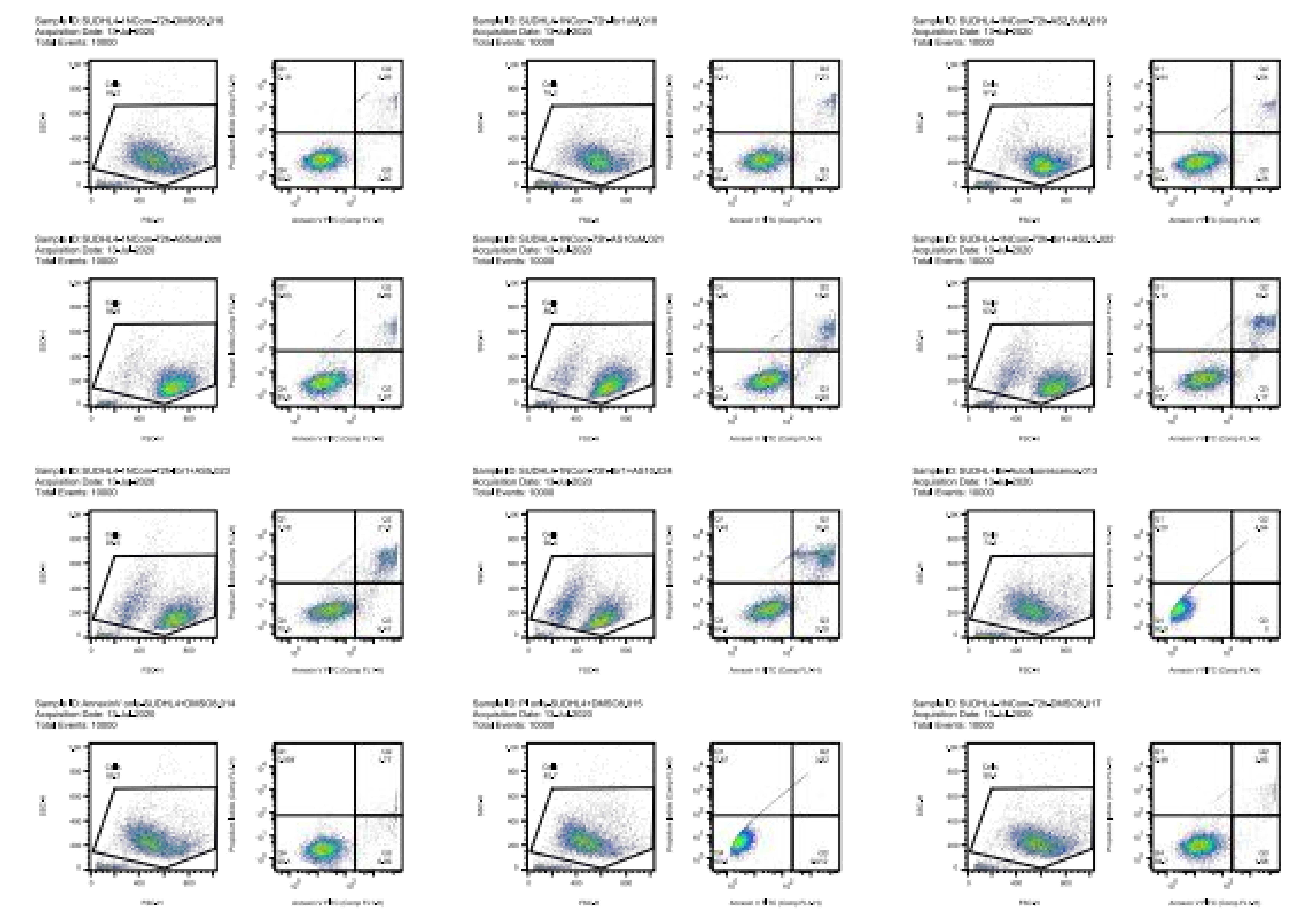

## Slide 59
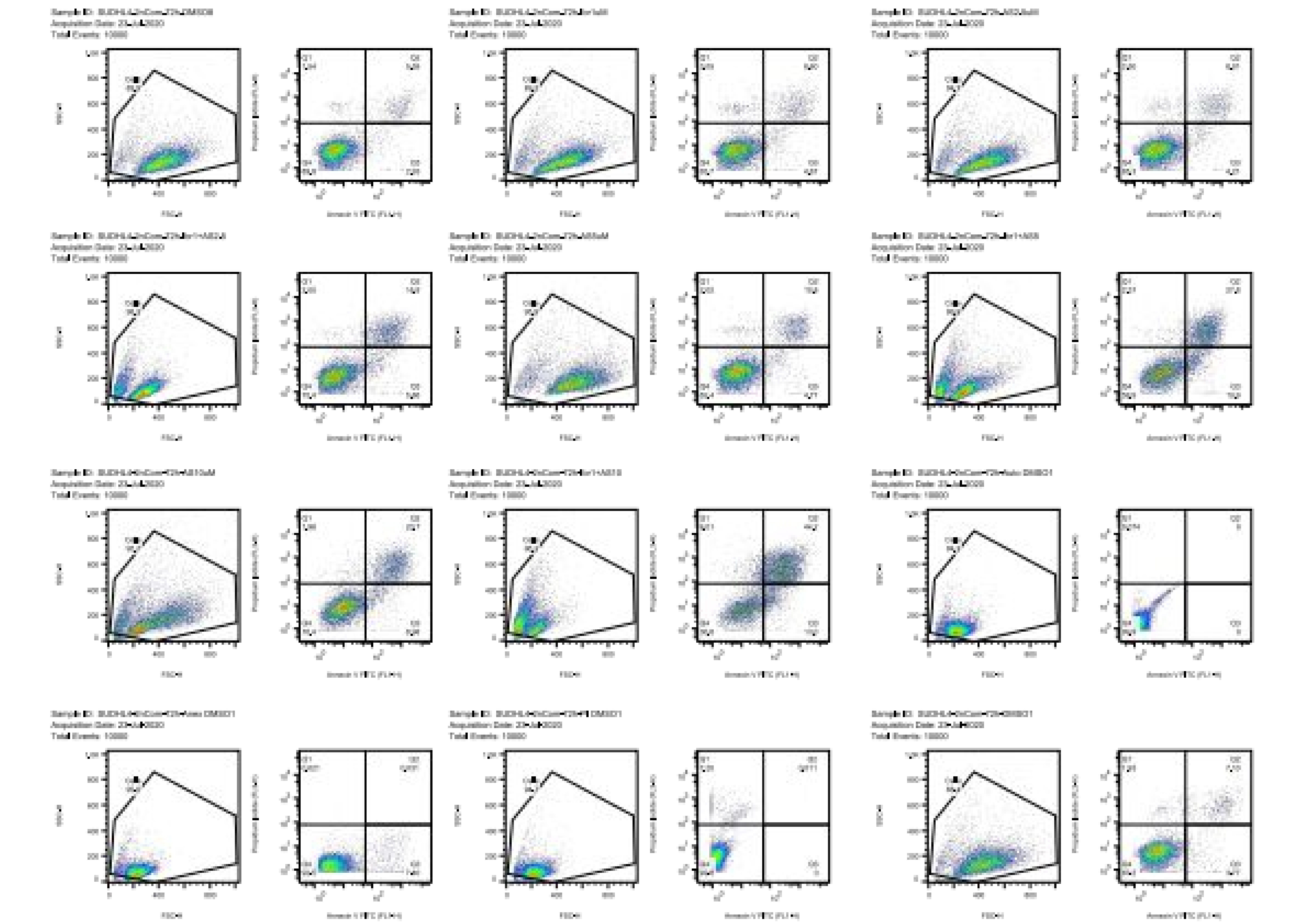

## Slide 60
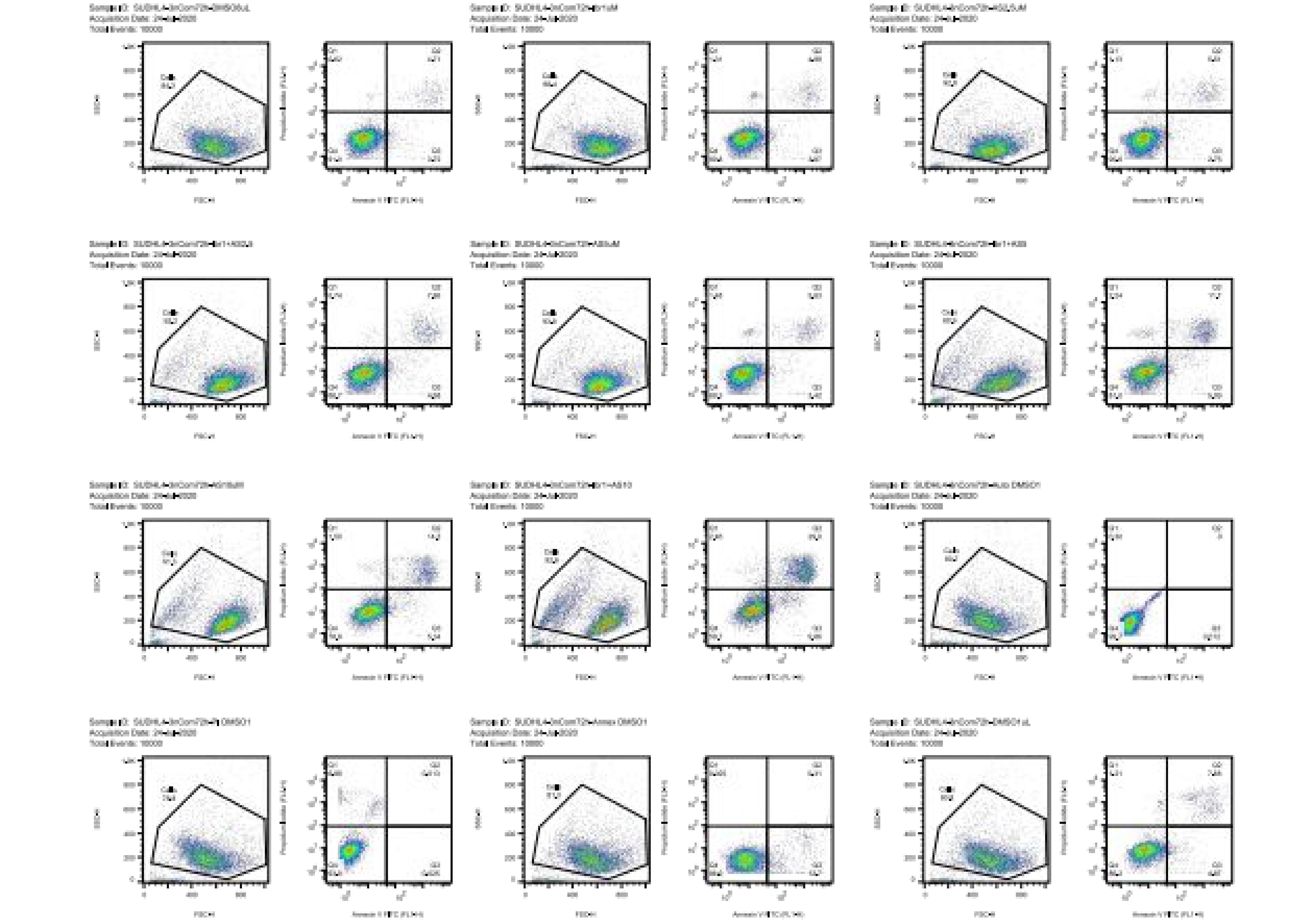

## Slide 61
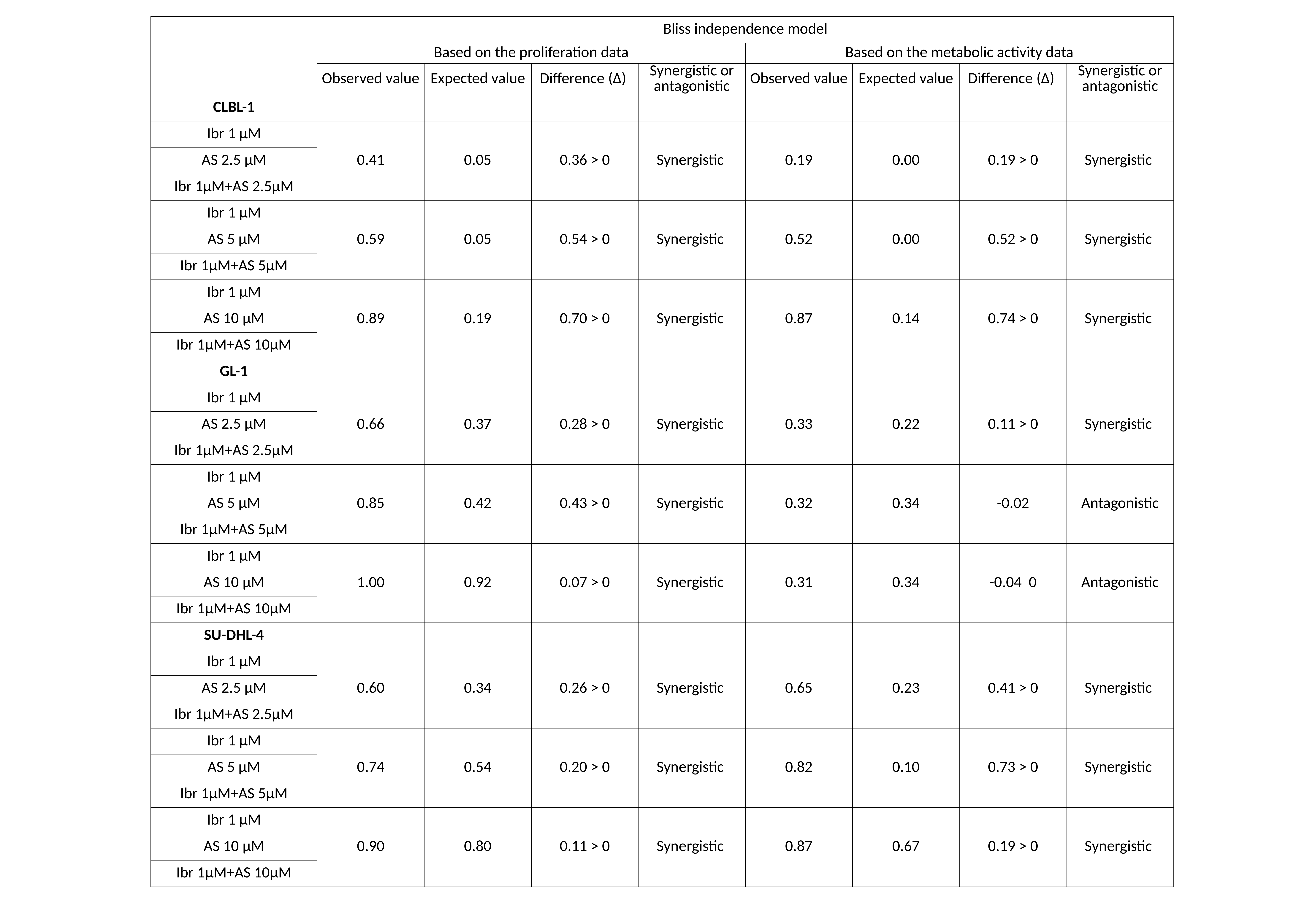

## Slide 62
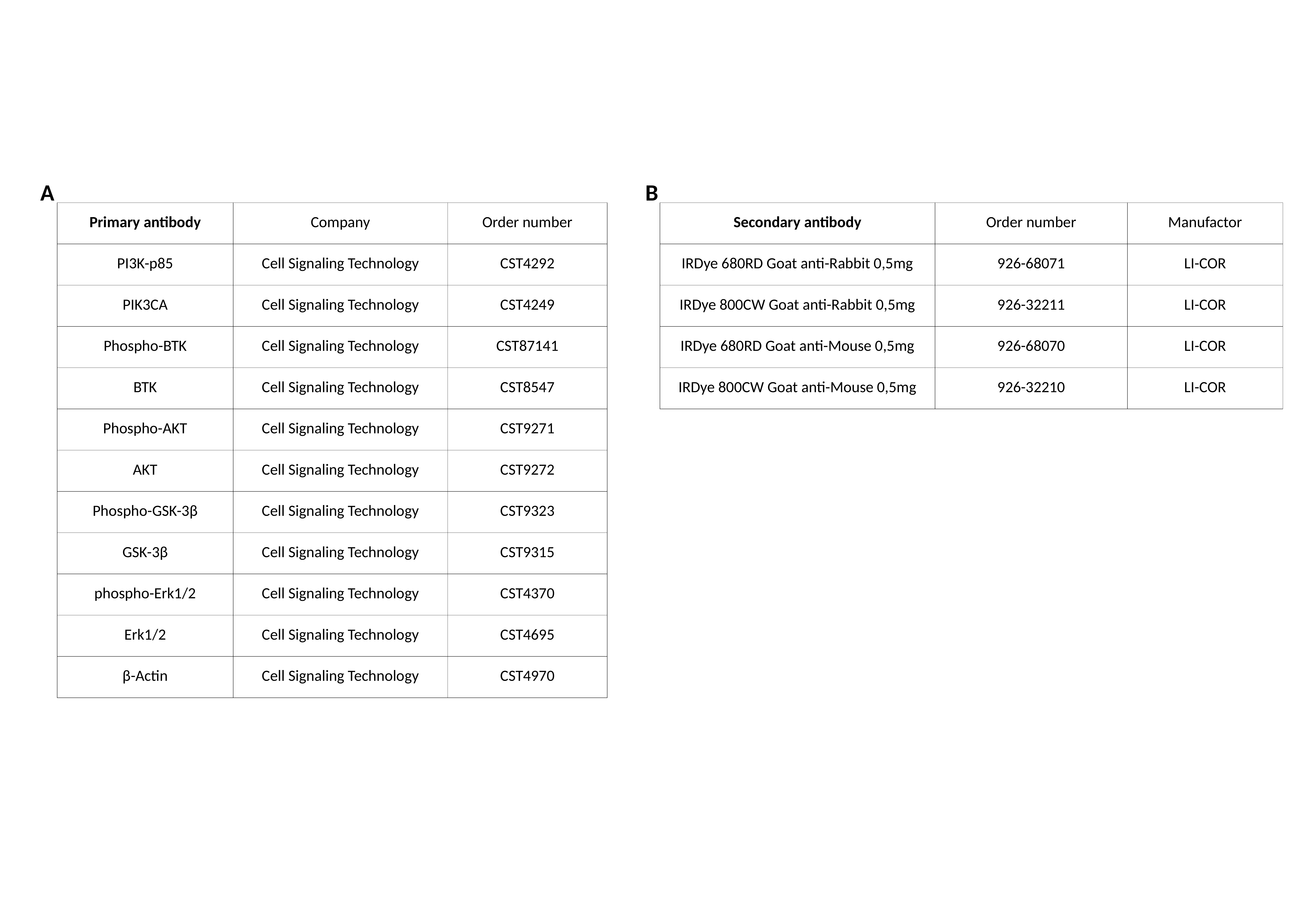

A
B
| Primary antibody | Company | Order number |
| --- | --- | --- |
| PI3K-p85 | Cell Signaling Technology | CST4292 |
| PIK3CA | Cell Signaling Technology | CST4249 |
| Phospho-BTK | Cell Signaling Technology | CST87141 |
| BTK | Cell Signaling Technology | CST8547 |
| Phospho-AKT | Cell Signaling Technology | CST9271 |
| AKT | Cell Signaling Technology | CST9272 |
| Phospho-GSK-3β | Cell Signaling Technology | CST9323 |
| GSK-3β | Cell Signaling Technology | CST9315 |
| phospho-Erk1/2 | Cell Signaling Technology | CST4370 |
| Erk1/2 | Cell Signaling Technology | CST4695 |
| β-Actin | Cell Signaling Technology | CST4970 |
| Secondary antibody | Order number | Manufactor |
| --- | --- | --- |
| IRDye 680RD Goat anti-Rabbit 0,5mg | 926-68071 | LI-COR |
| IRDye 800CW Goat anti-Rabbit 0,5mg | 926-32211 | LI-COR |
| IRDye 680RD Goat anti-Mouse 0,5mg | 926-68070 | LI-COR |
| IRDye 800CW Goat anti-Mouse 0,5mg | 926-32210 | LI-COR |

## Slide 63
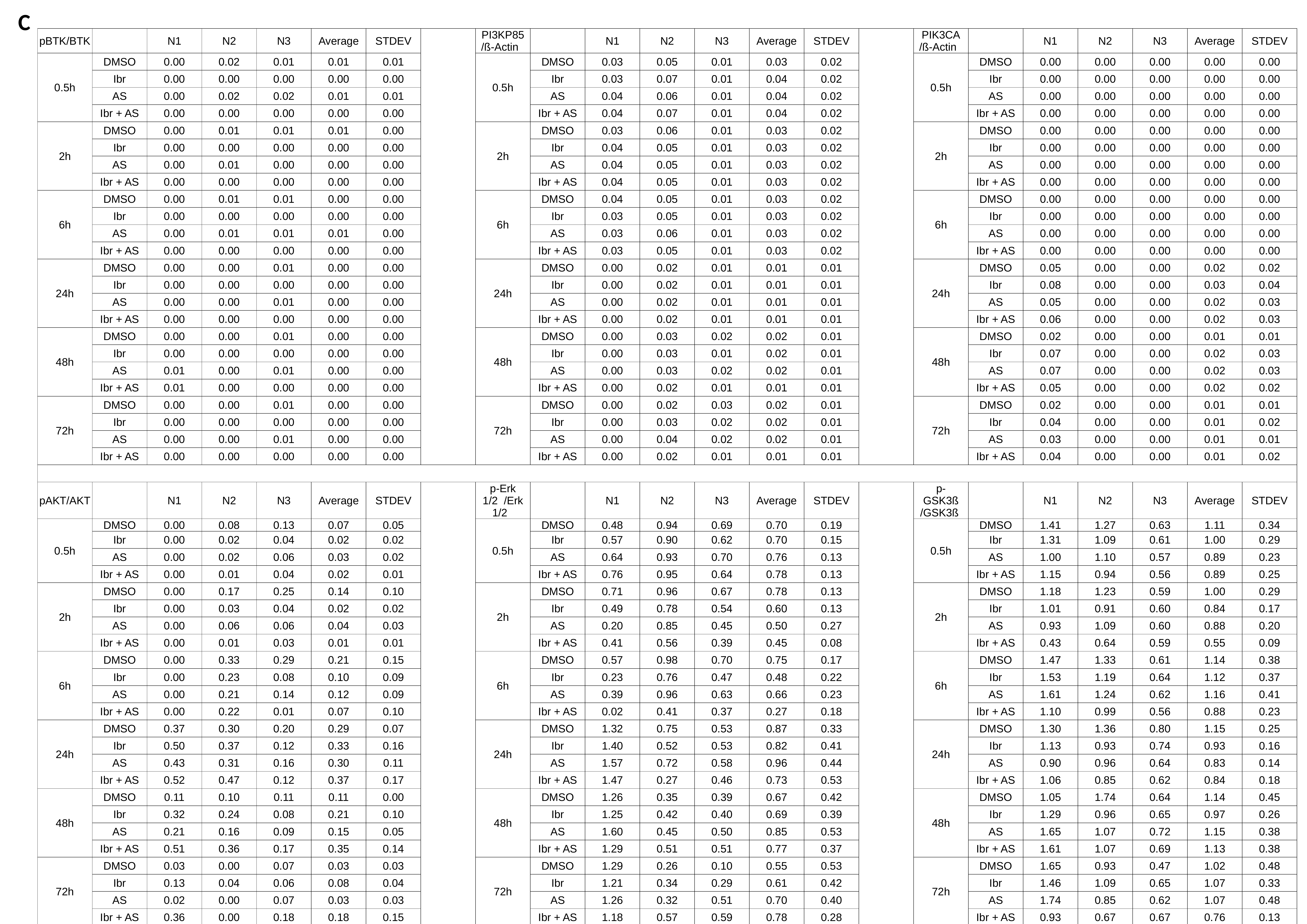

C
| pBTK/BTK | | N1 | N2 | N3 | Average | STDEV | | PI3KP85 /ß-Actin | | N1 | N2 | N3 | Average | STDEV | | PIK3CA /ß-Actin | | N1 | N2 | N3 | Average | STDEV |
| --- | --- | --- | --- | --- | --- | --- | --- | --- | --- | --- | --- | --- | --- | --- | --- | --- | --- | --- | --- | --- | --- | --- |
| 0.5h | DMSO | 0.00 | 0.02 | 0.01 | 0.01 | 0.01 | | 0.5h | DMSO | 0.03 | 0.05 | 0.01 | 0.03 | 0.02 | | 0.5h | DMSO | 0.00 | 0.00 | 0.00 | 0.00 | 0.00 |
| | Ibr | 0.00 | 0.00 | 0.00 | 0.00 | 0.00 | | | Ibr | 0.03 | 0.07 | 0.01 | 0.04 | 0.02 | | | Ibr | 0.00 | 0.00 | 0.00 | 0.00 | 0.00 |
| | AS | 0.00 | 0.02 | 0.02 | 0.01 | 0.01 | | | AS | 0.04 | 0.06 | 0.01 | 0.04 | 0.02 | | | AS | 0.00 | 0.00 | 0.00 | 0.00 | 0.00 |
| | Ibr + AS | 0.00 | 0.00 | 0.00 | 0.00 | 0.00 | | | Ibr + AS | 0.04 | 0.07 | 0.01 | 0.04 | 0.02 | | | Ibr + AS | 0.00 | 0.00 | 0.00 | 0.00 | 0.00 |
| 2h | DMSO | 0.00 | 0.01 | 0.01 | 0.01 | 0.00 | | 2h | DMSO | 0.03 | 0.06 | 0.01 | 0.03 | 0.02 | | 2h | DMSO | 0.00 | 0.00 | 0.00 | 0.00 | 0.00 |
| | Ibr | 0.00 | 0.00 | 0.00 | 0.00 | 0.00 | | | Ibr | 0.04 | 0.05 | 0.01 | 0.03 | 0.02 | | | Ibr | 0.00 | 0.00 | 0.00 | 0.00 | 0.00 |
| | AS | 0.00 | 0.01 | 0.00 | 0.00 | 0.00 | | | AS | 0.04 | 0.05 | 0.01 | 0.03 | 0.02 | | | AS | 0.00 | 0.00 | 0.00 | 0.00 | 0.00 |
| | Ibr + AS | 0.00 | 0.00 | 0.00 | 0.00 | 0.00 | | | Ibr + AS | 0.04 | 0.05 | 0.01 | 0.03 | 0.02 | | | Ibr + AS | 0.00 | 0.00 | 0.00 | 0.00 | 0.00 |
| 6h | DMSO | 0.00 | 0.01 | 0.01 | 0.00 | 0.00 | | 6h | DMSO | 0.04 | 0.05 | 0.01 | 0.03 | 0.02 | | 6h | DMSO | 0.00 | 0.00 | 0.00 | 0.00 | 0.00 |
| | Ibr | 0.00 | 0.00 | 0.00 | 0.00 | 0.00 | | | Ibr | 0.03 | 0.05 | 0.01 | 0.03 | 0.02 | | | Ibr | 0.00 | 0.00 | 0.00 | 0.00 | 0.00 |
| | AS | 0.00 | 0.01 | 0.01 | 0.01 | 0.00 | | | AS | 0.03 | 0.06 | 0.01 | 0.03 | 0.02 | | | AS | 0.00 | 0.00 | 0.00 | 0.00 | 0.00 |
| | Ibr + AS | 0.00 | 0.00 | 0.00 | 0.00 | 0.00 | | | Ibr + AS | 0.03 | 0.05 | 0.01 | 0.03 | 0.02 | | | Ibr + AS | 0.00 | 0.00 | 0.00 | 0.00 | 0.00 |
| 24h | DMSO | 0.00 | 0.00 | 0.01 | 0.00 | 0.00 | | 24h | DMSO | 0.00 | 0.02 | 0.01 | 0.01 | 0.01 | | 24h | DMSO | 0.05 | 0.00 | 0.00 | 0.02 | 0.02 |
| | Ibr | 0.00 | 0.00 | 0.00 | 0.00 | 0.00 | | | Ibr | 0.00 | 0.02 | 0.01 | 0.01 | 0.01 | | | Ibr | 0.08 | 0.00 | 0.00 | 0.03 | 0.04 |
| | AS | 0.00 | 0.00 | 0.01 | 0.00 | 0.00 | | | AS | 0.00 | 0.02 | 0.01 | 0.01 | 0.01 | | | AS | 0.05 | 0.00 | 0.00 | 0.02 | 0.03 |
| | Ibr + AS | 0.00 | 0.00 | 0.00 | 0.00 | 0.00 | | | Ibr + AS | 0.00 | 0.02 | 0.01 | 0.01 | 0.01 | | | Ibr + AS | 0.06 | 0.00 | 0.00 | 0.02 | 0.03 |
| 48h | DMSO | 0.00 | 0.00 | 0.01 | 0.00 | 0.00 | | 48h | DMSO | 0.00 | 0.03 | 0.02 | 0.02 | 0.01 | | 48h | DMSO | 0.02 | 0.00 | 0.00 | 0.01 | 0.01 |
| | Ibr | 0.00 | 0.00 | 0.00 | 0.00 | 0.00 | | | Ibr | 0.00 | 0.03 | 0.01 | 0.02 | 0.01 | | | Ibr | 0.07 | 0.00 | 0.00 | 0.02 | 0.03 |
| | AS | 0.01 | 0.00 | 0.01 | 0.00 | 0.00 | | | AS | 0.00 | 0.03 | 0.02 | 0.02 | 0.01 | | | AS | 0.07 | 0.00 | 0.00 | 0.02 | 0.03 |
| | Ibr + AS | 0.01 | 0.00 | 0.00 | 0.00 | 0.00 | | | Ibr + AS | 0.00 | 0.02 | 0.01 | 0.01 | 0.01 | | | Ibr + AS | 0.05 | 0.00 | 0.00 | 0.02 | 0.02 |
| 72h | DMSO | 0.00 | 0.00 | 0.01 | 0.00 | 0.00 | | 72h | DMSO | 0.00 | 0.02 | 0.03 | 0.02 | 0.01 | | 72h | DMSO | 0.02 | 0.00 | 0.00 | 0.01 | 0.01 |
| | Ibr | 0.00 | 0.00 | 0.00 | 0.00 | 0.00 | | | Ibr | 0.00 | 0.03 | 0.02 | 0.02 | 0.01 | | | Ibr | 0.04 | 0.00 | 0.00 | 0.01 | 0.02 |
| | AS | 0.00 | 0.00 | 0.01 | 0.00 | 0.00 | | | AS | 0.00 | 0.04 | 0.02 | 0.02 | 0.01 | | | AS | 0.03 | 0.00 | 0.00 | 0.01 | 0.01 |
| | Ibr + AS | 0.00 | 0.00 | 0.00 | 0.00 | 0.00 | | | Ibr + AS | 0.00 | 0.02 | 0.01 | 0.01 | 0.01 | | | Ibr + AS | 0.04 | 0.00 | 0.00 | 0.01 | 0.02 |
| | | | | | | | | | | | | | | | | | | | | | | |
| pAKT/AKT | | N1 | N2 | N3 | Average | STDEV | | p-Erk 1/2 /Erk 1/2 | | N1 | N2 | N3 | Average | STDEV | | p-GSK3ß /GSK3ß | | N1 | N2 | N3 | Average | STDEV |
| 0.5h | DMSO | 0.00 | 0.08 | 0.13 | 0.07 | 0.05 | | 0.5h | DMSO | 0.48 | 0.94 | 0.69 | 0.70 | 0.19 | | 0.5h | DMSO | 1.41 | 1.27 | 0.63 | 1.11 | 0.34 |
| | Ibr | 0.00 | 0.02 | 0.04 | 0.02 | 0.02 | | | Ibr | 0.57 | 0.90 | 0.62 | 0.70 | 0.15 | | | Ibr | 1.31 | 1.09 | 0.61 | 1.00 | 0.29 |
| | AS | 0.00 | 0.02 | 0.06 | 0.03 | 0.02 | | | AS | 0.64 | 0.93 | 0.70 | 0.76 | 0.13 | | | AS | 1.00 | 1.10 | 0.57 | 0.89 | 0.23 |
| | Ibr + AS | 0.00 | 0.01 | 0.04 | 0.02 | 0.01 | | | Ibr + AS | 0.76 | 0.95 | 0.64 | 0.78 | 0.13 | | | Ibr + AS | 1.15 | 0.94 | 0.56 | 0.89 | 0.25 |
| 2h | DMSO | 0.00 | 0.17 | 0.25 | 0.14 | 0.10 | | 2h | DMSO | 0.71 | 0.96 | 0.67 | 0.78 | 0.13 | | 2h | DMSO | 1.18 | 1.23 | 0.59 | 1.00 | 0.29 |
| | Ibr | 0.00 | 0.03 | 0.04 | 0.02 | 0.02 | | | Ibr | 0.49 | 0.78 | 0.54 | 0.60 | 0.13 | | | Ibr | 1.01 | 0.91 | 0.60 | 0.84 | 0.17 |
| | AS | 0.00 | 0.06 | 0.06 | 0.04 | 0.03 | | | AS | 0.20 | 0.85 | 0.45 | 0.50 | 0.27 | | | AS | 0.93 | 1.09 | 0.60 | 0.88 | 0.20 |
| | Ibr + AS | 0.00 | 0.01 | 0.03 | 0.01 | 0.01 | | | Ibr + AS | 0.41 | 0.56 | 0.39 | 0.45 | 0.08 | | | Ibr + AS | 0.43 | 0.64 | 0.59 | 0.55 | 0.09 |
| 6h | DMSO | 0.00 | 0.33 | 0.29 | 0.21 | 0.15 | | 6h | DMSO | 0.57 | 0.98 | 0.70 | 0.75 | 0.17 | | 6h | DMSO | 1.47 | 1.33 | 0.61 | 1.14 | 0.38 |
| | Ibr | 0.00 | 0.23 | 0.08 | 0.10 | 0.09 | | | Ibr | 0.23 | 0.76 | 0.47 | 0.48 | 0.22 | | | Ibr | 1.53 | 1.19 | 0.64 | 1.12 | 0.37 |
| | AS | 0.00 | 0.21 | 0.14 | 0.12 | 0.09 | | | AS | 0.39 | 0.96 | 0.63 | 0.66 | 0.23 | | | AS | 1.61 | 1.24 | 0.62 | 1.16 | 0.41 |
| | Ibr + AS | 0.00 | 0.22 | 0.01 | 0.07 | 0.10 | | | Ibr + AS | 0.02 | 0.41 | 0.37 | 0.27 | 0.18 | | | Ibr + AS | 1.10 | 0.99 | 0.56 | 0.88 | 0.23 |
| 24h | DMSO | 0.37 | 0.30 | 0.20 | 0.29 | 0.07 | | 24h | DMSO | 1.32 | 0.75 | 0.53 | 0.87 | 0.33 | | 24h | DMSO | 1.30 | 1.36 | 0.80 | 1.15 | 0.25 |
| | Ibr | 0.50 | 0.37 | 0.12 | 0.33 | 0.16 | | | Ibr | 1.40 | 0.52 | 0.53 | 0.82 | 0.41 | | | Ibr | 1.13 | 0.93 | 0.74 | 0.93 | 0.16 |
| | AS | 0.43 | 0.31 | 0.16 | 0.30 | 0.11 | | | AS | 1.57 | 0.72 | 0.58 | 0.96 | 0.44 | | | AS | 0.90 | 0.96 | 0.64 | 0.83 | 0.14 |
| | Ibr + AS | 0.52 | 0.47 | 0.12 | 0.37 | 0.17 | | | Ibr + AS | 1.47 | 0.27 | 0.46 | 0.73 | 0.53 | | | Ibr + AS | 1.06 | 0.85 | 0.62 | 0.84 | 0.18 |
| 48h | DMSO | 0.11 | 0.10 | 0.11 | 0.11 | 0.00 | | 48h | DMSO | 1.26 | 0.35 | 0.39 | 0.67 | 0.42 | | 48h | DMSO | 1.05 | 1.74 | 0.64 | 1.14 | 0.45 |
| | Ibr | 0.32 | 0.24 | 0.08 | 0.21 | 0.10 | | | Ibr | 1.25 | 0.42 | 0.40 | 0.69 | 0.39 | | | Ibr | 1.29 | 0.96 | 0.65 | 0.97 | 0.26 |
| | AS | 0.21 | 0.16 | 0.09 | 0.15 | 0.05 | | | AS | 1.60 | 0.45 | 0.50 | 0.85 | 0.53 | | | AS | 1.65 | 1.07 | 0.72 | 1.15 | 0.38 |
| | Ibr + AS | 0.51 | 0.36 | 0.17 | 0.35 | 0.14 | | | Ibr + AS | 1.29 | 0.51 | 0.51 | 0.77 | 0.37 | | | Ibr + AS | 1.61 | 1.07 | 0.69 | 1.13 | 0.38 |
| 72h | DMSO | 0.03 | 0.00 | 0.07 | 0.03 | 0.03 | | 72h | DMSO | 1.29 | 0.26 | 0.10 | 0.55 | 0.53 | | 72h | DMSO | 1.65 | 0.93 | 0.47 | 1.02 | 0.48 |
| | Ibr | 0.13 | 0.04 | 0.06 | 0.08 | 0.04 | | | Ibr | 1.21 | 0.34 | 0.29 | 0.61 | 0.42 | | | Ibr | 1.46 | 1.09 | 0.65 | 1.07 | 0.33 |
| | AS | 0.02 | 0.00 | 0.07 | 0.03 | 0.03 | | | AS | 1.26 | 0.32 | 0.51 | 0.70 | 0.40 | | | AS | 1.74 | 0.85 | 0.62 | 1.07 | 0.48 |
| | Ibr + AS | 0.36 | 0.00 | 0.18 | 0.18 | 0.15 | | | Ibr + AS | 1.18 | 0.57 | 0.59 | 0.78 | 0.28 | | | Ibr + AS | 0.93 | 0.67 | 0.67 | 0.76 | 0.13 |

## Slide 64
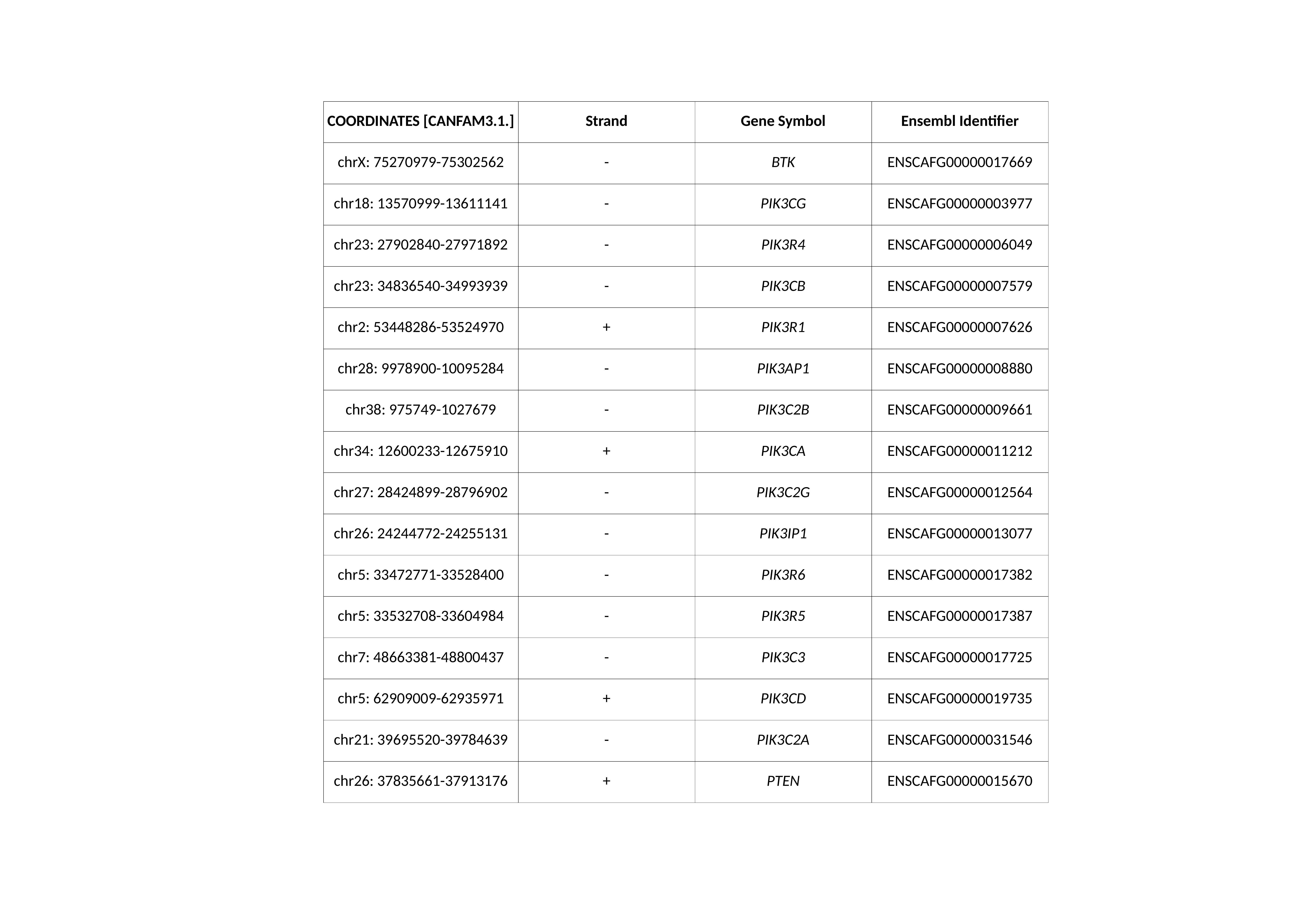

| Coordinates [canFam3.1.] | Strand | Gene Symbol | Ensembl Identifier |
| --- | --- | --- | --- |
| chrX: 75270979-75302562 | - | BTK | ENSCAFG00000017669 |
| chr18: 13570999-13611141 | - | PIK3CG | ENSCAFG00000003977 |
| chr23: 27902840-27971892 | - | PIK3R4 | ENSCAFG00000006049 |
| chr23: 34836540-34993939 | - | PIK3CB | ENSCAFG00000007579 |
| chr2: 53448286-53524970 | + | PIK3R1 | ENSCAFG00000007626 |
| chr28: 9978900-10095284 | - | PIK3AP1 | ENSCAFG00000008880 |
| chr38: 975749-1027679 | - | PIK3C2B | ENSCAFG00000009661 |
| chr34: 12600233-12675910 | + | PIK3CA | ENSCAFG00000011212 |
| chr27: 28424899-28796902 | - | PIK3C2G | ENSCAFG00000012564 |
| chr26: 24244772-24255131 | - | PIK3IP1 | ENSCAFG00000013077 |
| chr5: 33472771-33528400 | - | PIK3R6 | ENSCAFG00000017382 |
| chr5: 33532708-33604984 | - | PIK3R5 | ENSCAFG00000017387 |
| chr7: 48663381-48800437 | - | PIK3C3 | ENSCAFG00000017725 |
| chr5: 62909009-62935971 | + | PIK3CD | ENSCAFG00000019735 |
| chr21: 39695520-39784639 | - | PIK3C2A | ENSCAFG00000031546 |
| chr26: 37835661-37913176 | + | PTEN | ENSCAFG00000015670 |

## Slide 65
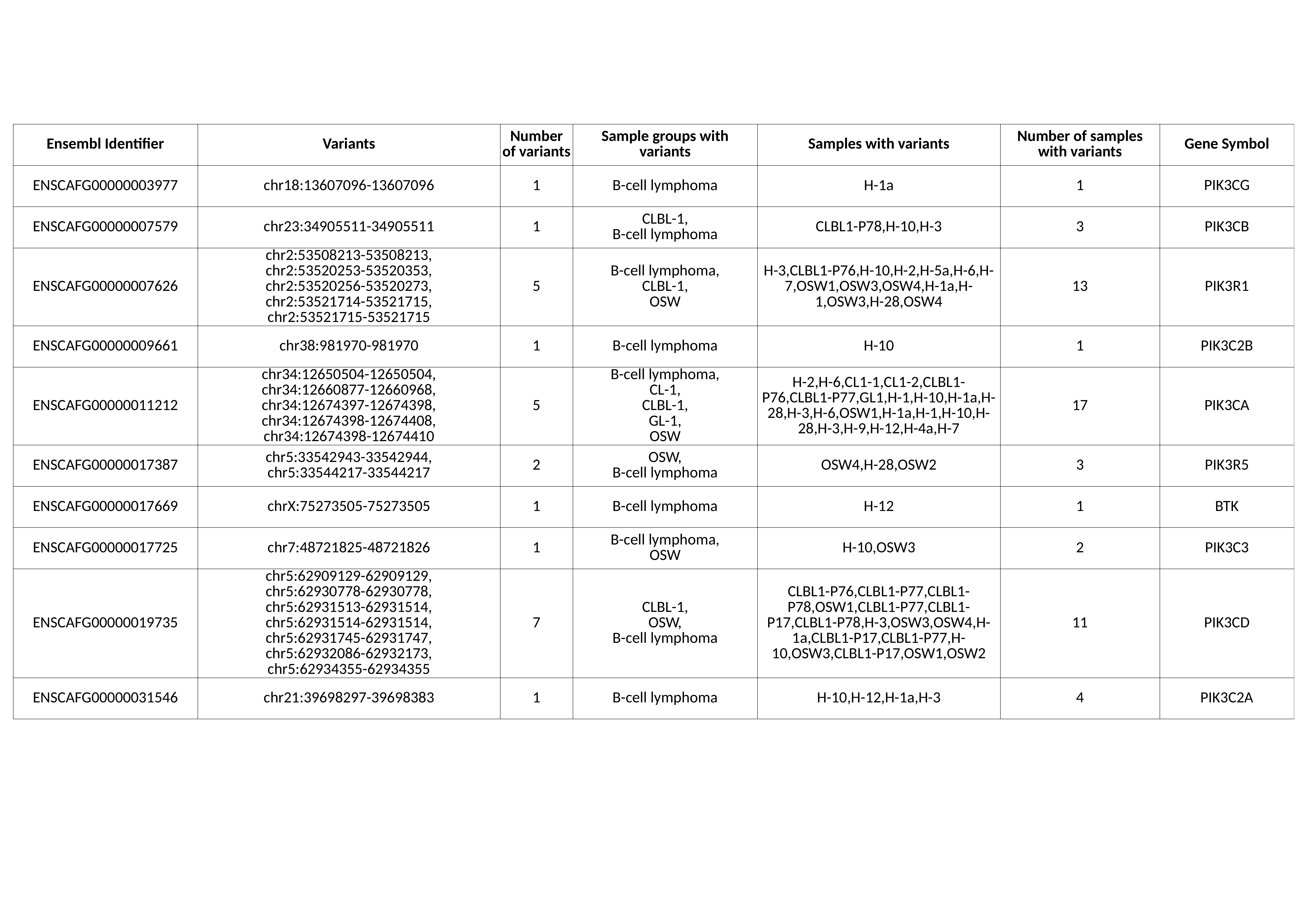

| Ensembl Identifier | Variants | Number of variants | Sample groups with variants | Samples with variants | Number of samples with variants | Gene Symbol |
| --- | --- | --- | --- | --- | --- | --- |
| ENSCAFG00000003977 | chr18:13607096-13607096 | 1 | B-cell lymphoma | H-1a | 1 | PIK3CG |
| ENSCAFG00000007579 | chr23:34905511-34905511 | 1 | CLBL-1, B-cell lymphoma | CLBL1-P78,H-10,H-3 | 3 | PIK3CB |
| ENSCAFG00000007626 | chr2:53508213-53508213, chr2:53520253-53520353, chr2:53520256-53520273, chr2:53521714-53521715, chr2:53521715-53521715 | 5 | B-cell lymphoma, CLBL-1, OSW | H-3,CLBL1-P76,H-10,H-2,H-5a,H-6,H-7,OSW1,OSW3,OSW4,H-1a,H-1,OSW3,H-28,OSW4 | 13 | PIK3R1 |
| ENSCAFG00000009661 | chr38:981970-981970 | 1 | B-cell lymphoma | H-10 | 1 | PIK3C2B |
| ENSCAFG00000011212 | chr34:12650504-12650504, chr34:12660877-12660968, chr34:12674397-12674398, chr34:12674398-12674408, chr34:12674398-12674410 | 5 | B-cell lymphoma, CL-1, CLBL-1, GL-1, OSW | H-2,H-6,CL1-1,CL1-2,CLBL1-P76,CLBL1-P77,GL1,H-1,H-10,H-1a,H-28,H-3,H-6,OSW1,H-1a,H-1,H-10,H-28,H-3,H-9,H-12,H-4a,H-7 | 17 | PIK3CA |
| ENSCAFG00000017387 | chr5:33542943-33542944, chr5:33544217-33544217 | 2 | OSW, B-cell lymphoma | OSW4,H-28,OSW2 | 3 | PIK3R5 |
| ENSCAFG00000017669 | chrX:75273505-75273505 | 1 | B-cell lymphoma | H-12 | 1 | BTK |
| ENSCAFG00000017725 | chr7:48721825-48721826 | 1 | B-cell lymphoma, OSW | H-10,OSW3 | 2 | PIK3C3 |
| ENSCAFG00000019735 | chr5:62909129-62909129, chr5:62930778-62930778, chr5:62931513-62931514, chr5:62931514-62931514, chr5:62931745-62931747, chr5:62932086-62932173, chr5:62934355-62934355 | 7 | CLBL-1, OSW, B-cell lymphoma | CLBL1-P76,CLBL1-P77,CLBL1-P78,OSW1,CLBL1-P77,CLBL1-P17,CLBL1-P78,H-3,OSW3,OSW4,H-1a,CLBL1-P17,CLBL1-P77,H-10,OSW3,CLBL1-P17,OSW1,OSW2 | 11 | PIK3CD |
| ENSCAFG00000031546 | chr21:39698297-39698383 | 1 | B-cell lymphoma | H-10,H-12,H-1a,H-3 | 4 | PIK3C2A |

## Slide 66
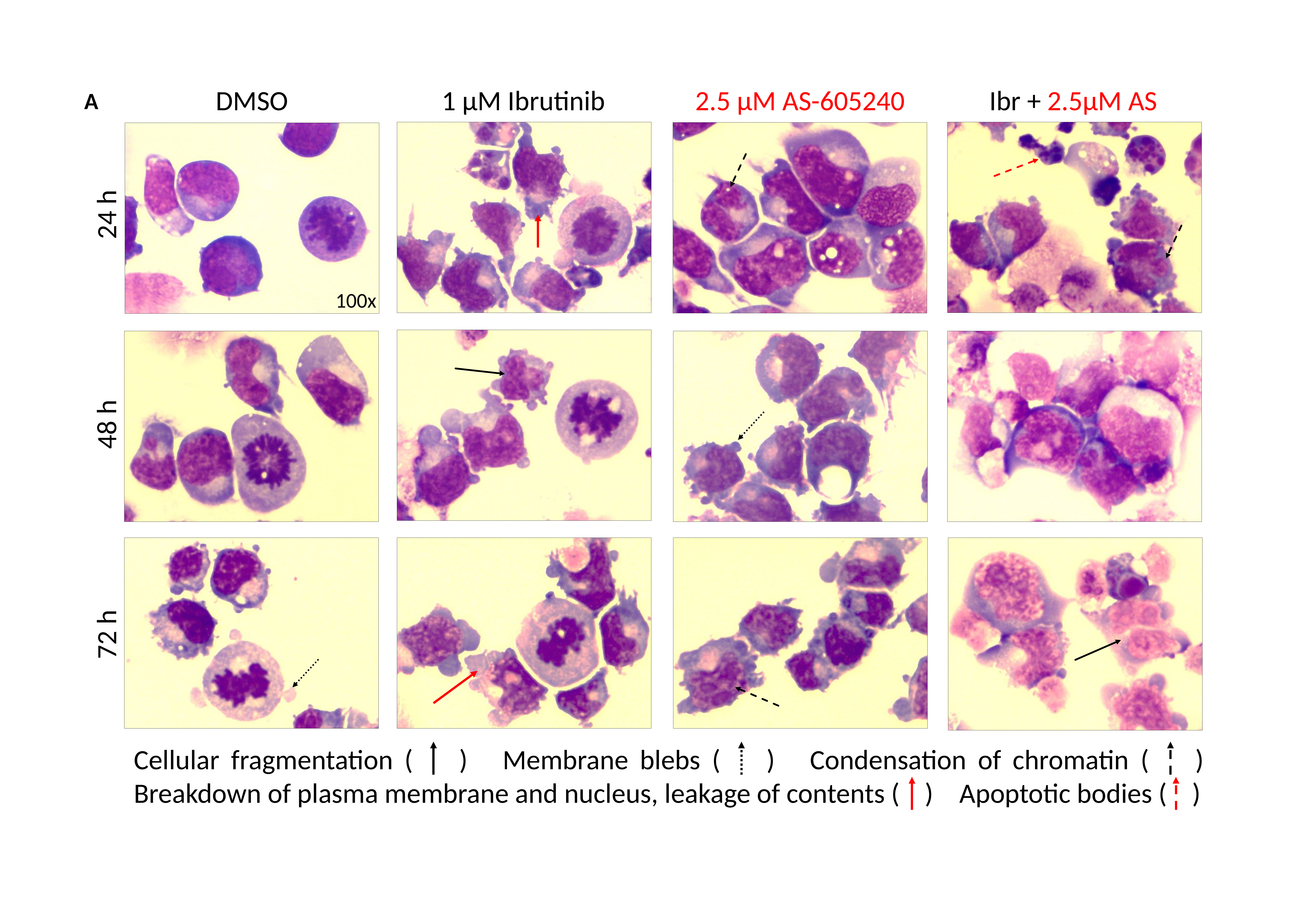

DMSO
1 µM Ibrutinib
2.5 µM AS-605240
Ibr + 2.5µM AS
A
24 h
100x
48 h
72 h
Cellular fragmentation ( ) Membrane blebs ( ) Condensation of chromatin ( ) Breakdown of plasma membrane and nucleus, leakage of contents ( ) Apoptotic bodies ( )

## Slide 67
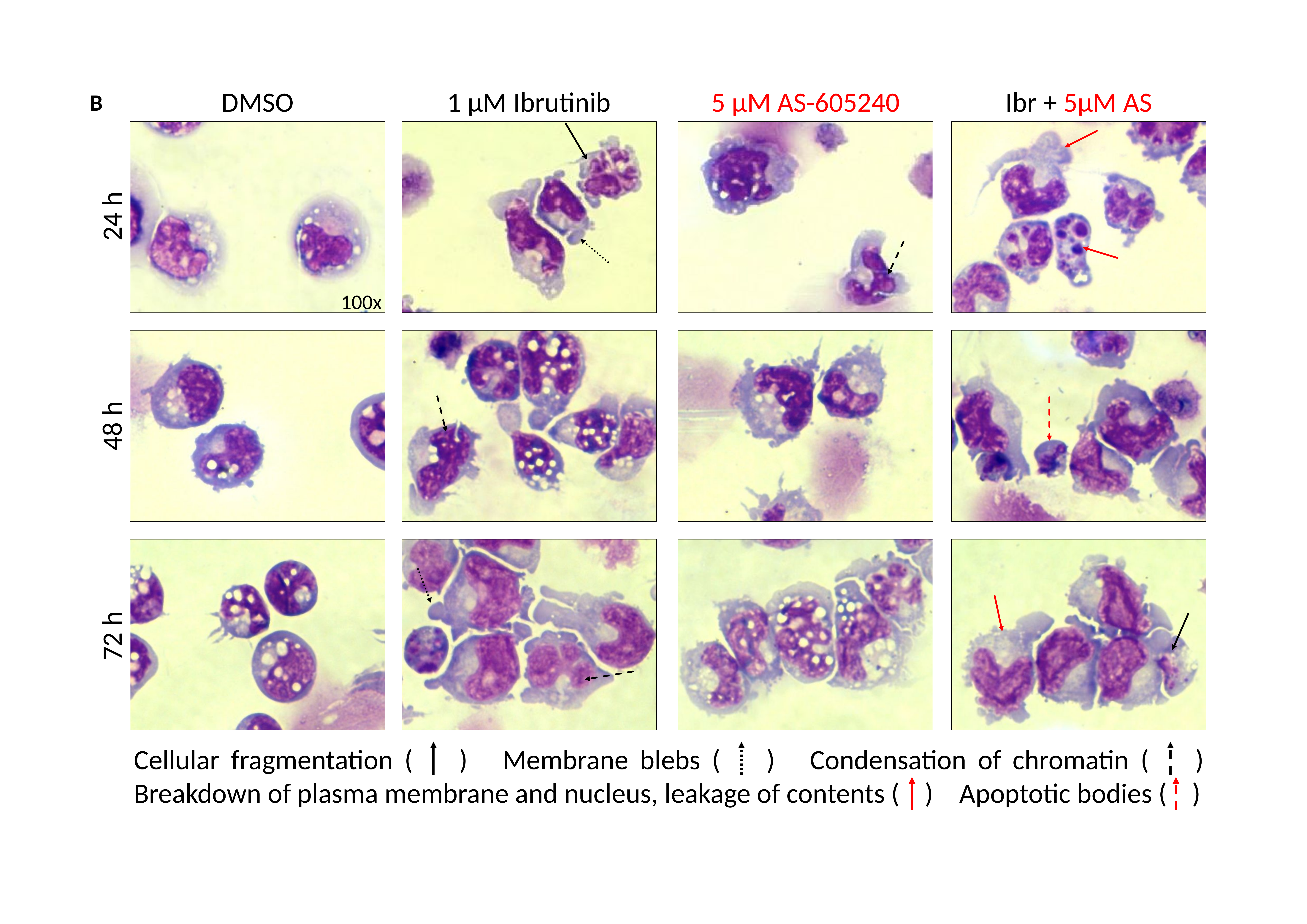

DMSO
1 µM Ibrutinib
5 µM AS-605240
Ibr + 5µM AS
100x
24 h
48 h
72 h
B
Cellular fragmentation ( ) Membrane blebs ( ) Condensation of chromatin ( ) Breakdown of plasma membrane and nucleus, leakage of contents ( ) Apoptotic bodies ( )

## Slide 68
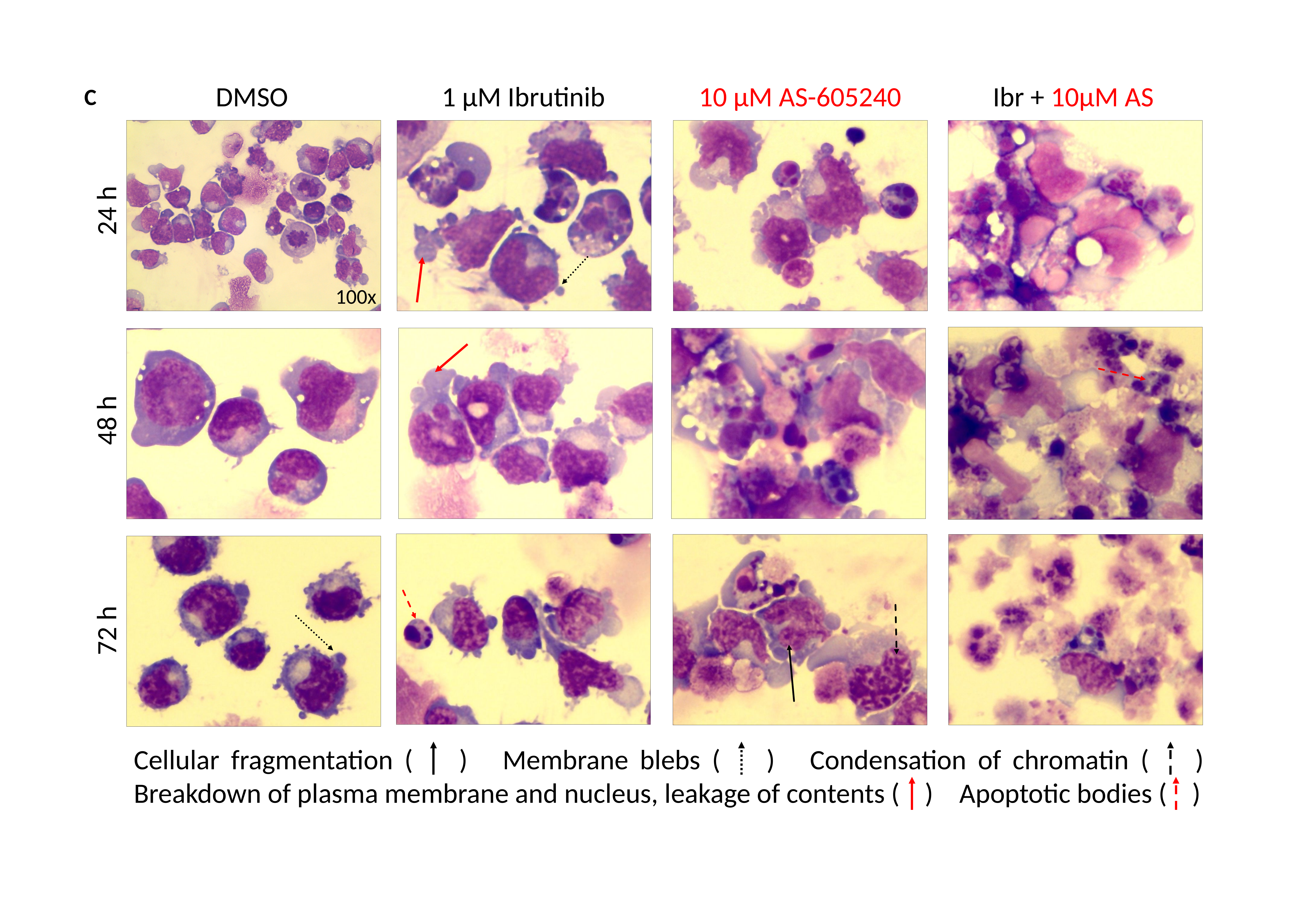

DMSO
1 µM Ibrutinib
10 µM AS-605240
Ibr + 10µM AS
C
24 h
100x
48 h
72 h
Cellular fragmentation ( ) Membrane blebs ( ) Condensation of chromatin ( ) Breakdown of plasma membrane and nucleus, leakage of contents ( ) Apoptotic bodies ( )

## Slide 69
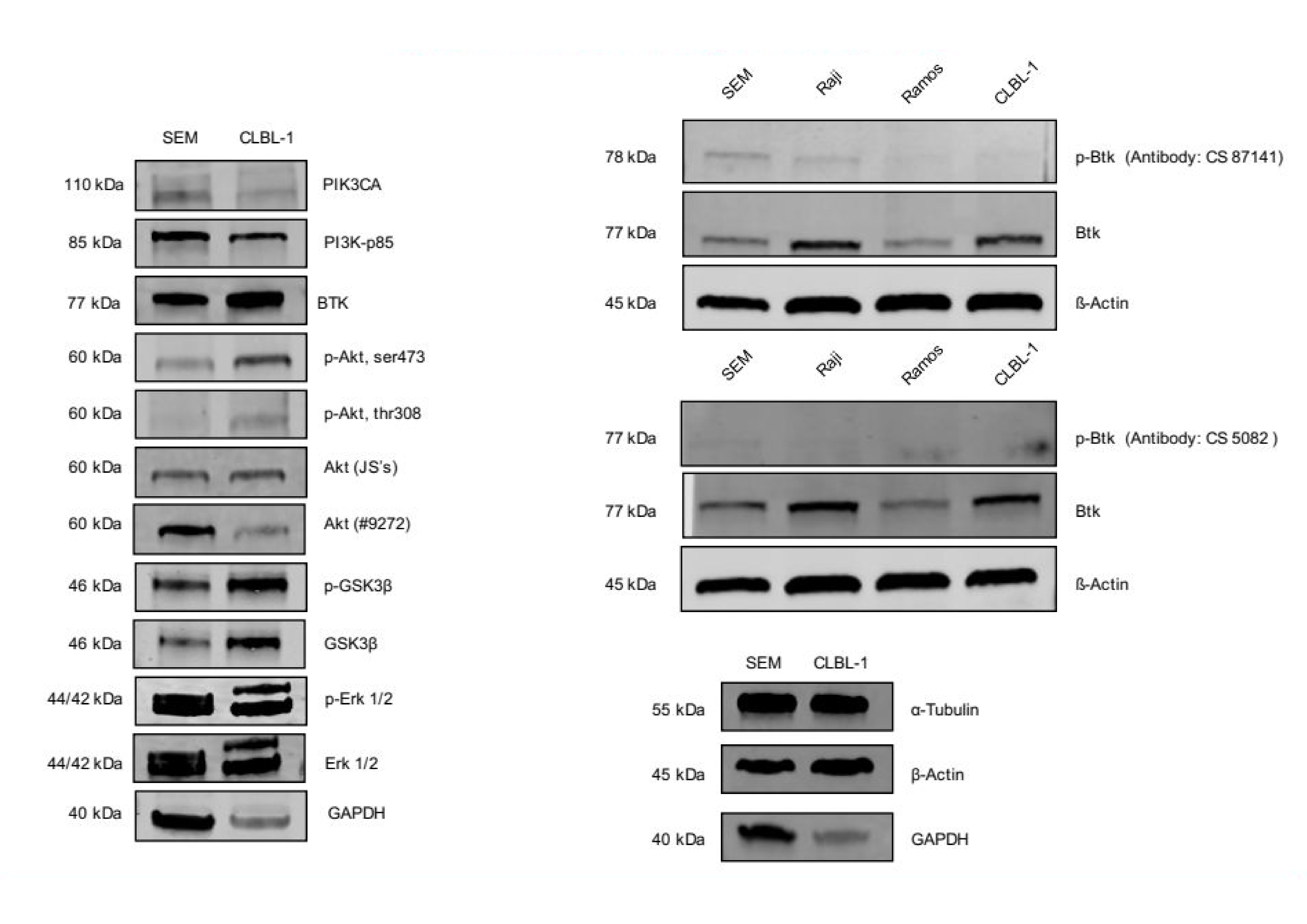

## Slide 70
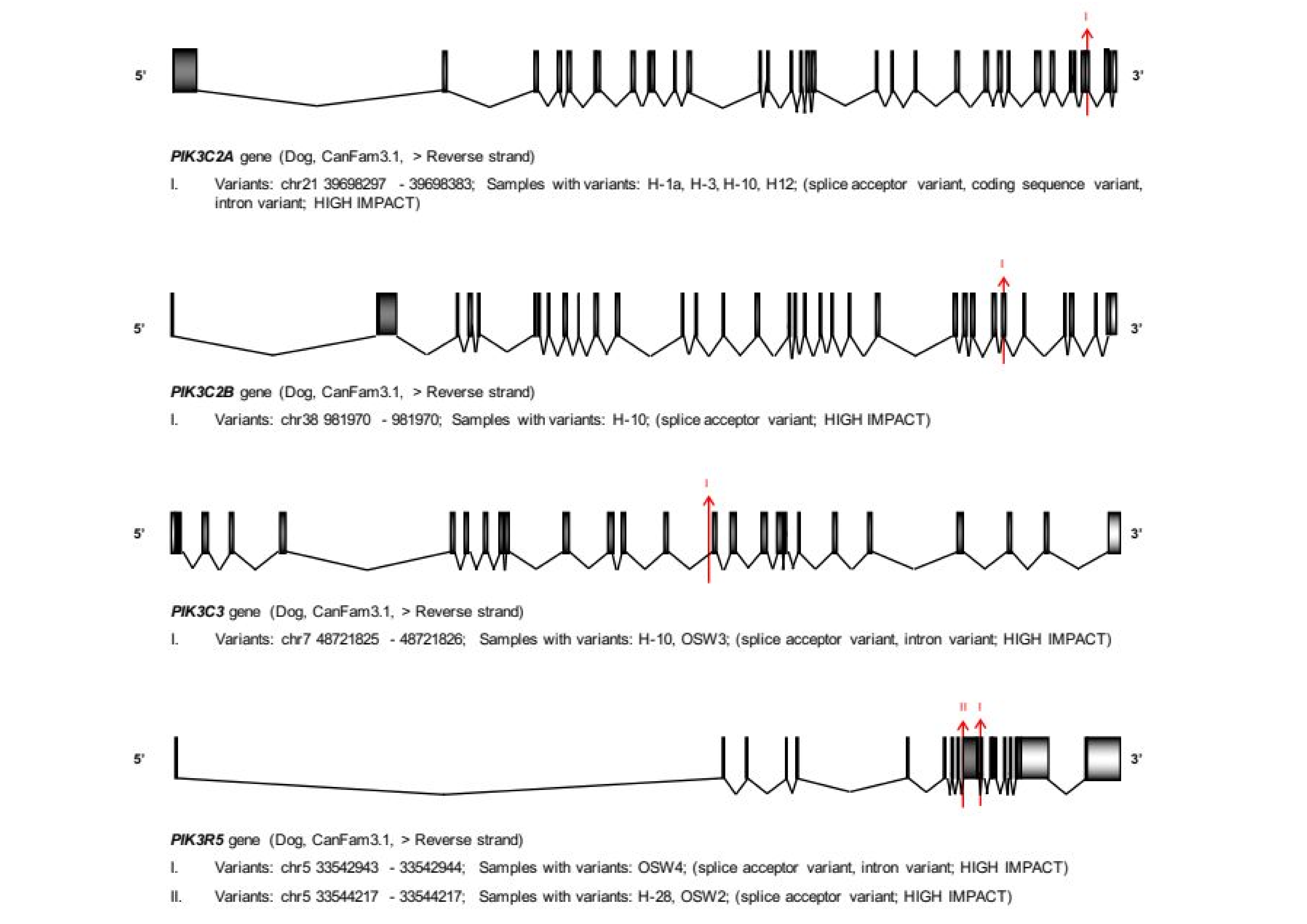

## Slide 71
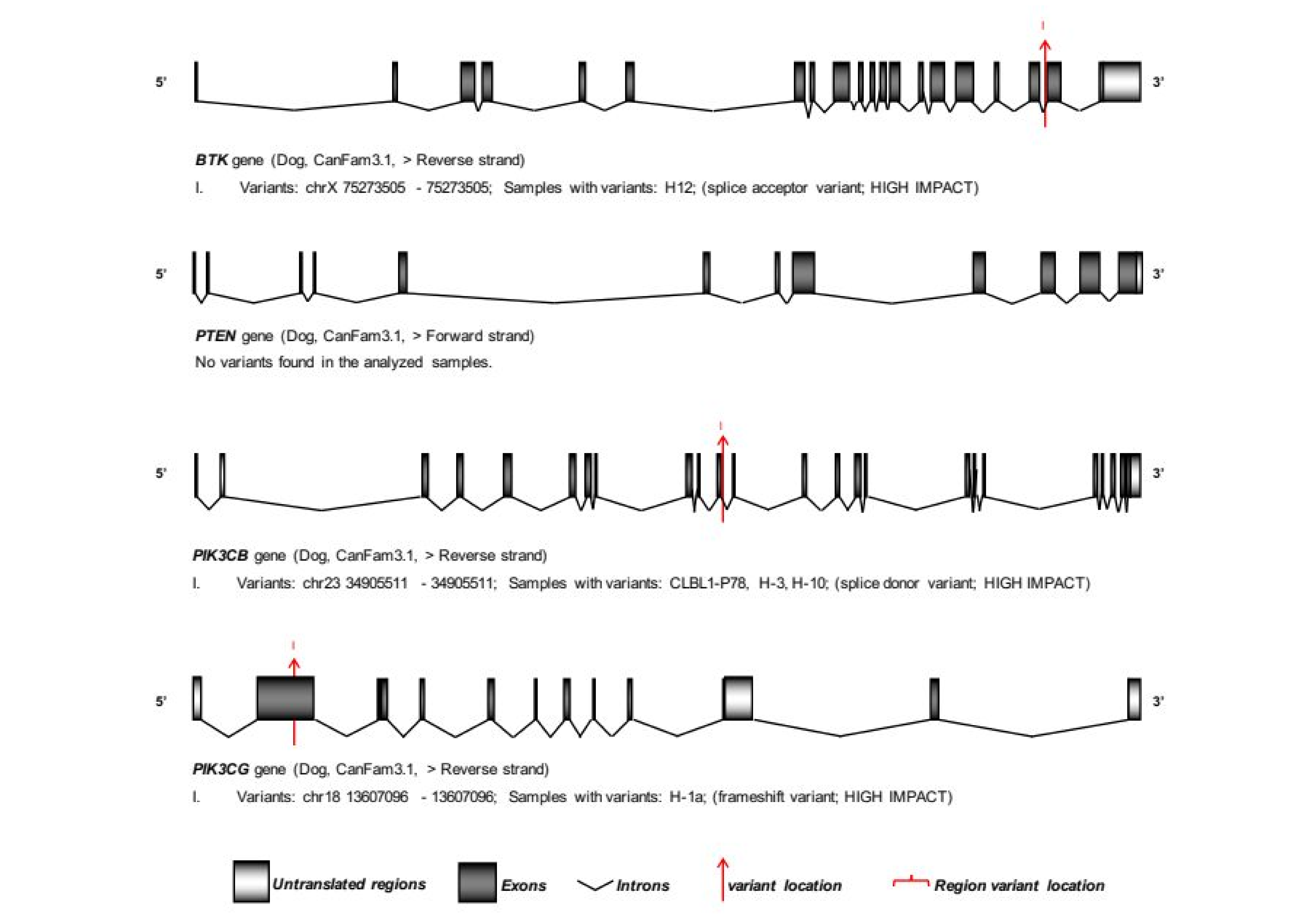

Supplement: Supplementary file 1 [file ijms-22-12673-s001.zip › ijms-1428818-supplementary.pptx]
